# Coffee drinking and cancer risk: an umbrella review of meta-analyses of observational studies

**DOI:** 10.1186/s12885-020-6561-9

**Published:** 2020-02-05

**Authors:** Long-Gang Zhao, Zhuo-Ying Li, Guo-Shan Feng, Xiao-Wei Ji, Yu-Ting Tan, Hong-Lan Li, Marc J. Gunter, Yong-Bing Xiang

**Affiliations:** 1 State Key Laboratory of Oncogene and Related Genes & Department of Epidemiology, Shanghai Cancer Institute, Renji Hospital, Shanghai Jiaotong University School of Medicine, Shanghai, China; 2 Section of Nutrition and Metabolism, International Agency for Research on Cancer, 150, cours Albert Thomas F-69372, Cedex 08 Lyon, France

**Keywords:** Coffee, Cancer, Umbrella review, Grading evidence, Observational studies

## Abstract

**Background:**

Epidemiological studies on the association between coffee intake and cancer risk have yielded inconsistent results. To summarize and appraise the quality of the current evidence, we conducted an umbrella review of existing findings from meta-analyses of observational studies.

**Methods:**

We searched PubMed, Embase, Web of Science and the Cochrane database to obtain systematic reviews and meta-analyses of associations between coffee intake and cancer incidence. For each association, we estimated the summary effect size using the fixed- and random-effects model, the 95% confidence interval, and the 95% prediction interval. We also assessed heterogeneity, evidence of small-study effects, and excess significance bias.

**Results:**

Twenty-eight individual meta-analyses including 36 summary associations for 26 cancer sites were retrieved for this umbrella review. A total of 17 meta-analyses were significant at *P* ≤ 0.05 in the random-effects model. For the highest versus lowest categories, 4 of 26 associations had a more stringent *P* value (*P* ≤ 10^− 6^). Associations for five cancers were significant in dose-response analyses. Most studies (69%) showed low heterogeneity (I^2^ ≤ 50%). Three and six associations had evidence of excessive significance bias and publication bias, respectively. Coffee intake was inversely related to the risk of liver cancer and endometrial cancer and was characterized by dose-response relationships. There were no substantial changes when we restricted analyses to meta-analysis of cohort studies.

**Conclusions:**

There is highly suggestive evidence for an inverse association between coffee intake and risk of liver and endometrial cancer. Further research is needed to provide more robust evidence for cancer at other sites.

## Background

Coffee, a beverage prepared using roasted beans from berries of the coffee plant, is one of the most widely consumed beverages in the world. Given the popularity of coffee drinking, any benefits of coffee on human health may exert substantial public health effects. Therefore, investigations of coffee intake and its potential health effects have been an area of research interest [1]. These potential links with health outcomes, especially with cancers, are appealing to the policy maker and epidemiologists, as well as to the public.

Researchers have been investigating the associations between coffee consumption and the risk of cancer for decades [2, 3]. Biochemical studies have indicated that hundreds of biologically active compounds including caffeine, flavonoids, lignans, and other polyphenols are found in roasted coffee [4]. These coffee-derived compounds have been shown to increase energy expenditure, regulate DNA repair-related genes, and inhibit the chronic inflammatory response in animal studies [5]. However, human studies from different regions or populations have generated inconsistent results. Early studies on the impacts of coffee mainly focused on bladder cancer, which indicated a higher risk of bladder cancer based on case-control studies [6, 7]. Studies conducted in the past 30 years have suggested a potential relation between coffee consumption and cancers of the liver, endometrium, and melanoma [8–11]. Even though numerous findings have accumulated to support the benefits of recommendations on coffee drinking daily habits for cancer prevention, there is no convincing evidence because of inconsistent results across studies and issues with data quality. Previous studies focused on all kinds of health outcomes rather than only cancer at various sites [12, 13]. It is therefore important to summarize the current epidemiological evidence and appraises its quality based on a comprehensive criteria of grading evidence.

Umbrella reviews offer the possibility to understand the strength of evidence and the extent of potential biases for the associations between coffee and cancer incidence [14]. In this report, we summarized the epidemiological evidence on associations between coffee consumption and the risk of developing any type of cancer. We described the magnitude, direction, and significance of the observed associations between coffee intake and cancer incidence. We also evaluated whether there is evidence of biases in the findings and identify the most robust associations which have fewer potential biases.

## Methods

We had registered this protocol within the PROSPERO database for systematic reviews and meta-analyses (registration number: CRD42017084381). The protocol has been designed and reported according to the reporting guidance provided in the Preferred Reporting Items for Systematic Reviews and Meta-Analyses Protocols (PRISMA-P) statement [15].

### Literature search, study selection, and data extraction

Three researchers independently searched the PubMed, Embase, Web of Science and the Cochrane database of systematic reviews from inception to February 2019 for meta-analyses or systematic reviews of observational studies investigating the association between coffee consumption and risk of any developing cancer. We screened the titles and abstracts of all articles and then examined in detail and screened for applicability through full text. Further information on screening, selection procedure, and data collection were provided in the Additional file.

### Data analysis

We carried out a descriptive analysis of systematic reviews. The data from each systematic review and findings based on methodological quality were used to build evidence tables. We reestimated the summary effect size and its 95% confidence interval (95%CI) [16]. In the sensitivity analysis, we reselected meta-analysis with the largest number of only cohort studies from the database. We evaluated heterogeneity by estimating the variance between studies using Cochran’s Q test and the I^2^ statistic [17, 18]. We also estimated the 95% prediction interval (95%PI) [19]. An indication of small study effects was evaluated based on the Egger’s test [20]. We assessed excess significance bias by evaluating whether the observed number of studies with nominally statistically significant results was different from the expected number of studies with statistically significant results [21]. Details can be found in the Additional file 1.

### Grading the evidence

We categorized the associations between measures of coffee consumption and cancers into strong, highly suggestive, suggestive, weak, or no association depending on the strength and validity of the evidence, such as *P* value of the random-effects model, total cases, I^2^ statistic of heterogeneity, 95%PI, small study effects, and excess significance bias [22–24]. Details were provided in Table 3.

All statistical analyses were performed using Stata version 14.0 (Stata Corp LP, College Station, TX, USA). A two-sided *P* value less than 0.05 was considered as reaching statistical significance if not specified.

## Results

### Literature selection

**Fig. 1:**
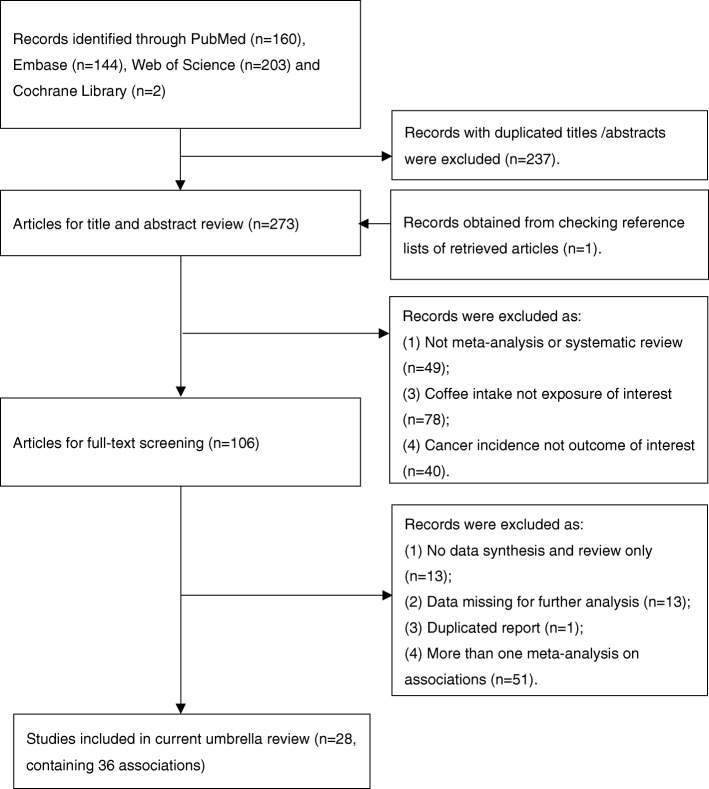
Flow diagram of the selection process of meta-analyses on coffee intake and cancers

The flow chart of article selection was provided in Fig. 1. Briefly, we identified 272 articles after removing the duplicates. We provided a list (online Additional file 1: Table S1) for excluded studies that did not meet our inclusion criteria.

After two steps of careful reviews, we identified 28 original meta-analyses that investigated associations between coffee intake and cancer incidence. Among these studies, we finally selected 36 non-overlapped associations from 28 independent original studies. Twenty-six of these used the highest versus lowest intake of coffee drinking in a categorical and the remainder analyzed the data in a dose-response analysis.

### Characteristics of meta-analyses included in this umbrella review

Table 1 summarizes these 36 independent associations including 26 cancer sites. The median number of individual studies for each cancer was 15, ranging from 4 to 54. The number of cases ranged from 255 to 104,770. All except for three comparisons (acute myelogenous leukemia, biliary tract cancer, and glioma) included more than 1000 cases in the meta-analyses. Of 448 individual studies for the highest versus lowest comparison, 268 (59.8%) were case-control studies and 179 (40.0%) were cohort studies and one was a cross-sectional study. Of 144 individual studies for dose-response analysis, 40 (27.8%) were case-control studies and 104 (72.2%) were cohort studies.

**Table 1:** Description of 36 associations of coffee intake and cancer incidence included in umbrella review

					Summary relative risk (95% CI)			*P* value		
Author, year	Cancer type	Unit of comparison	No of studies	No. of cases	Fixed-effects model	Random-effects model	Largest study	Fixed-effects model	Random-effects model	95% PI
Thomopoulos, 2015 [20]	Acute lymphocytic leukemia	H/L	7	2453	1.43 (1.22, 1.68)	1.44 (1.21, 1.71)	1.30 (1.00, 1.70)	9.28E-06	2.9E-05	(1.09, 1.91)
Thomopoulos, 2015 [20]	Acute myelogenous leukemia	H/L	6	333	1.76 (1.17, 2.63)	1.78 (0.87, 3.65)	2.40 (1.30, 4.30)	0.006	0.115	(0.17, 18.39)
Godos, 2017 [4]	Biliary tract cancer	H/L	5	726	0.91 (0.69, 1.20)	0.91 (0.69, 1.20)	0.94 (0.64, 1.37)	0.006	0.115	(0.58, 1.43)
Wu, 2015 [24]	Bladder cancer	H/L	30	16,172	1.29 (1.19, 1.39)	1.35 (1.20, 1.50)	1.00 (0.83, 1.21)	8.89E-11	1.54E-07	(0.90, 2.00)
Jiang, 2013 [9]	Breast cancer	H/L	34	56,541	0.97 (0.93, 1.01)	0.97 (0.93, 1.01)	0.98 (0.91, 1.07)	0.089	0.089	(0.93, 1.01)
Gan, 2017 [3]	Colon cancer	H/L	16	13,853	0.91 (0.84, 0.98)	0.92 (0.83, 1.02)	0.92 (0.80, 1.06)	0.011	0.097	(0.72, 1.18)
Li, 2013 [13]	Colorectal cancer	H/L	40	25,965	0.89 (0.85, 0.94)	0.88 (0.81, 0.96)	0.90 (0.80, 1.02)	1.37E-05	0.003	(0.59, 1.30)
Lukic, 2018 [8]	Endometrial cancer	H/L	19	13,812	0.79 (0.74, 0.84)	0.75 (0.70, 0.82)	0.92 (0.82, 1.03)	2.67E-14	2.90E-11	(0.60, 0.93)
Zheng, 2013 [28]	Esophageal cancer	H/L	14	3575	0.90 (0.81, 1.00)	0.84 (0.72, 0.98)	1.13 (0.92, 1.37)	0.047	0.028	(0.55, 1.29)
Botelho, 2006 [1]	Gastric cancer	H/L	23	5611	0.96 (0.88, 1.06)	0.97 (0.86, 1.09)	0.93 (0.72, 1.21)	0.422	0.593	(0.67, 1.40)
Malerba, 2013 [15]	Glioma	H/L	6	255	1.01 (0.83, 1.22)	1.01 (0.83, 1.22)	0.98 (0.67, 1.41)	0.943	0.943	(0.77, 1.32)
Wijarnpreecha, 2017 [23]	Kidney cancer	H/L	22	11,087	1.00 (0.92, 1.09)	0.99 (0.89, 1.11)	1.33 (1.07, 1.66)	0.971	0.909	(0.71, 1.38)
Ouyang, 2014 [16]	Laryngeal cancer	H/L	8	2596	1.05 (0.94, 1.18)	1.18 (0.87, 1.59)	0.90 (0.80, 1.10)	0.397	0.284	(0.47, 2.93)
Thomopoulos, 2015 [20]	Leukemia	H/L	6	2303	1.41 (1.19, 1.66)	1.57 (1.16, 2.11)	1.10 (0.90, 1.50)	5.27E-05	0.003	(0.69, 3.56)
Sang, 2013 [17]	Liver cancer	H/L	16	3622	0.50 (0.42, 0.59)	0.48 (0.40, 0.58)	0.70 (0.50, 1.00)	2.77E-16	8.84E-15	(0.35, 0.67)
Galarraga, 2016 [2]	Lung cancer	H/L	21	19,892	1.08 (1.06, 1.11)	1.26 (1.13, 1.41)	1.04 (1.01, 1.07)	1.84E-11	4.7E-05	(0.82, 1.93)
Han, 2016 [6]	Lymphoma	H/L	7	1513	1.05 (0.90, 1.23)	1.08 (0.76, 1.53)	1.11 (0.90, 1.37)	0.551	0.659	(0.38, 3.05)
Yew, 2016 [27]	Melanoma	H/L	11	3787	0.76 (0.69, 0.84)	0.75 (0.65, 0.87)	0.80 (0.69, 0.93)	1.39E-08	0.000151	(0.50, 1.12)
Vaseghi, 2016 [22]	Nonmelanoma	H/L	6	104,770	0.86 (0.80, 0.93)	0.87 (0.78, 0.98)	0.83 (0.75, 0.92)	0.000111	0.021	(0.67, 1.13)
Li, 2016 [11]	Oral cancer	H/L	15	5021	0.63 (0.57, 0.71)	0.63 (0.52, 0.75)	0.51 (0.40, 0.64)	7.3E-16	3.79E-07	(0.36, 1.09)
Miranda, 2017 [15]	Oral/Pharyngeal cancer	H/L	17	5151	0.72 (0.64, 0.82)	0.69 (0.57, 0.84)	0.60 (0.45, 0.80)	5.26E-07	0.000281	(0.37, 1.29)
Steevens, 2007 [18]	Ovarian cancer	H/L	15	5479	1.11 (0.98, 1.27)	1.18 (0.97, 1.44)	0.93 (0.69, 1.26)	0.115	0.101	(0.63, 2.19)
Turati, 2012 [21]	Pancreatic cancer	H/L	54	10,594	1.14 (1.05, 1.24)	1.13 (0.99, 1.29)	0.95 (0.73, 1.23)	0.002	0.063	(0.59, 2.16)
Xia, 2017 [26]	Prostate cancer	H/L	27	42,399	1.02 (0.96, 1.07)	1.07 (0.96, 1.18)	0.94 (0.87, 1.02)	0.585	0.228	(0.74, 1.53)
Gan, 2017 [3]	Rectal cancer	H/L	15	6200	1.07 (0.97, 1.18)	1.06 (0.95, 1.19)	1.20 (1.00, 1.44)	0.185	0.285	(0.86, 1.31)
Han, 2017 [5]	Thyroid cancer	H/L	7	1039	0.91 (0.74, 1.12)	0.91 (0.74, 1.12)	1.00 (0.68, 1.48)	0.37	0.37	(0.70, 1.19)
Li, 2013 [12]	Breast cancer	Per 1 cup	24	46,812	0.99 (0.98, 1.00)	0.99 (0.98, 1.00)	0.99 (0.98, 1.01)	0.009	0.009	(0.98, 1.00)
Gan, 2017 [3]	Colon cancer	Per 1 cup	15	13,650	0.98 (0.97, 0.99)	0.98 (0.97, 1.00)	0.97 (0.95, 0.99)	0.0006	0.019	(0.95, 1.02)
Gan, 2017 [3]	Colorectal cancer	Per 1 cup	17	22,037	0.99 (0.98, 1.00)	0.99 (0.98, 1.01)	0.97 (0.96, 0.99)	0.01	0.292	(0.96, 1.03)
Huang, 2013 [7]	Endometrial cancer	Per 1 cup	7	3571	0.94 (0.91, 0.96)	0.93 (0.90, 0.97)	0.94 (0.90, 0.97)	6.63E-07	0.000321	(0.85, 1.02)
Xie, 2014 [26]	Gastric cancer	Per 1 cup	9	2344	1.00 (0.98, 1.03)	1.02 (0.97, 1.06)	0.97 (0.93, 1.01)	0.806	0.482	(0.90, 1.15)
Malerba, 2013 [14]	Glioma	Per 1 cup	4	1493	1.00 (0.97, 1.03)	1.00 (0.96, 1.05)	1.00 (0.96, 1.05)	0.927	0.939	(0.85, 1.19)
Kennedy, 2017 [10]	Liver cancer	Per 1 cup	17	4730	0.84 (0.82, 0.86)	0.81 (0.77, 0.85)	0.90 (0.86, 0.95)	2.69E-36	1.2E-16	(0.68, 0.95)
Tang, 2010 [19]	Lung cancer	Per 1 cup	9	3862	1.07 (1.04, 1.11)	1.07 (1.02, 1.12)	1.10 (1.03, 1.17)	3E-05	0.006	(0.95, 1.21)
Turati, 2012 [21]	Pancreatic cancer	Per 1 cup	28	6534	1.02 (1.00, 1.03)	1.03 (0.99, 1.06)	1.04 (1.00, 1.08)	0.047	0.12	(0.90, 1.17)
Gan, 2017 [3]	Rectal cancer	Per 1 cup	14	6134	1.01 (0.99, 1.03)	1.01 (0.99, 1.03)	0.95 (0.86, 1.07)	0.263	0.211	(0.98, 1.05)

Abbreviation: H/L The highest intake vs. lowest intake of coffee

Reference was provided in Additional file 1: Table S3

For associations with more than one published meta-analysis, there was an agreement in general on the direction, magnitude, and significance of the summary associations given the different numbers of studies included. Details were provided in the online Additional file 1: Table S1.

### Evidence summary

The summary random-effects estimates were significant at *P* ≤ 0.05 in 17 meta-analyses (39%). For the highest versus lowest categories (H/L), four of 26 associations had a more stringent *P* value (*P* ≤ 10^− 6^). Overall, coffee intake was associated with incident bladder cancer, endometrial cancer, liver cancer, and oral cancer in the highest versus lowest analyses. Coffee was associated with five cancers in dose-response analyses (cancers of breast, colon, endometrium, liver, and lung). There is a slight difference between the random-effects model and fixed-effects model. The studies with the smallest standard error of each association suggested that 16 of 36 were significant at *P* = 0.05.

**Fig. 2:**
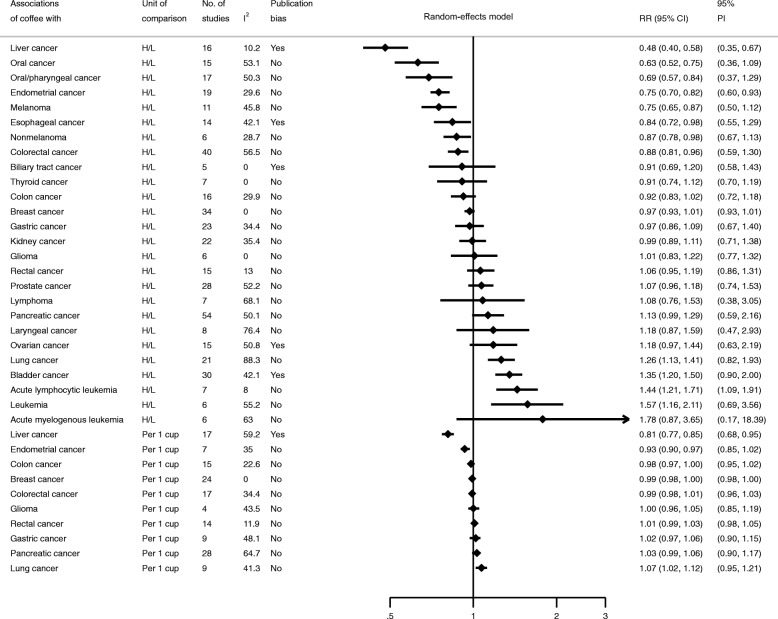
Summary estimates with 95% confidence and prediction intervals from 36 associations of coffee and cancer. H/L, the highest versus lowest intake of coffee; RR, relative risk; PI, prediction intervals. RRs and 95%CIs displayed were calculated from random-effects models.

The Q test showed significant heterogeneity (*P* ≤ 0.10) for 11 (31%, 11/36) meta-analyses (Table 2 and Fig. 2). Most studies (69%, 25/36) showed low heterogeneity (I^2^ ≤ 50%). The associations showed substantial heterogeneity (I^2^ > 75%) included laryngeal cancer (H/L) and lung cancer (H/L). Only three associations (acute lymphocytic leukemia, endometrial cancer, and liver cancer) had a 95% PI that excluded the null value. Details were provided in the Additional file 2.

**Table 2:** Evaluation of bias and heterogeneity in 36 associations of coffee intake with cancer incidence

Author, year	Cancer type	Unit of comparison	I^2^ (95%CI)	*P* for Q*	Egger’s *P* value	Observed^a^	Expected^a^	*P* for TES^b^
Thomopoulos, 2015 [20]	Acute lymphocytic leukemia	H/L	8 (0, 73)	0.368	0.309	2	1.24	–
Thomopoulos, 2015 [20]	Acute myelogenous leukemia	H/L	63 (2, 86)	0.029	0.9	1	1.16	0.444
Godos, 2017 [4]	Biliary tract cancer	H/L	0 (0, 79)	0.849	0.073	0	0.42	–
Wu, 2015 [24]	Bladder cancer	H/L	42 (11, 63)	0.009	0.014	9	10.42	–
Jiang, 2013 [9]	Breast cancer	H/L	0 (0, 39)	0.485	0.374	4	5.64	–
Gan, 2017 [3]	Colon cancer	H/L	30 (0, 62)	0.124	0.699	3	3.14	–
Li, 2013 [13]	Colorectal cancer	H/L	57 (38, 70)	< 0.001	0.419	11	10.27	–
Lukic, 2018 [8]	Endometrial cancer	H/L	29 (0, 60)	0.132	0.326	8	3.17	0.786
Zheng, 2013 [28]	Esophageal cancer	H/L	42 (0, 69)	0.049	0.038	3	4.88	0.008
Botelho, 2006 [1]	Gastric cancer	H/L	34 (0, 61)	0.055	0.695	3	5.13	–
Malerba, 2013 [15]	Glioma	H/L	0 (0, 75)	0.479	0.135	0	0.85	–
Wijarnpreecha, 2017 [23]	Kidney cancer	H/L	35 (0, 62)	0.052	0.882	2	8.51	–
Ouyang, 2014 [16]	Laryngeal cancer	H/L	76 (53, 88)	< 0.001	0.329	2	2.83	–
Thomopoulos, 2015 [20]	Leukemia	H/L	55 (0, 82)	0.048	0.128	2	2.34	–
Sang, 2013 [17]	Liver cancer	H/L	10 (0, 47)	0.337	0.05	7	4.24	0.118
Galarraga, 2016 [2]	Lung cancer	H/L	88 (84, 92)	< 0.001	0.104	9	9.29	–
Han, 2016 [6]	Lymphoma	H/L	68 (30, 86)	0.004	0.775	2	2.32	–
Yew, 2016 [27]	Melanoma	H/L	46 (0, 73)	0.048	0.824	5	2.7	0.107
Vaseghi, 2016 [22]	Nonmelanoma	H/L	29 (0, 71)	0.22	0.512	1	1.28	–
Li, 2016 [11]	Oral cancer	H/L	53 (16, 74)	0.008	0.685	7	4.89	0.245
Miranda, 2017 [15]	Oral/Pharyngeal cancer	H/L	50 (13, 72)	0.009	0.287	6	4.86	0.537
Steevens, 2007 [18]	Ovarian cancer	H/L	51 (11, 73)	0.012	0.047	3	4.57	–
Turati, 2012 [21]	Pancreatic cancer	H/L	50 (32, 64)	< 0.001	0.876	12	14.86	–
Xia, 2017 [26]	Prostate cancer	H/L	52 (32, 71)	0.001	0.229	4	6.63	–
Gan, 2017 [3]	Rectal cancer	H/L	13 (0, 51)	0.308	0.822	1	3.13	–
Han, 2017 [5]	Thyroid cancer	H/L	0 (0, 71)	0.591	0.746	0	1	–
Li, 2013 [12]	Breast cancer	Per 1 cup	0 (0, 45)	0.796	0.619	0	3.30	–
Gan, 2017 [3]	Colon cancer	Per 1 cup	23 (0, 58)	0.203	0.818	3	3.00	0.999
Gan, 2017 [3]	Colorectal cancer	Per 1 cup	34 (0, 63)	0.081	0.434	3	4.80	–
Huang, 2013 [7]	Endometrial cancer	Per 1 cup	35 (0, 73)	0.161	0.274	3	1.30	0.098
Xie, 2014 [26]	Gastric cancer	Per 1 cup	48 (0, 76)	0.051	0.198	1	2.66	–
Malerba, 2013 [14]	Glioma	Per 1 cup	44 (0, 81)	0.15	0.961	0	0.83	–
Kennedy, 2017 [10]	Liver cancer	Per 1 cup	59 (30, 76)	0.001	0.00005093	13	7.22	0.005
Tang, 2010 [19]	Lung cancer	Per 1 cup	41 (0, 73)	0.092	0.499	3	2.21	0.543
Turati, 2012 [21]	Pancreatic cancer	Per 1 cup	65 (47, 76)	< 0.001	0.326	4	8.67	–
Gan, 2017 [3]	Rectal cancer	Per 1 cup	12 (0, 50)	0.323	0.383	0	3.15	–

Abbreviation: *H/L* The highest intake vs. lowest intake of coffee

Reference was provided in Additional file 1: Table S3

* *P* value from Cochran’s Q test for heterogeneity.

^a^ Observed and expected number of significant studies using effect of largest study (smallest standard error) of each meta-analysis as plausible effect size

^b^ *P* value from test for excess significance bias. All statistical tests are two sided

### Excess significance test and publication bias

From Table 2, the evidence for excess significance using the largest study estimate as the plausible effect size was present for three outcomes, including liver cancer (per 1 cup), endometrial cancer (per 1 cup) and esophageal cancer (H/L). Using summary estimates of fixed or random-effects as plausible effect sizes gave similar results. There were 21 associations that included 10 or more studies. Six of 36 had evidence of publication bias using Egger’s test.

### Evaluating the evidence

Based on the above analyses, the association of childhood acute lymphocytic leukemia was supported by strong evidence in the current analyses (Table 3). For the highest versus lowest categories, six meta-analyses (23.1%, 6/26) were supported by highly suggestive evidence for an association. Coffee intake was inversely related to the risk of endometrial cancer, liver cancer, melanoma, oral cancer, and oral/pharyngeal cancer while positively related to the risk of bladder cancer. The association between coffee and lung cancer was categorized as suggestive evidence. Four meta-analyses (15%, 4/26) were supported by weak evidence including colorectal cancer, nonmelanoma, esophageal cancer, and leukemia. For dose-response analysis, only liver and endometrial cancers were judged as highly suggestive evidence. Three cancers were considered as having weak evidence in relation to coffee drinking including cancers of the colon, breast (women), and lung.

**Table 3:** Summary of evidence grading for meta-analyses associating coffee intake with cancer incidence Level of evidence

	Criteria used	Decreased risk	Increased risk
Strong	*P* in random-effects model < = 0.001 Number of cases > 1000 I^2^ < = 50% 95% predictive intervals exclude the null value Small study effects *P* > 0.1 Excess significance bias *P* > 0.1	None	Acute lymphocytic leukemia (H/L)
Highly suggestive	*P* in random-effects model < = 0.001Number of cases > 1000 I^2^ < = 75%	Endometrial cancer (H/L); Endometrial cancer (Per 1 cup); Liver cancer (H/L); Liver cancer (Per 1 cup); Melanoma (H/L); Oral cancer (H/L); Oral/pharyngeal cancer (H/L)	Bladder (H/L)
Suggestive	*P* in random-effects model < = 0.001Number of cases > 500	None	Lung cancer (H/L)
Weak	*P* in random-effects model <= 0.05	Breast cancer (Per 1 cup); Colon cancer (Per 1 cup); Colorectal cancer (H/L); Esophageal cancer (H/L); Nonmelanoma (H/L)	Leukemia (H/L); Lung (Per 1 cup)
No association	*P* in random-effects model > 0.05	Pancreatic cancer (H/L); Breast cancer (H/L); Colon cancer (H/L); Ovarian cancer (H/L); Acute myelogenous leukemia (H/L); Pancreatic cancer (Per 1 cup); Rectal cancer (Per 1 cup); Kidney cancer (H/L); Laryngeal cancer (H/L); Rectal cancer (H/L); Colorectal cancer (Per 1 cup); Thyroid cancer (H/L); Prostate cancer (H/L); Gastric cancer (Per 1 cup); Gastric cancer (H/L); Lymphoma (H/L); Glioma (Per 1 cup); Glioma (H/L); Biliary tract cancer (H/L).	

*H/L* Highest intake vs. lowest intake of coffee

The AMSTAR 2 instrument adopted the rating process based on the identification of critical domains and distinguished the reviews into four categories: high, moderate, low, and extremely low. Twenty-six out of 28 reviews (93%) scored as extremely low (online Additional file 1: Table S3). The low scores may be due to two main domains. First, none of the included reviews contained an explicit statement or published protocol prior to the conduct of the review. Second, none of the reviews searched the grey literature database.

### Secondary analysis

When we included a meta-analysis of prospective cohort studies only, our main results were robust (online Additional file 1: Tables S4, S5, and S6). We still found highly suggestive evidence for the associations for liver and endometrial cancers. Several associations mainly based on case-control studies were not evaluated in sensitivity analyses. Associations for melanoma and lung cancer were considered as suggestive evidence. Other associations found in the main analysis were classified into weak evidence.

## Discussion

In this umbrella review, we evaluated associations between coffee intake and 26 different cancer sites including 364,749 cancer cases. With the detailed evaluation of bias in the literature, our studies revealed that coffee intake was inversely associated with five cancer sites, namely, endometrial cancer, liver cancer, melanoma, oral cancer, and oral/pharyngeal cancer. For endometrial and liver cancers, there were robust evidences for a dose-dependent association. Higher coffee intake was associated with an increased risk of childhood acute lymphocytic leukemia and bladder cancer. When we used a meta-analysis of prospective cohort studies, only associations for liver and endometrial cancers were further confirmed.

The International Agency for Research on Cancer (IARC) and the World Cancer Research Fund (WCRF) have judged the evidence of coffee intake and risk of cancer recently [25, 26]. Their latest evaluation reported an inverse association with coffee drinking for liver and endometrial cancers. Consistent with this, the WCRF summarized the main findings from the Continuous Update Project (CUP) dose-response meta-analyses of cohort studies on coffee drinking and cancer risk reporting that it was probable that coffee intake was inversely associated with liver and endometrial cancers with low heterogeneity. The evidence was deemed suggestive for the risk of cancers of the mouth, pharynx and larynx, and of skin cancer. However, for other cancer sites, the evidence was too limited to draw a firm conclusion.

Our umbrella review of the existing evidence supports an inverse association between coffee intake and the risk of liver and endometrial cancers, which is similar to other studies [12, 27]. Previous studies only described the associations between coffee and liver and endometrial cancer and did not comprehensively evaluate the potential heterogeneity and bias. Our studies indicated there were still some questions before achieving a determinative conclusion. There was evidence of small study effects and excess significance bias for liver cancer. When we re-analyzed in meta-analyses of cohort studies, the results still tended to be affected by publication bias and excess significance bias. The association for endometrial cancer also had evidence of excess significance bias. These biases may degrade the evidence and lead to cautious and prudent conclusions.

Our studies indicated higher coffee intake was inversely associated with melanoma and oral/pharyngeal cancer. However, these associations are mostly based on case-control studies that naturally tended to be affected by recall bias and selection bias. When we restricted to meta-analyses of cohort studies, the number of cases was less than 1000 for melanoma making it challenging to interpret the findings. More data from cohort studies will be needed to draw more firm conclusions for this cancer. For oral/pharyngeal cancer, substantial heterogeneity was found between cohort studies even though only four cohort studies were combined for oral cancer and six for oral/pharyngeal cancer. Similar to melanoma, associations of coffee with these rare cancers needs to be investigated in more cohorts or pooled studies.

The associations between coffee and cancers of bladder and lung have been highly debated [13, 28]. In early reports by IARC, it was reported that there was a suggestive positive link between these two cancers with coffee based on case-control studies [29]. The current study indicated stronger evidence when included meta-analysis of all observational studies. However, these associations were categorized into weak or no evidence in meta-analyses of cohort studies. The recall bias and confounding effect of tobacco smoking may play a role in these observed associations. Recently, some debates are ignited about the acrylamide formed early in the roasting process of coffee beans, a carcinogen in both animals and human [30]. However, we did not have enough evidence to attribute the positive associations between coffee and these two types of cancers to the acrylamide because such a low exposure of acrylamide in coffee is unlikely to cause any cancers in human body [31, 32].

Our study also indicated high coffee consumption during pregnancy was associated with acute lymphocytic leukemia in childhood based on a meta-analysis of six case-control studies. Even though the evidence is categorized as convincing evidence, we still need more cohort studies to illustrate the association between maternal coffee intake and childhood leukemia. For colon and breast cancers, if there is any association, coffee drinking may have a very small effect, which means these associations need a larger sample of population to be confirmed. For nonmelanoma and esophageal cancer, these associations were present mainly in case-control studies [33, 34]. Therefore, more prospective studies are needed for a further conclusion.

For possible associations of coffee intake with the risk of cancer, the biological mechanism remains unclear. Numerous studies indicated that coffee drinking provides exposure to a range of biologically active chemicals, including caffeine and phenolic compounds that may impact health through various mechanisms, such as antioxidants, insulin sensitivity, liver function, and chronic inflammation [4, 35]. However, these benefits have not been obtained in randomized controlled studies (RCTs) yet. Therefore, to confirm these healthy effects and explore the underlying mechanism, new high quality RCTs with serum biomarker should be conducted.

Umbrella reviews rely on methodological quality and report transparency of the included meta-analyses [36]. Therefore, several limitations should be acknowledged in the current study. First, since umbrella reviews are observational studies, the reliability depended on the included meta-analysis directly and the original studies indirectly. It is impossible to control biases fielded in the original studies. Second, using the categorical association comparing the highest vs. lowest categories of coffee intake, or the linear association per one cup increase of coffee intake, may not reflect the real association (e.g. non-linear association) because we do not have the access to the individual-level data from the original studies. Finally, before a general recommendation on cancer prevention can be made, more researches are needed to improve understanding of how the volume and regularity of consumption, type of coffee, and style of preparation, such as adding milk or sugar, affect the risk of cancer.

## Conclusions

Even though coffee has been associated with a lower risk of several common cancers in the literature, the associations for only liver cancer and endometrial cancer were supported by highly suggestive evidence. Findings for cancer at other sites were less consistent, presenting hints of uncertainty and/or bias, which need more confirmative studies in the future.

## Additional Files

**Additional file 1:** Supplementary methods. **Table S1.** Excluded list through full-text review. **Table S2.** Description of meta-analyses of coffee consumption and cancer incidence with more than one meta-analysis. **Table S3.** AMSTAR score of included meta-analysis. **Table S4.** Description of meta-analyses only including cohort studies of coffee consumption and cancer incidence with more than one meta-analysis. **Table S5.** Description, evaluation of bias and heterogeneity in 34 associations of coffee intake and cancer incidence only including meta-analyses of cohort studies. **Table S6.** Summary of evidence grading for meta-analyses of cohort studies associating coffee intake and cancer incidence.

**Additional file 2:** **Figure S1**–**S36.** Forest plots of all observational studies. **Figure S37**–**S70**. Forest plots of cohort studies.

## Data Availability

No additional data are available.

## Abbreviations

CI: Confidence interval
IARC: International agency for research on cancer
PI: Predictive interval
WCRF: World cancer research fund
