# Supplementary material for: Coffee drinking and cancer risk: an umbrella review of meta-analyses of observational studies
Source: BMC Cancer. 2020 Feb 5;20:101. doi: 10.1186/s12885-020-6561-9 (PMC7003434; doi:10.1186/s12885-020-6561-9)
Supplement: Supplementary file 1 — Additional file 1: Supplementary methods. Table S1. Excluded list through full-text review. Table S2. Description of meta-analyses of coffee consumption and cancer incidence with more than one meta-analysis. Table S3. AMSTAR score of included meta-analysis. Table S4. Description of meta-analyses only including cohort studies of coffee consumption and cancer incidence with more than one meta-analysis. Table S5. Description, evaluation of bias and heterogeneity in 34 associations of coffee intake and cancer incidence only including meta-analyses of cohort studies. Table S6. Summary of evidence grading for meta-analyses of cohort studies associating coffee intake and cancer incidence. [file 12885_2020_6561_MOESM1_ESM.docx]

**Coffee drinking and cancer risk: an umbrella review of meta-analyses of observational studies**

Long-Gang Zhao, Zhuo-Ying Li, Guo-Shan Feng, Xiao-Wei Ji, Hong-Lan Li, Marc J. Gunter, Yong-Bing Xiang

**Additional Files Lists**

Supplementary methods

ST1 Excluded list through full text review

ST2 Description of meta-analyses of coffee consumption and risk of cancer with more than one meta-analysis

ST3 AMSTAR score of included meta-analysis

ST4 Description of meta-analyses (cohort studies) of coffee consumption and cancer incidence with more than one meta-analysis

ST5 Description, evaluation of bias and heterogeneity in 34 associations of coffee intake and cancer incidence (cohort studies)

ST6 Summary of evidence grading for meta-analyses of cohort studies associating coffee intake and cancer incidence

SF1-36 Forest plots of all observational studies
SF37-70 Forest plots of cohort studies

**Supplementary Methods**

**Literature search**

Three researchers (LGZ, ZYL, and GSF) independently searched the PubMed, Embase, Web of Science and the Cochrane database of systematic reviews from inception to February 2019 for meta-analyses or systematic reviews of observational studies investigating the association between coffee consumption and risk of any developing cancer. We did not apply any year, language, or publication status restrictions in the selection of eligible studies.

The search was based on the following terms: (coffee) AND (cancer OR cancers OR tumor OR tumors OR carcinoma OR carcinomas OR sarcoma OR neoplasm) AND (meta-analysis OR meta-analyses OR systematic review OR systematic reviews). Moreover, we manually searched the reference lists of included studies to identify additional articles that might be missed in the review of primary literature.

**Screening and selection procedure**

Firstly, three reviewers screened the titles and abstracts of all articles independently. Secondly, the potential included articles were downloaded the full text, examined in detail and screened for applicability through full text.

We included systematic reviews and meta-analyses of observational studies in humans on associations between coffee consumption and the risk of developing cancers. We included studies with comparisons of coffee intake, including the highest versus lowest and any linear dose-response. Randomized controlled trials were unavailable for our research question. We also excluded meta-analyses or systematic reviews that did not present study specific data (such as relative risks, 95% confidence intervals, et al.).

When we found more than one meta-analysis on the same association between exposure and outcome, we selected the study with the largest number of original studies. We defined one original study indicated one single cohort study. For example, if one paper reported the results for three cohorts, we considered it as three original studies. If the study lacked essential information, we selected an alternative one with enough information for further analysis.

**Data collection process**

For eligible systematic review and meta-analysis, two reviewers (ZYL and GSF) carried out the data extraction, and quality assessment independently. Disagreements were resolved by discussion with a third reviewer (LGZ). We developed a standard data extraction table to obtain the following information: first author’s last name, year of publication, exposure, unit of comparison, outcomes examined, number of included studies, number of cases, reported summary meta-analysis estimates (including heterogeneity measures) and bias assessment.

From each individual study in a meta-analysis, we extracted the first author’s last name, year of publication, epidemiological design, number of cases, maximally adjusted relative risk (odds ratio in case-control studies and hazard ratio or standardized incidence or mortality ratio in cohort studies) and their 95% confidence intervals.

**Assessment of methodological quality**

The methodological quality and bias of reviews were appraised by two reviewers using the revised Assessment of Multiple Systematic Reviews (AMSTAR 2) tool [1] based on the identification of critical domains.

**Data analysis**

*Estimation of summary effects*

We carried out a descriptive analysis of systematic reviews. The data from each systematic review and findings based on methodological quality were used to build evidence tables. Data from primary observational studies were used to perform meta-analyses. We reestimated the summary effect size and its 95% confidence interval [2]. When linear dose-response analyses presented results for more than one cup a day, we converted it to one cup. In the sensitivity analysis, we reselected meta-analysis with the largest number of only cohort studies from the database, which is considered less likely to be affected by recall bias.

*Assessment of heterogeneity*

We evaluated heterogeneity by estimating the variance between studies using Cochran’s Q test and the I-squared (I^2^) statistic [3, 4]. We also estimated the 95% prediction interval (95%PI), which further accounted for between-study heterogeneity and evaluates the uncertainty for the effect that would be expected in a new observational study addressing that same association [5].

*Evaluation of small study effects and excess significance*

An indication of small study effects was evaluated based on the Egger’s regression asymmetry test (*P* ≤ 0.10) [6]. We assessed excess significance bias by evaluating whether the observed number of studies with nominally statistically significant results (“positive” studies, *P* ≤ 0.05) in the published literature was different from the expected number of studies with statistically significant results [7]. The actual size of the true effect in each meta-analysis was assumed to be the effect of the largest study in each meta-analysis, which is defined based on the smallest standard error. Sensitivity analysis was performed using the summary fixed and random effects estimates as alternative plausible effect sizes. Excess significance for single meta-analyses was claimed at *P* ≤ 0.10.

**Reference**

1. Shea BJ, Reeves BC, Wells G, Thuku M, Hamel C, Moran J, Moher D, Tugwell P, Welch V, Kristjansson E *et al*: AMSTAR 2: a critical appraisal tool for systematic reviews that include randomised or non-randomised studies of healthcare interventions, or both. BMJ 2017, 358:j4008.

2. DerSimonian R, Laird N: Meta-analysis in clinical trials. Control Clin Trials 1986, 7(3):177-188.

3. Ioannidis JP, Patsopoulos NA, Evangelou E: Uncertainty in heterogeneity estimates in meta-analyses. BMJ 2007, 335(7626):914-916.

4. Higgins JP, Thompson SG: Quantifying heterogeneity in a meta-analysis. Stat Med 2002, 21(11):1539-1558.

5. Riley RD, Higgins JP, Deeks JJ: Interpretation of random effects meta-analyses. BMJ 2011, 342:d549.

6. Egger M, Davey Smith G, Schneider M, Minder C: Bias in meta-analysis detected by a simple, graphical test. BMJ 1997, 315(7109):629-634.

7. Ioannidis JPA: Clarifications on the application and interpretation of the test for excess significance and its extensions. J Math Psychol 2013, 57(5):184-187.

| **ST1 Excluded list through full text review** | | |
| --- | --- | --- |
| **No.** | **Reference** | **Excluded reason** |
| 1 | Malmir H, Esmaillzadeh A. The relationship between tea and coffee consumption and glioma: A systematic review. Journal of Babol University of Medical Sciences 2017;19:69-75. | Coffee and tea are combined |
| 2 | Bamia C, Turati F, Guercio V, Guha N, Loomis D, Tavani A. Coffee intake and risk of breast and ovarian cancer: Updated systematic review and meta-analysis. Ann Nutr Metab 2017;71:950-1. | Conference Abstract |
| 3 | Huang SJ, Xu H, Wei W. Coffee consumption and risk of endometrial cancer: A systematic review and Meta-analysis of prospective studies. Chinese Journal of Cancer Prevention and Treatment 2013;20:1525-30. | No single study RR included |
| 4 | Alzalabani A, Wesselius A, Stewart KFJ, Zeegers MP. Modifiable risk factors of bladder cancer: A quantitative review of meta-analyses. Eur J Epidemiol 2015;30:888. | No single study RR included |
| 5 | Dhote R, Pellicer-Coeuret M, Thiounn N, Debre B, Vidal-Trecan G. Risk factors for adult renal cell carcinoma: a systematic review and implications for prevention. BJU Int 2000;86:20-7. | No single study RR included |
| 6 | Hong X, Xu Q, Lan K, Huang H, Zhang Y, Chen S, et al. The effect of daily fluid management and beverages consumption on the risk of bladder cancer: A meta-analysis of observational study. Nutr Cancer 2018. | Pooled RR missing, only design subgroup |
| 7 | Horisaki K, Takahashi K, Ito H, Matsui S. A dose-response meta-analysis of coffee consumption and colorectal cancer risk in the Japanese population: Application of a cubic-spline model. J Epidemiol 2018;28:503-9. | Pooled RR missing, only dose-response analysis |
| 8 | Yang TO, Crowe F, Cairns BJ, Reeves GK, Beral V. Tea and coffee and risk of endometrial cancer: Cohort study and meta-analysis. Am J Clin Nutr 2015;101:570-8. | Pooled RR missing, only dose-response analysis |
| 9 | Tian C, Wang W, Hong Z, Zhang X. Coffee consumption and risk of colorectal cancer: A dose-response analysis of observational studies. Cancer Causes Control 2013;24:1265-8. | Pooled RR missing, only subgroup of coffee |
| 10 | Wang Y, Yu X, Wu Y, Zhang D. Coffee and tea consumption and risk of lung cancer: A dose-response analysis of observational studies. Lung Cancer 2012;78:169-70. | Pooled RR missing, only subgroup of coffee |
| 11 | Caini S, Cattaruzza S, Bendinelli B, Tosti G, Masala G, Gnagnarella P, et al. Coffee, tea and caffeine intake and the risk of non-melanoma skin cancer: a review of the literature and meta-analysis. Eur J Nutr 2017;56. | Only data from Japan |
| 12 | Liu J, Shen B, Shi M, Cai J. Higher caffeinated coffee intake is associated with reduced malignant melanoma risk: A meta-analysis Study. PLoS One 2016;11. | Duplicated with Huang, 2013, CEBM |
| 13 | Alicandro G, Tavani A, La Vecchia C. Coffee and cancer risk: A summary overview. Eur J Cancer Prev 2017;26:424-32. | Review only |
| 14 | Arab L. Epidemiologic evidence on coffee and cancer. Nutr Cancer 2010;62:271-83. | Review only |
| 15 | Dagnelie PC, Schuurman AG, Goldbohm RA, Van den Brandt PA. Diet, anthropometric measures and prostate cancer risk: a review of prospective cohort and intervention studies. BJU Int 2004;93:1139-50. | Review only |
| 16 | Einecke D. New indications from a meta analysis: Does coffee protect against prostate cancer? MMW-Fortschritte der Medizin 2014;156:1. | Review only |
| 17 | Ekbom A. Review: substantial coffee consumption was associated with a lower risk of colorectal cancer in the general population. Gut 1999;44:597. | Review only |
| 18 | Marques-Vidal P, Ravasco P, Camilo ME. Foodstuffs and colorectal cancer risk: A review. Clin Nutr 2006;25:14-36. | Review only |
| 19 | Masko EM, Allott EH, Freedland SJ. The relationship between nutrition and prostate cancer: Is more always better? Eur Urol 2013;63:810-20. | Review only |
| 20 | Merritt MA, Gunter MJ. Coffee drinking and endometrial cancer. Current Nutrition Reports 2015;4:40-6. | Review only |
| 21 | Mullie P, Autier P, Boyle P. Overview of meta-analyses and pooled analyses of nutrition and breast cancer risk. J Clin Oncol 2013;31. | Review only |
| 22 | Pelucchi C, La Vecchia C. Alcohol, coffee, and bladder cancer risk: a review of epidemiological studies. Eur J Cancer Prev 2009;18:62-8. | Review only |
| 23 | Pourshahidi LK, Navarini L, Petracco M, Strain JJ. A comprehensive overview of the risks and benefits of coffee consumption. Comprehensive Reviews in Food Science and Food Safety 2016;15:671-84. | Review only |
| 24 | Tavani A, Vecchia CL. Coffee, decaffeinated coffee, tea and cancer of the colon and rectum: A review of epidemiological studies, 1990-2003. Cancer Causes Control 2004;15:743-57. | Review only |
| 25 | Zeegers MPA, Kellen E, Buntinx F, van den Brandt PA. The association between smoking, beverage consumption, diet and bladder cancer: a systematic literature review. World J Urol 2004;21:392-401. | Review only |
| 26 | Discacciati A, Orsini N, Wolk A. Coffee consumption and risk of nonaggressive, aggressive and fatal prostate cancer--a dose-response meta-analysis. Ann Oncol 2014;25:584-91. | Subgroup of cancer |
| 27 | Rosenberg L. Coffee and tea consumption in relation to the risk of large bowel cancer: a review of epidemiologic studies. Cancer Lett 1990;52(3):163-71. | No single study RR included |
| 28 | Akter S, Kashino I, Mizoue T, Matsuo K, Ito H, Wakai K, et al. Coffee drinking and colorectal cancer risk: An evaluation based on a systematic review and meta-analysis among the Japanese population. Jpn J Clin Oncol 2016;46:781-7. | More than one meta-analysis on associations |
| 29 | Bai K, Cai Q, Jiang Y, Lv L. Coffee consumption and risk of hepatocellular carcinoma: A meta-analysis of eleven epidemiological studies. Onco Targets Ther 2016;9:4369-75. | More than one meta-analysis on associations |
| 30 | Berretta M, Micek A, Lafranconi A, Rossetti S, Di Francia R, De Paoli P, et al. Coffee consumption is not associated with ovarian cancer risk: A dose-response meta-analysis of prospective cohort studies. Oncotarget 2018;9:20807-15. | More than one meta-analysis on associations |
| 31 | Braem MGM, Onland-Moret NC, Schouten LJ, Tjønneland A, Hansen L, Dahm CC, et al. Coffee and tea consumption and the risk of ovarian cancer: A prospective cohort study and updated meta-analysis. Am J Clin Nutr 2012;95:1172-81. | More than one meta-analysis on associations |
| 32 | Bravi F, Bosetti C, Tavani A, Bagnardi V, Gallus S, Negri E, et al. Coffee drinking and hepatocellular carcinoma risk: A meta-analysis. Hepatology 2007;46:430-5. | More than one meta-analysis on associations |
| 33 | Bravi F, Bosetti C, Tavani A, Gallus S, La Vecchia C. Coffee reduces risk for hepatocellular carcinoma: An updated meta-analysis. Clin Gastroenterol Hepatol 2013;11:1413-21. | More than one meta-analysis on associations |
| 34 | Bravi F, Scotti L, Bosetti C, Gallus S, Negri E, La Vecchia C, et al. Coffee drinking and endometrial cancer risk: a metaanalysis of observational studies. Am J Obstet Gynecol 2009;200:130-5. | More than one meta-analysis on associations |
| 35 | Bravi F, Tavani A, Bosetti C, Boffetta P, La Vecchia C. Coffee and the risk of hepatocellular carcinoma and chronic liver disease: A systematic review and meta-analysis of prospective studies. Eur J Cancer Prev 2017;26:368-77. | More than one meta-analysis on associations |
| 36 | Cao S, Liu L, Yin X, Wang Y, Liu J, Lu Z. Coffee consumption and risk of prostate cancer: A meta-analysis of prospective cohort studies. Carcinogenesis 2014;35:256-61. | More than one meta-analysis on associations |
| 37 | Chen J, Long S. Tea and coffee consumption and risk of laryngeal cancer: A systematic review meta-analysis. PLoS One 2014;9. | More than one meta-analysis on associations |
| 38 | Cheng J, Su H, Zhu R, Wang X, Peng M, Song J, et al. Maternal coffee consumption during pregnancy and risk of childhood acute leukemia: a metaanalysis. Am J Obstet Gynecol 2014;210:151.e1-.e10. | More than one meta-analysis on associations |
| 39 | Deng W, Yang H, Wang J, Cai J, Bai Z, Song J, et al. Coffee consumption and the risk of incident gastric cancer--A meta-analysis of prospective cohort studies. Nutr Cancer 2016;68:40-7. | More than one meta-analysis on associations |
| 40 | Dong J, Zou J, Yu XF. Coffee drinking and pancreatic cancer risk: A meta-analysis of cohort studies. World J Gastroenterol 2011;17:1204-10. | More than one meta-analysis on associations |
| 41 | Fang X, Wei J, He X, An P, Wang H, Jiang L, et al. Landscape of dietary factors associated with risk of gastric cancer: A systematic review and dose-response meta-analysis of prospective cohort studies. Eur J Cancer 2015;51:2820-32. | More than one meta-analysis on associations |
| 42 | Galeone C, Turati F, La Vecchia C, Tavani A. Coffee consumption and risk of colorectal cancer: A meta-analysis of case-control studies. Cancer Causes Control 2010;21:1949-59. | More than one meta-analysis on associations |
| 43 | Giovannucci E. Meta-analysis of coffee consumption and risk of colorectal cancer. Am J Epidemiol 1998;147:1043-52. | More than one meta-analysis on associations |
| 44 | Huang TB, Guo ZF, Zhang XL, Zhang XP, Liu H, Geng J, et al. Coffee consumption and urologic cancer risk: A meta-analysis of cohort studies. Int Urol Nephrol 2014;46:1481-93. | More than one meta-analysis on associations |
| 45 | Je Y, Giovannucci E. Coffee consumption and risk of endometrial cancer: Findings from a large up-to-date meta-analysis. Int J Cancer 2012;131:1700-10. | More than one meta-analysis on associations |
| 46 | Je Y, Liu W, Giovannucci E. Coffee consumption and risk of colorectal cancer: A systematic review and meta-analysis of prospective cohort studies. Int J Cancer 2009;124:1662-8. | More than one meta-analysis on associations |
| 47 | Lafranconi A, Micek A, Galvano F, Rossetti S, Del Pup L, Berretta M, et al. Coffee decreases the risk of endometrial cancer: A dose–response meta-analysis of prospective cohort studies. Nutrients 2017;9. | More than one meta-analysis on associations |
| 48 | Lafranconi A, Micek A, Paoli PD, Bimonte S, Rossi P, Quagliariello V, et al. Coffee intake decreases risk of postmenopausal breast cancer: A dose-response meta-analysis on prospective cohort studies. Nutrients 2018;10. | More than one meta-analysis on associations |
| 49 | Larsson SC, Wolk A. Coffee Consumption and Risk of Liver Cancer: A Meta-Analysis. Gastroenterology 2007;132:1740-5. | More than one meta-analysis on associations |
| 50 | Li L, Gan Y, Wu C, Qu X, Sun G, Lu Z. Coffee consumption and the risk of gastric cancer: A meta-analysis of prospective cohort studies. BMC Cancer 2015;15. | More than one meta-analysis on associations |
| 51 | Liu H, Hu GH, Wang XC, Huang TB, Xu L, Lai P, et al. Coffee consumption and prostate cancer risk: A meta-analysis of cohort studies. Nutr Cancer 2015;67:392-400. | More than one meta-analysis on associations |
| 52 | Liu H, Hua Y, Zheng X, Shen Z, Luo H, Tao X, et al. Effect of coffee consumption on the risk of gastric cancer: A systematic review and meta-analysis of prospective cohort studies. PLoS One 2015;10. | More than one meta-analysis on associations |
| 53 | Lu Y, Zhai L, Zeng J, Peng Q, Wang J, Deng Y, et al. Coffee consumption and prostate cancer risk: An updated meta-analysis. Cancer Causes Control 2014;25:591-604. | More than one meta-analysis on associations |
| 54 | Micek A, Godos J, Lafranconi A, Marranzano M, Pajak A. Caffeinated and decaffeinated coffee consumption and melanoma risk: a dose-response meta-analysis of prospective cohort studies. Int J Food Sci Nutr 2018;69:417-26. | More than one meta-analysis on associations |
| 55 | Milne E, Royle JA, Bennett LC, de Klerk NH, Bailey HD, Bower C, et al. Maternal consumption of coffee and tea during pregnancy and risk of childhood ALL: results from an Australian case-control study. Cancer Causes Control 2011;22:207-18. | More than one meta-analysis on associations |
| 56 | Nie K, Xing Z, Huang W, Wang W, Liu W. Coffee intake and risk of pancreatic cancer: an updated meta-analysis of prospective studies. Minerva Med 2016;107:270-8. | More than one meta-analysis on associations |
| 57 | Nishi M, Ohba S, Hirata K, Miyake H. Dose-response relationship between coffee and the risk of pancreas cancer. Jpn J Clin Oncol 1996;26:42-8. | More than one meta-analysis on associations |
| 58 | Park CH, Myung SK, Kim TY, Seo HG, Jeon YJ, Kim Y. Coffee consumption and risk of prostate cancer: A meta-analysis of epidemiological studies. BJU Int 2010;106:762-9. | More than one meta-analysis on associations |
| 59 | Ran HQ, Wang JZ, Sun CQ. Coffee consumption and pancreatic cancer risk: An update meta-analysis of cohort studies. Pakistan Journal of Medical Sciences 2016;32:253-9. | More than one meta-analysis on associations |
| 60 | Shen Z, liu H, Cao H. Coffee consumption and risk of gastric cancer: An updated meta-analysis. Clin Res Hepatol Gastroenterol 2015;39:245-53. | More than one meta-analysis on associations |
| 61 | Tang N, Zhou B, Wang B, Yu R. Coffee consumption and risk of breast cancer: a metaanalysis. Am J Obstet Gynecol 2009;200:290.e1-9. | More than one meta-analysis on associations |
| 62 | Turati F, Galeone C, La Vecchia C, Garavello W, Tavani A. Coffee and cancers of the upper digestive and respiratory tracts: Meta-analyses of observational studies. Ann Oncol 2011;22:536-44. | More than one meta-analysis on associations |
| 63 | Vieira AR, Abar L, Chan DSM, Vingeliene S, Polemiti E, Stevens C, et al. Foods and beverages and colorectal cancer risk: A systematic review and meta-analysis of cohort studies, an update of the evidence of the WCRF-AICR Continuous Update Project. Ann Oncol 2017;28:1788-802. | More than one meta-analysis on associations |
| 64 | Wang AQ, Wang SS, Zhu CP, Huang HC, Wu LC, Wan XS, et al. Coffee and cancer risk: A meta-analysis of prospective observational studies. Sci Rep 2016;6. | More than one meta-analysis on associations |
| 65 | Wang J, Li XT, Zhang DF. Coffee consumption and the risk of cutaneous melanoma: a meta-analysis. Eur J Nutr 2016;55:1317-29. | More than one meta-analysis on associations |
| 66 | Xie Y, Huang S, He T, Su Y. Coffee consumption and risk of gastric cancer: an updated meta-analysis. Asia Pac J Clin Nutr 2016;25:578-88. | More than one meta-analysis on associations |
| 67 | Xie Y, Qin J, Nan G, Huang S, Wang Z, Su Y. Coffee consumption and the risk of lung cancer: An updated meta-analysis of epidemiological studies. Eur J Clin Nutr 2016;70:199-206. | More than one meta-analysis on associations |
| 68 | Yan KK, Xu XJ, Liu XD, Wang XK, Hua SC, Wang CP, et al. The Associations Between Maternal Factors During Pregnancy and the Risk of Childhood Acute Lymphoblastic Leukemia: A Meta-Analysis (vol 62, pg 1162, 2015). Pediatr Blood Cancer 2016;63:953-4. | More than one meta-analysis on associations |
| 69 | Yu C, Cao Q, Chen P, Yang S, Deng M, Wang Y, et al. An updated dose-response meta-analysis of coffee consumption and liver cancer risk. Sci Rep 2016;6:37488. | More than one meta-analysis on associations |
| 70 | Yu X, Bao Z, Zou J, Dong J. Coffee consumption and risk of cancers: A meta-analysis of cohort studies. BMC Cancer 2011;11. | More than one meta-analysis on associations |
| 71 | Zeegers MPA, Tan FES, Goldbohm RA, Van Den Brandt PA. Are coffee and tea consumption associated with urinary tract cancer risk? A systematic review and meta-analysis. Int J Epidemiol 2001;30:353-62. | More than one meta-analysis on associations |
| 72 | Zeng SB, Weng H, Zhou M, Duan XL, Shen XF, Zeng XT. Long-term coffee consumption and risk of gastric cancer: A PRISMA-compliant dose-response meta-analysis of prospective cohort studies. Medicine (United States) 2015;94. | More than one meta-analysis on associations |
| 73 | Zhang J, Zhou B, Hao CZ. Coffee consumption and risk of esophageal cancer incidence: A meta-analysis of epidemiologic studies. Medicine 2018;97. | More than one meta-analysis on associations |
| 74 | Zhang Y, Wang X, Cui D. Association between coffee consumption and the risk of oral cancer: A meta-analysis of observational studies. Int J Clin Exp Med 2015;8:11657-65. | More than one meta-analysis on associations |
| 75 | Zhong S, Chen W, Yu X, Chen Z, Hu Q, Zhao J. Coffee consumption and risk of prostate cancer: An up-to-date meta-analysis. Eur J Clin Nutr 2014;68:330-7. | More than one meta-analysis on associations |
| 76 | Zhou Q, Luo ML, Li H, Li M, Zhou JG. Coffee consumption and risk of endometrial cancer: a dose-response meta-analysis of prospective cohort studies. Sci Rep 2015;5:13410. | More than one meta-analysis on associations |
| 77 | Zhou Y, Tian C, Jia C. A dose-response meta-analysis of coffee consumption and bladder cancer. Prev Med 2012;55:14-22. | More than one meta-analysis on associations |
| 78 | Li TD, Yang HW, Wang P, Song CH, Wang KJ, Dai LP, et al. Coffee consumption and risk of pancreatic cancer: a systematic review and dose-response meta-analysis. Int J Food Sci Nutr 2019:1-11. | More than one meta-analysis on associations |

| **ST2 Description of meta-analyses of coffee consumption and cancer incidence with more than one meta-analysis** | | | | | | | | | | |  |  |  |  |  |  |
| --- | --- | --- | --- | --- | --- | --- | --- | --- | --- | --- | --- | --- | --- | --- | --- | --- |
| Author | Year | Journal | Cancer type | Unit of comparison | Co | CC | CS | Total | RR | LL | UL | Model | I^2^ | P for Q | P for Egger | Included |
| Thomopoulos | 2015 | Cancer Epidemiol | AL | H/L | 0 | 6 | 0 | 6 | 1.57 | 1.16 | 2.11 | R | 55.2 | 0.048 | NR | Yes |
| Cheng | 2014 | Am J Obstet Gynecol | AL | H/L | 0 | 4 | 0 | 4 | 1.72 | 1.37 | 2.16 | F | 35 | 0.202 | 0.406 | No |
| Yu | 2011 | BMC Cancer | AL | H/L | 2 | 0 | 0 | 2 | 0.64 | 0.51 | 0.77 | F | 30.4 | 0.231 | NR | No |
| Thomopoulos | 2015 | Cancer Epidemiol | ALL | H/L | 0 | 7 | 0 | 7 | 1.43 | 1.22 | 1.68 | R | 0 | 0.464 | NR | Yes |
| Cheng | 2014 | Am J Obstet Gynecol | ALL | H/L | 0 | 5 | 0 | 5 | 1.65 | 1.28 | 2.12 | F | 47 | 0.11 | 0.596 | No |
| Milne | 2011 | Cancer Causes Control | ALL | H/L | 0 | 3 | 0 | 3 | 1.67 | 1.2 | 2.33 | F | 42.7 | 0.174 | NR | No |
| Yan | 2015 | Pediatric Blood and Cancer | ALL | Y/N | 0 | 3 | 0 | 3 | 1.44 | 1.07 | 1.92 | R | 41.8 | NR | NR | No |
| Yu | 2011 | BMC Cancer | all cancers | H/L | 40 | 0 | 0 | 40 | 0.82 | 0.74 | 0.89 | R | 67.7 | <0.001 | 0.793 | No |
| Yu | 2011 | BMC Cancer | all cancers | per 1 cup | 40 | 0 | 0 | 40 | 0.97 | 0.96 | 0.98 | NR | NR | NR | NR | No |
| Thomopoulos | 2015 | Cancer Epidemiol | AML | H/L | 0 | 6 | 0 | 6 | 1.81 | 0.93 | 3.53 | R | 53.7 | 0.056 | NR | Yes |
| Cheng | 2014 | Am J Obstet Gynecol | AML | H/L | 0 | 4 | 0 | 4 | 1.58 | 1.2 | 2.08 | F | 0 | 0.643 | 0.018 | No |
| Godos | 2017 | Nutrients | Biliary tract | H/L | 3 | 2 | 0 | 5 | 0.83 | 0.64 | 1.08 | R | 0 | 0.58 | >0.05 | Yes |
| Wu | 2015 | Sci Rep | bladder | H/L | 5 | 25 | 0 | 30 | 1.33 | 1.19 | 1.48 | R | 38.4 | 0.008 | 0.051 | Yes |
| Zhou | 2012 | Prev Med | bladder | H/L | 0 | 20 | 0 | 20 | 1.12 | 0.93 | 1.34 | R | 46.1 | 0.054 | No | No |
| Wang | 2016 | Sci Rep | bladder | H/L | 10 | 0 | 0 | 10 | 1.12 | 0.94 | 1.34 | F | 39.6 | 0.094 | 0.01 | No |
| Huang | 2014 | Int Urol Nephrol | bladder | H/L | 5 | 0 | 0 | 5 | 1.08 | 0.71 | 1.66 | R | 62.9 | 0.009 | NR | No |
| Zhou | 2012 | Prev Med | bladder | H/L | 5 | 0 | 0 | 5 | 1.45 | 1.29 | 1.63 | R | 31.8 | 0.086 | No | No |
| Yu | 2011 | BMC Cancer | bladder | H/L | 5 | 0 | 0 | 5 | 0.83 | 0.73 | 0.94 | F | 39.3 | 0.106 | NR | No |
| Wu | 2015 | Sci Rep | bladder | per 1 cup | 5 | 21 | 0 | 26 | 1.05 | 1.03 | 1.06 | R | NR | NR | NR | No |
| Yu | 2011 | BMC Cancer | bladder | per 1 cup | 5 | 0 | 0 | 5 | 1.03 | 0.99 | 1.06 | NR | NR | NR | NR | No |
| Zhou | 2012 | Prev Med | bladder | per 2 cups | 0 | 17 | 0 | 17 | 1.1 | 0.87 | 1.17 | NR | NR | NR | NR | No |
| Huang | 2014 | Int Urol Nephrol | bladder | per 2 cups | 5 | 0 | 0 | 5 | 1.03 | 0.8 | 1.3 | NR | NR | NR | NR | No |
| Zhou | 2012 | Prev Med | bladder | per 2 cups | 4 | 0 | 0 | 4 | 1.1 | 1.07 | 1.15 | NR | NR | NR | NR | No |
| Lafranconi | 2018 | Nutrients | breast | H/L | 16 | 0 | 0 | 16 | 0.96 | 0.93 | 1 | R | 7 | 0.37 | >0.05 | No |
| Jiang | 2013 | Gynecol Oncol | breast | H/L | 17 | 17 | 0 | 34 | 0.97 | 0.93 | 1 | F | 14.2 | 0.229 | 0.23 | Yes |
| Li | 2013 | PLoS One | breast | H/L | 16 | 10 | 0 | 26 | 0.96 | 0.93 | 1 | R | 0 | 0.769 | 0.54 | No |
| Tang | 2009 | Am J Obstet Gynecol | breast | H/L | 9 | 9 | 0 | 18 | 0.95 | 0.9 | 1 | R | 0 | 0.677 | 0.874 | No |
| Wang | 2016 | Sci Rep | breast | H/L | 17 | 0 | 0 | 17 | 0.99 | 0.94 | 1.04 | F | 0 | 0.55 | 0.75 | No |
| Yu | 2011 | BMC Cancer | breast | H/L | 11 | 0 | 0 | 11 | 0.94 | 0.91 | 0.98 | F | 28.7 | 0.172 | NR | No |
| Yu | 2011 | BMC Cancer | breast | per 1 cup | 11 | 0 | 0 | 11 | 0.99 | 0.97 | 1 | NR | NR | NR | NR | No |
| Li | 2013 | PLoS One | breast | per 2 cups | 15 | 9 | 0 | 24 | 0.98 | 0.97 | 1 | R | 0 | 0.795 | NR | Yes |
| Jiang | 2013 | Gynecol Oncol | breast | per 2 cups | 12 | 8 | 0 | 20 | 0.98 | 0.96 | 1 | NR | NR | NR | NR | No |
| Tang | 2009 | Am J Obstet Gynecol | breast | per 2 cups | 8 | 7 | 0 | 15 | 0.98 | 0.96 | 1 | R | 0 | 0.599 | NR | No |
| Gan | 2017 | Oncotarget | colon | H/L | 16 | 0 | 0 | 16 | 0.92 | 0.83 | 1.02 | R | 29.9 | 0.124 | 0.699 | Yes |
| Galeone | 2010 | Cancer Causes Control | colon | H/L | 0 | 15 | 0 | 15 | 0.75 | 0.64 | 0.88 | R | 60.9 | NR | >0.05 | No |
| Wang | 2016 | Sci Rep | colon | H/L | 9 | 0 | 0 | 9 | 0.87 | 0.78 | 0.96 | NR | NR | NR | NR | No |
| Galeone | 2010 | Cancer Causes Control | colon | per 2 cups | 0 | 12 | 0 | 12 | 0.95 | 0.92 | 0.98 | R | 60.8 | 0.002 | >0.05 | No |
| Gan | 2017 | Oncotarget | colon | per 4 cups | 15 | 0 | 0 | 15 | 0.93 | 0.87 | 0.99 | R | 23 | 0.199 | 0.858 | Yes |
| Li | 2013 | Public Health Nutr | colorectal | H/L | 15 | 25 | 0 | 40 | 0.85 | 0.75 | 0.97 | R | 64 | <0.001 | 0.69 | Yes |
| Galeone | 2010 | Cancer Causes Control | colorectal | H/L | 0 | 24 | 0 | 24 | 0.7 | 0.6 | 0.81 | R | 50 | NR | >0.05 | No |
| Wang | 2016 | Sci Rep | colorectal | H/L | 21 | 0 | 0 | 21 | 0.96 | 0.91 | 1.02 | F | 23.6 | 0.16 | 0.82 | No |
| Gan | 2017 | Oncotarget | colorectal | H/L | 19 | 0 | 0 | 19 | 0.98 | 0.9 | 1.06 | R | 41.4 | 0.031 | 0.764 | No |
| Giovannucci | 1998 | Am J Epidemiol | colorectal | H/L | 5 | 12 | 0 | 17 | 0.76 | 0.66 | 0.89 | R | NR | NR | NR | No |
| Yu | 2011 | BMC Cancer | colorectal | H/L | 15 | 0 | 0 | 15 | 0.89 | 0.8 | 0.97 | R | 75.3 | <0.001 | NR | No |
| Je | 2009 | Int J Cancer | colorectal | H/L | 12 | 0 | 0 | 12 | 0.91 | 0.81 | 1.02 | R | 0 | 0.73 | 0.03 | No |
| Akter (JPN) | 2016 | Jpn J Clin Oncol | colorectal | H/L | 9 | 0 | 0 | 9 | 0.78 | 0.65 | 0.95 | R | 51.6 | 0.013 | 0.3 | No |
| Akter (JPN) | 2016 | Jpn J Clin Oncol | colorectal | H/L | 5 | 0 | 0 | 5 | 0.95 | 0.77 | 1.17 | R | 28.1 | 0.147 | 0.3 | No |
| Yu | 2011 | BMC Cancer | colorectal | per 1 cup | 15 | 0 | 0 | 15 | 0.99 | 0.97 | 1.01 | NR | NR | NR | NR | No |
| Vieira | 2017 | Ann Oncol | colorectal | per 1 cup | 14 | 0 | 0 | 14 | 1 | 0.99 | 1.02 | R | 44.2 | 0.05 | 0.002 | No |
| Galeone | 2010 | Cancer Causes Control | colorectal | per 1 cup | 0 | 12 | 0 | 12 | 0.94 | 0.91 | 0.98 | R | 69.3 | <0.001 | >0.05 | No |
| Gan | 2017 | Oncotarget | colorectal | per 4 cups | 17 | 0 | 0 | 17 | 0.93 | 0.88 | 0.99 | R | 34.3 | 0.082 | 0.434 | Yes |
| Lafranconi | 2017 | Nutrients | endometrial | H/L | 12 | 0 | 0 | 12 | 0.79 | 0.73 | 0.87 | R | 28 | 0.19 | >0.05 | No |
| Lukic | 2018 | Nutr Cancer | endometrial | H/L | 12 | 7 | 0 | 19 | 0.74 | 0.68 | 0.81 | R | 32 | 0.09 | <0.05 | Yes |
| Je | 2012 | Int J Cancer | endometrial | H/L | 6 | 10 | 0 | 16 | 0.71 | 0.62 | 0.81 | R | 29.1 | 0.132 | 0.33 | No |
| Zhou | 2015 | Sci Rep | endometrial | H/L | 14 | 0 | 0 | 14 | 0.8 | 0.74 | 0.86 | R | 31 | 0.13 | 0.03 | No |
| Wang | 2016 | Sci Rep | endometrial | H/L | 12 | 0 | 0 | 12 | 0.73 | 0.67 | 0.8 | F | 0 | 0.58 | 0.16 | No |
| Huang | 2013 | Chin J Evid-based Med | endometrial | H/L | 10 | 0 | 0 | 10 | 0.69 | 0.62 | 0.78 | F | 0 | 0.492 | Begg=0.592 | No |
| Bravi | 2009 | Am J Obstet Gynecol | endometrial | H/L | 2 | 7 | 0 | 9 | 0.64 | 0.48 | 0.86 | R | NR | 0.016 | 0.6 | No |
| Yu | 2011 | BMC Cancer | endometrial | H/L | 4 | 0 | 0 | 4 | 0.74 | 0.63 | 0.84 | F | 13.5 | 0.325 | NR | No |
| Je | 2012 | Int J Cancer | endometrial | per 1 cup | 6 | 8 | 0 | 14 | 0.92 | 0.9 | 0.95 | R | 25.6 | 0.18 | 0.07 | No |
| Zhou | 2015 | Sci Rep | endometrial | per 1 cup | 11 | 0 | 0 | 11 | 0.95 | 0.93 | 0.97 | R | NR | NR | NR | No |
| Yu | 2011 | BMC Cancer | endometrial | per 1 cup | 4 | 0 | 0 | 4 | 0.93 | 0.89 | 0.98 | NR | NR | NR | NR | No |
| Wang | 2016 | Sci Rep | endometrial | per 2 cups | 11 | 0 | 0 | 11 | 0.88 | 0.85 | 0.92 | NR | NR | NR | NR | No |
| Huang | 2013 | Chin J Evid-based Med | endometrial | per 2 cups | 7 | 0 | 0 | 7 | 0.88 | 0.83 | 0.92 | F | 35.6 | 0.156 | NR | Yes |
| Zhang | 2018 | Medicine (Baltimore) | esophageal | H/L | 2 | 9 | 0 | 11 | 0.91 | 0.73 | 1.04 | R | 52.6 | 0.021 | >0.1 | No |
| Wang | 2016 | Sci Rep | esophageal | H/L | 6 | 0 | 0 | 6 | 0.86 | 0.71 | 1.04 | F | 0 | 0.64 | 0.69 | No |
| Yu | 2011 | BMC Cancer | esophageal | H/L | 2 | 0 | 0 | 2 | 0.55 | 0.37 | 0.74 | F | 0 | 0.478 | NR | No |
| Zheng | 2013 | Nutr Cancer | esophageal | H/L | 4 | 10 | 0 | 14 | 0.88 | 0.76 | 1.01 | R | 38.4 | 0.055 | 0.53 | Yes |
| Turati | 2011 | Ann Oncol | esophageal (EAC) | H/L | 0 | 3 | 0 | 3 | 1.18 | 0.81 | 1.71 | R | 43.7 | 0.169 | >0.05 | No |
| Turati | 2011 | Ann Oncol | esophageal (ESCC) | H/L | 1 | 6 | 0 | 7 | 0.87 | 0.65 | 1.17 | R | 74.6 | 0.001 | >0.05 | No |
| Botelho | 2006 | Cad Saude Publica | gastric | H/L | 7 | 16 | 0 | 23 | 0.97 | 0.86 | 1.09 | R | NR | 0.08 | 0.92 | Yes |
| Xie | 2016 | Asia Pac J Clin Nutr | gastric | H/L | 9 | 13 | 0 | 22 | 0.94 | 0.8 | 1.1 | R | 59.4 | <0.001 | 0.422 | No |
| Deng | 2016 | Nutr Cancer | gastric | H/L | 13 | 0 | 0 | 13 | 1.16 | 1.03 | 1.32 | F | 26.6 | 0.162 | 0.29 | No |
| Li | 2015 | BMC Cancer | gastric | H/L | 13 | 0 | 0 | 13 | 1.13 | 0.94 | 1.35 | R | 38 | 0.044 | 0.173 | No |
| Wang | 2016 | Sci Rep | gastric | H/L | 12 | 0 | 0 | 12 | 1.15 | 0.96 | 1.37 | R | 49.2 | 0.027 | 0.85 | No |
| Xie | 2014 | Nutrients | gastric | H/L | 12 | 0 | 0 | 12 | 1.12 | 0.93 | 1.36 | R | 37 | 0.074 | 0.03 | No |
| Zeng | 2015 | Medicine (Baltimore) | gastric | H/L | 9 | 0 | 0 | 9 | 1.18 | 0.9 | 1.55 | R | 50.9 | 0.01 | 0.65 | No |
| Liu | 2015 | PLoS One | gastric | H/L | 9 | 0 | 0 | 9 | 1.05 | 0.88 | 1.25 | R | 74 | <0.001 | 0.602 | No |
| Shen | 2015 | Clin Res Hepatol Gastroenterol | gastric | H/L | 8 | 0 | 0 | 8 | 1.24 | 1.03 | 1.49 | F | 25.4 | 0.202 | 0.6 | No |
| Fang | 2015 | Eur J Cancer | gastric | H/L | 8 | 0 | 0 | 8 | 1.02 | 0.79 | 1.31 | R | 57.6 | NR | >0.1 | No |
| Yu | 2011 | BMC Cancer | gastric | H/L | 8 | 0 | 0 | 8 | 1 | 0.77 | 1.24 | R | 66.6 | 0.004 | NR | No |
| Yu | 2011 | BMC Cancer | gastric | per 1 cup | 8 | 0 | 0 | 8 | 1.13 | 1.03 | 1.24 | NR | NR | NR | NR | No |
| Liu | 2015 | PLoS One | gastric | per 1 cup | 5 | 0 | 0 | 5 | 1.01 | 0.98 | 1.05 | F | NR | 0.102 | NR | No |
| Xie | 2014 | Nutrients | gastric | per 2 cups | 9 | 0 | 0 | 9 | 1.03 | 0.95 | 1.13 | R | 41.4 | 0.07 | NR | Yes |
| Zeng | 2015 | Medicine (Baltimore) | gastric | per 3 cups | 9 | 0 | 0 | 9 | 1.07 | 0.95 | 1.21 | R | NR | 0.004 | 0.1 | No |
| Li | 2015 | BMC Cancer | gastric | per 3 cups | 9 | 0 | 0 | 9 | 1.03 | 0.95 | 1.11 | R | 31.1 | 0.127 | NR | No |
| Malerba | 2013 | Cancer Causes Control | glioma | H/L | 4 | 2 | 0 | 6 | 1.01 | 0.83 | 1.22 | R | 0 | 0.479 | >0.25 | Yes |
| Malerba | 2013 | Cancer Causes Control | glioma | per 1 cup | 3 | 1 | 0 | 4 | 1 | 0.96 | 1.05 | R | 41.9 | 0.16 | >0.25 | Yes |
| Wijarnpreecha | 2017 | Intern Med J | kidney | H/L | 6 | 16 | 0 | 22 | 0.99 | 0.89 | 1.11 | R | 33 | 0.07 | NR | Yes |
| Wang | 2016 | Sci Rep | kidney | H/L | 5 | 0 | 0 | 5 | 0.79 | 0.54 | 1.15 | R | 49.8 | 0.09 | 0.14 | No |
| Huang | 2014 | Int Urol Nephrol | kidney | H/L | 4 | 0 | 0 | 4 | 0.99 | 0.52 | 1.89 | F | 45.2 | 0.121 | NR | No |
| Yu | 2011 | BMC Cancer | kidney | H/L | 4 | 0 | 0 | 4 | 0.74 | 0.46 | 1.03 | R | 64.1 | 0.025 | NR | No |
| Yu | 2011 | BMC Cancer | kidney | per 1 cup | 4 | 0 | 0 | 4 | 0.99 | 0.91 | 1.06 | NR | NR | NR | NR | No |
| Huang | 2014 | Int Urol Nephrol | kidney | per 2 cups | 4 | 0 | 0 | 4 | 0.95 | 0.56 | 1.59 | NR | NR | NR | NR | No |
| Ouyang | 2014 | Int J Clin Exp Med | laryngeal | H/L | 1 | 7 | 0 | 8 | 1.22 | 0.92 | 1.62 | R | 74.1 | <0.001 | 0.256 | Yes |
| Chen | 2014 | PLoS One | laryngeal | H/L | 1 | 5 | 0 | 6 | 1.47 | 1.03 | 2.11 | R | 72.8 | 0.002 | 0.446 | No |
| Turati | 2011 | Ann Oncol | laryngeal | H/L | 0 | 3 | 0 | 3 | 1.56 | 0.6 | 4.02 | R | 70 | 0.036 | >0.05 | No |
| Chen | 2014 | PLoS One | laryngeal | per 1 cup | 0 | 4 | 0 | 4 | 1.22 | 1.04 | 1.54 | NR | NR | NR | NR | No |
| Sang | 2013 | BMC Gastroenterol | liver | H/L | 7 | 9 | 0 | 16 | 0.5 | 0.42 | 0.59 | F | 10.2 | 0.337 | 0.05 | Yes |
| Bravi | 2013 | Clin Gastroenterol Hepatol | liver | H/L | 7 | 8 | 0 | 15 | 0.6 | 0.5 | 0.71 | R | 73.9 | <0.001 | 0.379 | No |
| Yu | 2016 | Sci Rep | liver | H/L | 11 | 0 | 0 | 11 | 0.55 | 0.44 | 0.67 | R | 38 | 0.081 | 0.229 | No |
| Bravi | 2016 | Eur J Cancer Prev | liver | H/L | 11 | 0 | 0 | 11 | 0.5 | 0.43 | 0.58 | R | NR | 0.25 | 0.17 | No |
| Bai | 2016 | Onco Targets Ther | liver | H/L | 3 | 7 | 0 | 10 | 0.21 | 0.18 | 0.25 | R | 94.8 | <0.001 | >0.05 | No |
| Bravi | 2007 | Hepatology | liver | H/L | 4 | 6 | 0 | 10 | 0.45 | 0.38 | 0.53 | F | NR | 0.27 | NR | No |
| Wang | 2016 | Sci Rep | liver | H/L | 9 | 0 | 0 | 9 | 0.46 | 0.37 | 0.57 | F | 0 | 0.44 | 0.23 | No |
| Yu | 2011 | BMC Cancer | liver | H/L | 5 | 0 | 0 | 5 | 0.54 | 0.46 | 0.61 | F | 29.1 | 0.228 | NR | No |
| Godos | 2017 | Nutrients | liver | H/L | 8 | 5 | 0 | 13 | 0.52 | 0.42 | 0.63 | R | 44 | 0.02 | >0.05 | No |
| Bravi | 2007 | Hepatology | liver | per 1 cup | 4 | 6 | 0 | 10 | 0.77 | 0.72 | 0.82 | F | NR | 0.16 | NR | No |
| Yu | 2016 | Sci Rep | liver | per 1 cup | 8 | 0 | 0 | 8 | 0.97 | 0.81 | 0.94 | R | NR | NR | NR | No |
| Yu | 2011 | BMC Cancer | liver | per 1 cup | 5 | 0 | 0 | 5 | 0.83 | 0.78 | 0.87 | NR | NR | NR | NR | No |
| Kennedy | 2017 | BMJ Open | liver | per 2 cups | 10 | 7 | 0 | 17 | 0.65 | 0.59 | 0.72 | R | 58.5 | <0.01 | <0.0001 | Yes |
| Larsson | 2007 | Gastroenterology | liver | per 2 cups | 4 | 5 | 0 | 9 | 0.57 | 0.49 | 0.67 | R | 30.8 | 0.17 | >0.1 | No |
| Wang | 2016 | Sci Rep | liver | per 2 cups | 7 | 0 | 0 | 7 | 0.73 | 0.67 | 0.79 | NR | NR | NR | NR | No |
| Galarraga | 2016 | Cancer Epidemiol Biomarkers Prev | lung | H/L | 8 | 13 | 0 | 21 | 1.09 | 1 | 1.19 | R | 75.1 | <0.001 | <0.001 | Yes |
| Xie | 2016 | Eur J Clin Nutr | lung | H/L | 5 | 12 | 0 | 17 | 1.31 | 1.11 | 1.55 | R | 56.7 | 0.002 | 0.743 | No |
| Tang | 2010 | Lung Cancer | lung | H/L | 5 | 8 | 0 | 13 | 1.27 | 1.04 | 1.54 | R | 58.6 | 0.004 | 0.878 | No |
| Yu | 2011 | BMC Cancer | lung | H/L | 5 | 0 | 0 | 5 | 1.17 | 0.92 | 1.42 | F | 0 | 0.529 | NR | No |
| Wang | 2016 | Sci Rep | lung | H/L | 4 | 0 | 0 | 4 | 2.18 | 1.26 | 3.75 | R | 63.3 | 0.04 | 0.39 | No |
| Galarraga | 2016 | Cancer Epidemiol Biomarkers Prev | lung | per 1 cup | 8 | 13 | 0 | 21 | 1.04 | 1.03 | 1.05 | R | NR | NR | NR | No |
| Tang | 2010 | Lung Cancer | lung | per 2 cups | 2 | 7 | 0 | 9 | 1.14 | 1.04 | 1.26 | R | 41.2 | 0.093 | NR | Yes |
| Han | 2016 | Iran J Public Health | lymphoma | H/L | 3 | 4 | 0 | 7 | 1.05 | 0.89 | 1.23 | R | 44.4 | 0.01 | 0.714 | Yes |
| Wang | 2016 | Sci Rep | lymphoma | H/L | 3 | 0 | 0 | 3 | 1.23 | 0.75 | 2.03 | F | 0 | 0.769 | 0.18 | No |
| Wang | 2016 | Eur J Nutr | melanoma | H/L | 7 | 4 | 0 | 11 | 0.8 | 0.69 | 0.93 | R | 53.5 | 0.014 | 0.738 | No |
| Yew | 2016 | Am J Clin Dermatol | melanoma | H/L | 9 | 2 | 0 | 11 | 0.75 | 0.63 | 0.89 | R | 57.5 | 0.005 | 0.981 | Yes |
| Wang | 2016 | Sci Rep | melanoma | H/L | 6 | 0 | 0 | 6 | 0.89 | 0.8 | 0.99 | F | 0 | 0.42 | 0.4 | No |
| Micek | 2017 | Int J Food Sci Nutr | melanoma | H/L | 4 | 0 | 0 | 4 | 0.79 | 0.62 | 1.01 | R | 47 | 0.09 | NR | No |
| Vaseghi | 2016 | Eur J Cancer Prev | nonmelanoma | H/L | 2 | 3 | 1 | 6 | 0.91 | 0.89 | 0.93 | R | 51.6 | 0.001 | 0.213 | Yes |
| Yu | 2011 | BMC Cancer | nonmelanoma | H/L | 2 | 0 | 0 | 2 | 0.77 | 0.52 | 1.01 | R | 88.2 | 0.004 | NR | No |
| Li | 2016 | Oral Surg Oral Med Oral Pathol Oral Radiol | oral | H/L | 4 | 11 | 0 | 15 | 0.63 | 0.52 | 0.75 | R | 53.1 | 0.008 | 0.289 | Yes |
| Zhang | 2015 | Int J Clin Exp Med | oral | H/L | 3 | 9 | 0 | 12 | 0.69 | 0.54 | 0.89 | R | 65 | 0.001 | 0.556 | No |
| Miranda | 2017 | Med Oral Patol Oral Cir Bucal | oral/pharyngeal | H/L | 4 | 13 | 0 | 17 | 0.69 | 0.57 | 0.84 | R | 50.3 | 0.009 | 0.267 | Yes |
| Turati | 2011 | Ann Oncol | oral/pharyngeal | H/L | 1 | 8 | 0 | 9 | 0.64 | 0.51 | 0.8 | R | 31.3 | 0.149 | >0.05 | No |
| Wang | 2016 | Sci Rep | oral/pharyngeal | H/L | 6 | 0 | 0 | 6 | 0.69 | 0.48 | 0.99 | R | 73.4 | 0.002 | 0.98 | No |
| Berretta | 2018 | Oncotarget | ovarian | H/L | 8 | 0 | 0 | 8 | 1.06 | 0.89 | 1.26 | R | 25 | 0.24 | >0.05 | No |
| Steevens | 2007 | Br J Cancer | ovarian | H/L | 4 | 11 | 0 | 15 | 1.18 | 0.97 | 1.44 | R | 50.5 | 0.013 | NR | Yes |
| Wang | 2016 | Sci Rep | ovarian | H/L | 8 | 0 | 0 | 8 | 1.04 | 0.9 | 1.2 | F | 23.7 | 0.23 | 0.009 | No |
| Yu | 2011 | BMC Cancer | ovarian | H/L | 8 | 0 | 0 | 8 | 1 | 0.83 | 1.17 | R | 54.1 | 0.033 | NR | No |
| Braem | 2012 | Am J Clin Nutr | ovarian | H/L | 7 | 0 | 0 | 7 | 1.13 | 0.89 | 1.43 | R | 50.9 | 0.057 | 0.13 | No |
| Yu | 2011 | BMC Cancer | ovarian | per 1 cup | 8 | 0 | 0 | 8 | 0.96 | 0.93 | 0.99 | NR | NR | NR | NR | No |
| Li | 2019 | Int J Food Sci Nutr | pancreatic | H/L | 13 | 0 | 0 | 13 | 1.08 | 0.94 | 1.25 | F | 45.6 | 0.024 | 0.612 | No |
| Turati | 2012 | Ann Oncol | pancreatic | H/L | 17 | 37 | 0 | 54 | 1.13 | 0.99 | 1.29 | R | 50.2 | <0.001 | 0.9 | Yes |
| Ran | 2016 | Pak J Med Sci | pancreatic | H/L | 20 | 0 | 0 | 20 | 0.75 | 0.63 | 0.86 | R | 37.8 | 0.045 | 0.436 | No |
| Nie | 2016 | Minerva Med | pancreatic | H/L | 20 | 0 | 0 | 20 | 0.99 | 0.81 | 1.21 | R | 47.9 | 0.008 | ? | No |
| Wang | 2016 | Sci Rep | pancreatic | H/L | 15 | 0 | 0 | 15 | 1.02 | 0.87 | 1.18 | F | 16.2 | 0.27 | 0.75 | No |
| Yu | 2011 | BMC Cancer | pancreatic | H/L | 14 | 0 | 0 | 14 | 0.82 | 0.69 | 0.95 | R | 40.6 | 0.057 | NR | No |
| Dong | 2011 | World J Gastroenterol | pancreatic | H/L | 14 | 0 | 0 | 14 | 0.68 | 0.51 | 0.84 | R | 0 | 0.729 | 0.735 | No |
| Nishi | 1996 | Jpn J Clin Oncol | pancreatic | H/L | 6 | 0 | 0 | 6 | 1.21 | 0.98 | 1.5 | R | NR | NR | NR | No |
| Turati | 2012 | Ann Oncol | pancreatic | per 1 cup | 12 | 16 | 0 | 28 | 1.03 | 0.99 | 1.06 | R | 64.8 | <0.001 | NR | Yes |
| Yu | 2011 | BMC Cancer | pancreatic | per 1 cup | 14 | 0 | 0 | 14 | 0.96 | 0.9 | 1.02 | NR | NR | NR | NR | No |
| Ran | 2016 | Pak J Med Sci | pancreatic | per 1 cup | 9 | 0 | 0 | 9 | 0.99 | 0.96 | 1.03 | R | NR | NR | NR | No |
| Yu | 2011 | BMC Cancer | pharyngeal | H/L | 3 | 0 | 0 | 3 | 0.49 | 0.29 | 0.7 | F | 26.1 | 0.258 | NR | No |
| Xia | 2017 | Urol J | prostate | H/L | 14 | 14 | 0 | 28 | 1.07 | 0.96 | 1.18 | R | 52.2 | 0.001 | 0.229 | Yes |
| Zhong | 2014 | Eur J Clin Nutr | prostate | H/L | 12 | 12 | 0 | 24 | 0.94 | 0.85 | 1.05 | R | 53.8 | 0.001 | 0.42 | No |
| Lu | 2014 | Cancer Causes Control | prostate | H/L | 9 | 12 | 0 | 21 | 0.91 | 0.86 | 0.97 | F | 9 | 0.341 | 0.02 | No |
| Wang | 2016 | Sci Rep | prostate | H/L | 14 | 0 | 0 | 14 | 0.89 | 0.84 | 0.93 | F | 30.3 | 0.14 | 0.84 | No |
| Liu | 2015 | Nutr Cancer | prostate | H/L | 13 | 0 | 0 | 13 | 0.9 | 0.85 | 0.95 | F | 17.5 | 0.267 | 0.848 | No |
| Park | 2010 | BJU Int | prostate | H/L | 4 | 8 | 0 | 12 | 1.16 | 1.01 | 1.33 | F | 6.5 | NR | <0.001 | No |
| Cao | 2014 | Carcinogenesis | prostate | H/L | 10 | 0 | 0 | 10 | 0.88 | 0.82 | 0.95 | R | 31.9 | 0.153 | 0.133 | No |
| Huang | 2014 | Int Urol Nephrol | prostate | H/L | 8 | 0 | 0 | 8 | 0.86 | 0.79 | 0.95 | F | 25 | 0.221 | NR | No |
| Yu | 2011 | BMC Cancer | prostate | H/L | 5 | 0 | 0 | 5 | 0.79 | 0.61 | 0.98 | R | 57.1 | 0.053 | NR | No |
| Wang | 2016 | Sci Rep | prostate | per 2 cups | 10 | 0 | 0 | 10 | 0.97 | 0.96 | 0.98 | NR | NR | NR | NR | No |
| Liu | 2015 | Nutr Cancer | prostate | per 2 cups | 9 | 0 | 0 | 9 | 0.98 | 0.96 | 1 | R | NR | NR | NR | No |
| Huang | 2014 | Int Urol Nephrol | prostate | per 2 cups | 8 | 0 | 0 | 8 | 0.98 | 0.93 | 1.03 | NR | NR | NR | NR | No |
| Zhong | 2014 | Eur J Clin Nutr | prostate | per 3 cups | 10 | 8 | 0 | 18 | 0.97 | 0.94 | 1.01 | R | NR | NR | NR | No |
| Gan | 2017 | Oncotarget | rectal | H/L | 15 | 0 | 0 | 15 | 1.06 | 0.95 | 1.19 | R | 13 | 0.308 | 0.822 | Yes |
| Galeone | 2010 | Cancer Causes Control | rectal | H/L | 0 | 14 | 0 | 14 | 0.87 | 0.75 | 1 | R | 36.4 | NR | >0.05 | No |
| Galeone | 2010 | Cancer Causes Control | rectal | per 3 cups | 0 | 10 | 0 | 10 | 0.97 | 0.95 | 0.99 | R | 10.2 | 0.347 | >0.05 | No |
| Gan | 2017 | Oncotarget | rectal | per 4 cups | 14 | 0 | 0 | 14 | 1.05 | 0.97 | 1.13 | R | 11 | 0.333 | 0.384 | Yes |
| Han | 2017 | Int J Environ Res Public Health | thyroid | H/L | 2 | 5 | 0 | 7 | 0.88 | 0.71 | 1.07 | F | 0 | 0.79 | NR | Yes |
| Zeegers | 2001 | Int J Epidemiol | urinary tract | Y/N | 3 | 22 | 0 | 25 | 1.26 | 1.11 | 1.43 | R | NR | <0.01 | >0.51 | No |
|  |  |  |  |  |  |  |  |  |  |  |  |  |  |  |  |  |
| Abbreviation: Co, cohort study; CC, case control study; CS, cross-sectional study; LL, low 95% confidential limit; UL, upper 95% confidential limit; AL, acute leukemia; ALL, acute lymphocytic leukemia; AML, acute myelogenous leukemia; H/L, highest versus lowest intake; Y/N, regular coffee intake versus none; R, random-effects model; F, fixed-effects model; NR, not report. | | | | | | | | | | | | | | | | |

| **ST3 AMSTAR score of included meta-analysis** | | | | | | | | | | | | | | | | | | | | | |
| --- | --- | --- | --- | --- | --- | --- | --- | --- | --- | --- | --- | --- | --- | --- | --- | --- | --- | --- | --- | --- | --- |
| Author | Year | Journal | Q1 | **Q2** | Q3 | **Q4** | Q5 | Q6 | **Q7** | Q8 | **Q9** | Q10 | **Q11** | Q12 | **Q13** | Q14 | **Q15** | Q16 | **Critical** | **Non-critical** | **Level** |
| Botelho[1] | 2006 | Cad Saude Publica | 2 | 2 | 1 | 2 | 2 | 1 | 1 | 2 | 2 | 2 | 1 | 2 | 1 | 1 | 1 | 1 | 3 | 5 | extremely low |
| Galarraga[2] | 2016 | Cancer Epidemiol Biomarkers Prev | 1 | 2 | 1 | 3 | 2 | 2 | 2 | 2 | 2 | 2 | 1 | 2 | 2 | 1 | 1 | 1 | 4 | 5 | extremely low |
| Gan[3] | 2017 | Oncotarget | 2 | 2 | 1 | 3 | 1 | 1 | 2 | 1 | 1 | 2 | 1 | 1 | 1 | 2 | 1 | 1 | 2 | 3 | extremely low |
| Godos[4] | 2017 | Nutrients | 1 | 2 | 1 | 3 | 1 | 1 | 1 | 1 | 1 | 2 | 1 | 1 | 1 | 1 | 1 | 1 | 1 | 1 | low |
| Han[5] | 2017 | Int J Environ Res Public Health | 2 | 2 | 2 | 3 | 1 | 1 | 2 | 3 | 2 | 2 | 1 | 2 | 2 | 1 | 2 | 1 | 5 | 4 | extremely low |
| Han[6] | 2016 | Iran J Public Health | 2 | 2 | 1 | 3 | 1 | 1 | 2 | 3 | 1 | 2 | 1 | 2 | 2 | 1 | 1 | 1 | 3 | 3 | extremely low |
| Huang[7] | 2013 | Chin J Evid-based Med | 1 | 2 | 1 | 3 | 1 | 1 | 2 | 3 | 1 | 2 | 1 | 2 | 1 | 1 | 1 | 2 | 2 | 3 | extremely low |
| Lukic[8] | 2018 | Nutr Cancer | 2 | 2 | 1 | 3 | 2 | 1 | 2 | 3 | 2 | 2 | 1 | 2 | 2 | 1 | 1 | 1 | 4 | 4 | extremely low |
| Jiang[9] | 2013 | Gynecol Oncol | 2 | 2 | 2 | 2 | 1 | 1 | 2 | 3 | 2 | 2 | 1 | 2 | 2 | 1 | 1 | 1 | 5 | 4 | extremely low |
| Kennedy[10] | 2017 | BMJ Open | 2 | 3 | 1 | 3 | 1 | 1 | 2 | 3 | 1 | 2 | 1 | 2 | 2 | 1 | 1 | 1 | 2 | 3 | extremely low |
| Li[11] | 2016 | Oral Surg Oral Med Oral Pathol Oral Radiol | 2 | 2 | 1 | 3 | 2 | 1 | 2 | 1 | 2 | 2 | 1 | 2 | 1 | 1 | 1 | 1 | 3 | 4 | extremely low |
| Li[12] | 2013 | PLoS One | 2 | 2 | 1 | 3 | 2 | 1 | 2 | 3 | 2 | 2 | 1 | 2 | 2 | 1 | 1 | 1 | 4 | 4 | extremely low |
| Li[13] | 2013 | Public Health Nutr | 2 | 2 | 2 | 3 | 1 | 1 | 2 | 2 | 1 | 2 | 1 | 1 | 1 | 1 | 1 | 1 | 2 | 4 | extremely low |
| Malerba[14] | 2013 | Cancer Causes Control | 2 | 2 | 1 | 3 | 1 | 2 | 2 | 1 | 2 | 2 | 1 | 2 | 2 | 2 | 1 | 1 | 4 | 5 | extremely low |
| Miranda[15] | 2017 | Med Oral Patol Oral Cir Bucal | 2 | 2 | 1 | 3 | 1 | 2 | 2 | 2 | 1 | 2 | 1 | 1 | 2 | 1 | 1 | 1 | 3 | 4 | extremely low |
| Ouyang[16] | 2014 | Int J Clin Exp Med | 2 | 2 | 1 | 3 | 1 | 1 | 2 | 3 | 2 | 2 | 1 | 2 | 1 | 1 | 1 | 1 | 3 | 3 | extremely low |
| Sang[17] | 2013 | BMC Gastroenterol | 2 | 2 | 1 | 3 | 2 | 1 | 2 | 2 | 1 | 2 | 1 | 2 | 2 | 1 | 1 | 1 | 3 | 5 | extremely low |
| Steevens[18] | 2007 | Br J Cancer | 2 | 2 | 1 | 2 | 2 | 2 | 2 | 2 | 2 | 2 | 2 | 2 | 2 | 1 | 2 | 1 | 7 | 6 | extremely low |
| Tang[19] | 2010 | Lung Cancer | 1 | 2 | 1 | 3 | 2 | 2 | 1 | 2 | 2 | 2 | 1 | 2 | 2 | 1 | 1 | 1 | 3 | 5 | extremely low |
| Thomopoulos[20] | 2015 | Cancer Epidemiol | 1 | 3 | 1 | 2 | 1 | 1 | 2 | 3 | 1 | 2 | 1 | 1 | 1 | 2 | 1 | 2 | 2 | 3 | extremely low |
| Turati[21] | 2012 | Ann Oncol | 2 | 2 | 1 | 2 | 1 | 1 | 2 | 3 | 2 | 2 | 1 | 2 | 2 | 1 | 1 | 1 | 5 | 3 | extremely low |
| Vaseghi[22] | 2016 | Eur J Cancer Prev | 2 | 2 | 1 | 2 | 2 | 1 | 2 | 3 | 2 | 2 | 1 | 1 | 2 | 1 | 1 | 1 | 5 | 3 | extremely low |
| Wijarnpreecha[23] | 2017 | Intern Med J | 2 | 2 | 1 | 3 | 1 | 1 | 2 | 3 | 1 | 2 | 1 | 1 | 1 | 1 | 1 | 1 | 2 | 2 | extremely low |
| Wu[24] | 2015 | Sci Rep | 2 | 2 | 1 | 3 | 1 | 1 | 2 | 3 | 1 | 2 | 1 | 1 | 2 | 1 | 1 | 1 | 3 | 2 | extremely low |
| Xia[25] | 2017 | Urol J | 2 | 2 | 1 | 2 | 1 | 1 | 2 | 2 | 1 | 2 | 1 | 1 | 1 | 1 | 1 | 1 | 3 | 3 | extremely low |
| Xie[26] | 2014 | Nutrients | 2 | 2 | 1 | 3 | 2 | 1 | 2 | 3 | 2 | 2 | 1 | 2 | 1 | 1 | 1 | 1 | 3 | 4 | extremely low |
| Yew[27] | 2016 | Am J Clin Dermatol | 2 | 3 | 1 | 3 | 1 | 1 | 2 | 3 | 1 | 2 | 1 | 1 | 1 | 1 | 1 | 1 | 1 | 2 | low |
| Zheng[28] | 2013 | Nutr Cancer | 2 | 2 | 1 | 3 | 1 | 2 | 2 | 2 | 2 | 2 | 1 | 2 | 1 | 2 | 1 | 1 | 3 | 6 | extremely low |
| Total |  |  | 5 | 0 | 25 | 0 | 18 | 22 | 3 | 4 | 13 | 0 | 27 | 10 | 13 | 24 | 26 | 26 |  |  |  |
| % |  |  | 17.9 | 0 | 89.3 | 0 | 64.3 | 78.6 | 10.7 | 14.3 | 46.4 | 0 | 96.4 | 35.7 | 46.4 | 85.7 | 92.9 | 92.9 |  |  |  |
|  |  |  |  |  |  |  |  |  |  |  |  |  |  |  |  |  |  |  |  |  |  |
| AMSTAR 2 critical domains included Q2, Q4, Q7, Q9, Q11, Q13, and Q15. Details about the AMSTAR 2 can be found elsewhere (doi: 10.1136/bmj.j4008).  1. Botelho F, Lunet N, Barros H: Coffee and gastric cancer: Systematic review and meta-analysis. Cad Saude Publica 2006, 22(5):889-900.  2. Galarraga V, Boffetta P: Coffee drinking and risk of lung cancer: A meta-analysis. Cancer Epidemiology Biomarkers and Prevention 2016, 25(6):951-957.  3. Gan Y, Wu J, Zhang S, Li L, Cao S, Mkandawire N, Ji K, Herath C, Gao C, Xu H *et al*: Association of coffee consumption with risk of colorectal cancer: A meta-analysis of prospective cohort studies. Oncotarget 2017, 8(12):18699-18711.  4. Godos J, Micek A, Marranzano M, Salomone F, Del Rio D, Ray S: Coffee consumption and risk of biliary tract cancers and liver cancer: A dose–response meta-analysis of prospective cohort studies. Nutrients 2017, 9(9).  5. Han MA, Kim JH: Coffee consumption and the risk of thyroid cancer: A systematic review and meta-analysis. Int J Environ Res Public Health 2017, 14(2).  6. Han TJ, Li JS, Wang L, Xu HZ: Coffee and the risk of lymphoma: A meta-analysis article. Iran J Public Health 2016, 45(9):1126-1135.  7. Huang SJ, Xu H, Wei W: Correlation between coffee and risk of endometrial cancer: A meta-analysis. Chinese Journal of Evidence-Based Medicine 2013, 13(3):313-319.  8. Lukic M, Guha N, Licaj I, van den Brandt PA, Stayner LT, Tavani A, Weiderpass E: Coffee drinking and the risk of endometrial cancer: An updated meta-analysis of observational studies. Nutr Cancer 2018, 70(4):513-528.  9. Jiang W, Wu Y, Jiang X: Coffee and caffeine intake and breast cancer risk: An updated dose-response meta-analysis of 37 published studies. Gynecol Oncol 2013, 129(3):620-629.  10. Kennedy OJ, Roderick P, Buchanan R, Fallowfield JA, Hayes PC, Parkes J: Coffee, including caffeinated and decaffeinated coffee, and the risk of hepatocellular carcinoma: A systematic review and dose-response meta-Analysis. BMJ Open 2017, 7(5).  11. Li YM, Peng J, Li LZ: Coffee consumption associated with reduced risk of oral cancer: A meta-analysis. Oral surgery, oral medicine, oral pathology and oral radiology 2016, 121(4):381-389.  12. Li XJ, Ren ZJ, Qin JW, Zhao JH, Tang JH, Ji MH, Wu JZ: Coffee consumption and risk of breast cancer: An up-to-date meta-analysis. PLoS One 2013, 8(1).  13. Li G, Ma D, Zhang Y, Zheng W, Wang P: Coffee consumption and risk of colorectal cancer: a meta-analysis of observational studies. Public Health Nutr 2013, 16(2):346-357.  14. Malerba S, Galeone C, Pelucchi C, Turati F, Hashibe M, La Vecchia C, Tavani A: A meta-analysis of coffee and tea consumption and the risk of glioma in adults. Cancer Causes Control 2013, 24(2):267-276.  15. Miranda J, Monteiro L, Albuquerque R, Pacheco JJ, Khan Z, Lopez-Lopez J, Warnakulasuryia S: Coffee is protective against oral and pharyngeal cancer: A systematic review and meta-analysis. Med Oral Patol Oral Cir Bucal 2017, 22(5):e554-e561.  16. Ouyang Z, Wang Z, Jin J: Association between tea and coffee consumption and risk of laryngeal cancer: A meta-analysis. Int J Clin Exp Med 2014, 7(12):5192-5200.  17. Sang LX, Chang B, Li XH, Jiang M: Consumption of coffee associated with reduced risk of liver cancer: A meta-analysis. BMC Gastroenterol 2013, 13(1).  18. Steevens J, Schouten LJ, Verhage BAJ, Goldbohm RA, Van Den Brandt PA: Tea and coffee drinking and ovarian cancer risk: Results from the Netherlands Cohort Study and a meta-analysis. Br J Cancer 2007, 97(9):1291-1294.  19. Tang N, Wu Y, Ma J, Wang B, Yu R: Coffee consumption and risk of lung cancer: A meta-analysis. Lung Cancer 2010, 67(1):17-22.  20. Thomopoulos TP, Ntouvelis E, Diamantaras AA, Tzanoudaki M, Baka M, Hatzipantelis E, Kourti M, Polychronopoulou S, Sidi V, Stiakaki E *et al*: Maternal and childhood consumption of coffee, tea and cola beverages in association with childhood leukemia: a meta-analysis. Cancer Epidemiol 2015, 39(6):1047-1059.  21. Turati F, Galeone C, Edefonti V, Ferraroni M, Lagiou P, La Vecchia C, Tavani A: A meta-analysis of coffee consumption and pancreatic cancer. Ann Oncol 2012, 23(2):311-318.  22. Vaseghi G, Haghjoo-Javanmard S, Naderi J, Eshraghi A, Mahdavi M, Mansourian M: Coffee consumption and risk of nonmelanoma skin cancer: A dose-response meta-analysis. Eur J Cancer Prev 2018, 27(2):164-170.  23. Wijarnpreecha K, Thongprayoon C, Thamcharoen N, Panjawatanan P, Cheungpasitporn W: Association between coffee consumption and risk of renal cell carcinoma: A meta-analysis. Intern Med J 2017, 47(12):1422-1432.  24. Wu W, Tong Y, Zhao Q, Yu G, Wei X, Lu Q: Coffee consumption and bladder cancer: A meta-analysis of observational studies. Sci Rep 2015, 5:9051.  25. Xia J, Chen J, Xue JX, Yang J, Wang ZJ: An up-to-date meta-analysis of coffee consumption and risk of prostate cancer. Urology journal 2017, 14(5):4079-4088.  26. Xie F, Wang D, Huang Z, Guo Y: Coffee consumption and risk of gastric cancer: A large updated meta-analysis of prospective studies. Nutrients 2014, 6(9):3734-3746.  27. Yew YW, Lai YC, Schwartz RA: Coffee consumption and melanoma: A systematic review and meta-analysis of observational studies. Am J Clin Dermatol 2016, 17(2):113-123.  28. Zheng JS, Yang J, Fu YQ, Huang T, Huang YJ, Li D: Effects of green tea, black tea, and coffee consumption on the risk of esophageal cancer: A systematic review and meta-analysis of observational studies. Nutr Cancer 2013, 65(1):1-16. | | | | | | | | | | | | | | | | | | | | | |

| **ST4 Description of meta-analyses only including cohort studies of coffee consumption and cancer incidence with more than one meta-analysis** | | | | | | |
| --- | --- | --- | --- | --- | --- | --- |
| Author | Year | Journal | **Cancer type** | **Unit of comparison** | **No of studies** | Included |
| Yu | 2011 | BMC Cancer | **ALL** | **H/L** | **2** | Yes |
| Yu | 2011 | BMC Cancer | **all cancers** | **H/L** | **40** | No |
| Yu | 2011 | BMC Cancer | **all cancers** | **per 1 cup** | **40** | No |
| Godos | 2017 | Nutrients | **Biliary tract** | **H/L** | **3** | Yes |
| Wang | 2016 | Sci Rep | **bladder** | **H/L** | **10** | Yes |
| Wu | 2015 | Sci Rep | **bladder** | **H/L** | **5** | No |
| Zhou | 2012 | Prev Med | **bladder** | **H/L** | **5** | No |
| Huang | 2014 | Int Urol Nephrol | **bladder** | **H/L** | **5** | No |
| Yu | 2011 | BMC Cancer | **bladder** | **H/L** | **5** | No |
| Bai | 2014 | World J Surg Oncol | **bladder** | **H/L** | **2** | No |
| Wu | 2015 | Sci Rep | **bladder** | **per 1 cup** | **5** | No |
| Yu | 2011 | BMC Cancer | **bladder** | **per 1 cup** | **5** | No |
| Huang | 2014 | Int Urol Nephrol | **bladder** | **per 2 cups** | **5** | No |
| Zhou | 2012 | Prev Med | **bladder** | **per 2 cups** | **4** | No |
| Lafranconi | 2018 | Nutrients | **breast** | **H/L** | **16** | No |
| Jiang | 2013 | Gynecol Oncol | **breast** | **H/L** | **17** | No |
| Wang | 2016 | Sci Rep | **breast** | **H/L** | **17** | Yes |
| Li | 2013 | PLoS One | **breast** | **H/L** | **16** | No |
| Yu | 2011 | BMC Cancer | **breast** | **H/L** | **11** | No |
| Tang | 2009 | Am J Obstet Gynecol | **breast** | **H/L** | **9** | No |
| Yu | 2011 | BMC Cancer | **breast** | **per 1 cup** | **11** | No |
| Li | 2013 | PLoS One | **breast** | **per 2 cups** | **15** | Yes |
| Jiang | 2013 | Gynecol Oncol | **breast** | **per 2 cups** | **12** | No |
| Tang | 2009 | Am J Obstet Gynecol | **breast** | **per 2 cups** | **8** | No |
| Gan | 2017 | Oncotarget | **colon** | **H/L** | **16** | Yes |
| Wang | 2016 | Sci Rep | **colon** | **H/L** | **9** | No |
| Gan | 2017 | Oncotarget | **colon** | **per 4 cups** | **15** | Yes |
| Wang | 2016 | Sci Rep | **colorectal** | **H/L** | **21** | Yes |
| Gan | 2017 | Oncotarget | **colorectal** | **H/L** | **19** | No |
| Li | 2013 | Public Health Nutr | **colorectal** | **H/L** | **16** | No |
| Yu | 2011 | BMC Cancer | **colorectal** | **H/L** | **15** | No |
| Je | 2009 | Int J Cancer | **colorectal** | **H/L** | **12** | No |
| Giovannucci | 1998 | Am J Epidemiol | **colorectal** | **H/L** | **5** | No |
| Akter | 2016 | Jpn J Clin Oncol | **colorectal** | **H/L** | **5** | No |
| Yu | 2011 | BMC Cancer | **colorectal** | **per 1 cup** | **15** | No |
| Vieira | 2017 | Ann Oncol | **colorectal** | **per 1 cup** | **14** | No |
| Gan | 2017 | Oncotarget | **colorectal** | **per 4 cups** | **17** | Yes |
| Lafranconi | 2017 | Nutrients | **endometrial** | **H/L** | **12** | No |
| Lukic | 2018 | Nutr Cancer | **endometrial** | **H/L** | **12** | No |
| Zhou | 2015 | Sci Rep | **endometrial** | **H/L** | **14** | Yes |
| Wang | 2016 | Sci Rep | **endometrial** | **H/L** | **12** | No |
| Huang | 2013 | Chin J Cancer Prev Treat | **endometrial** | **H/L** | **10** | No |
| Je | 2012 | Int J Cancer | **endometrial** | **H/L** | **6** | No |
| Yu | 2011 | BMC Cancer | **endometrial** | **H/L** | **4** | No |
| Bravi | 2009 | Am J Obstet Gynecol | **endometrial** | **H/L** | **2** | No |
| Yang | 2015 | Am J Clin Nutr | **endometrial** | **per 1 cup** | **8** | No |
| Je | 2012 | Int J Cancer | **endometrial** | **per 1 cup** | **6** | No |
| Yu | 2011 | BMC Cancer | **endometrial** | **per 1 cup** | **4** | No |
| Wang | 2016 | Sci Rep | **endometrial** | **per 2 cups** | **11** | No |
| Huang | 2013 | Chin J Evid-based Med | **endometrial** | **per 2 cups** | **7** | Yes |
| Zhang | 2018 | Medicine (Baltimore) | **esophageal** | **H/L** | **2** | No |
| Wang | 2016 | Sci Rep | **esophageal** | **H/L** | **6** | Yes |
| Yu | 2011 | BMC Cancer | **esophageal** | **H/L** | **2** | No |
| Yu | 2011 | BMC Cancer | **esophageal** | **per 1 cup** | **2** | No |
| Zheng | 2013 | Nutr Cancer | **esophageal** | **H/L** | **4** | No |
| Turati | 2011 | Ann Oncol | **esophageal(ESCC)** | **H/L** | **1** | No |
| Deng | 2016 | Nutr Cancer | **gastric** | **H/L** | **13** | Yes |
| Li | 2015 | BMC Cancer | **gastric** | **H/L** | **13** | No |
| Wang | 2016 | Sci Rep | **gastric** | **H/L** | **12** | No |
| Xie | 2014 | Nutrients | **gastric** | **H/L** | **12** | No |
| Zeng | 2015 | Medicine (Baltimore) | **gastric** | **H/L** | **9** | No |
| Liu | 2015 | PLoS One | **gastric** | **H/L** | **9** | No |
| Shen | 2015 | Clin Res Hepatol Gastroenterol | **gastric** | **H/L** | **8** | No |
| Fang | 2015 | Eur J Cancer | **gastric** | **H/L** | **8** | No |
| Yu | 2011 | BMC Cancer | **gastric** | **H/L** | **8** | No |
| Botelho | 2006 | Cad Saude Publica | **gastric** | **H/L** | **7** | No |
| Xie | 2016 | Asia Pac J Clin Nutr | **gastric** | **H/L** | **6** | No |
| Yu | 2011 | BMC Cancer | **gastric** | **per 1 cup** | **8** | No |
| Liu | 2015 | PLoS One | **gastric** | **per 1 cup** | **5** | No |
| Xie | 2014 | Nutrients | **gastric** | **per 2 cups** | **9** | No |
| Li | 2015 | BMC Cancer | **gastric** | **per 3 cups** | **9** | Yes |
| Zeng | 2015 | Medicine (Baltimore) | **gastric** | **per 3 cups** | **9** | No |
| Malerba | 2013 | Cancer Causes Control | **glioma** | **H/L** | **3** | Yes |
| Malerba | 2013 | Cancer Causes Control | **glioma** | **per 1 cup** | **3** | Yes |
| Wijarnpreecha | 2017 | Intern Med J | **kidney** | **H/L** | **6** | Yes |
| Wang | 2016 | Sci Rep | **kidney** | **H/L** | **5** | No |
| Yu | 2011 | BMC Cancer | **kidney** | **H/L** | **4** | No |
| Yu | 2011 | BMC Cancer | **kidney** | **per 1 cup** | **4** | No |
| Chen | 2014 | PLoS One | **laryngeal** | **H/L** | **1** | No |
| Ouyang | 2014 | Int J Clin Exp Med | **laryngeal** | **H/L** | **1** | Yes |
| Bravi | 2016 | Eur J Cancer Prev | **liver** | **H/L** | **11** | Yes |
| Yu | 2016 | Sci Rep | **liver** | **H/L** | **11** | No |
| Godos | 2017 | Nutrients | **liver** | **H/L** | **8** | No |
| Wang | 2016 | Sci Rep | **liver** | **H/L** | **9** | No |
| Bravi | 2013 | Clin Gastroenterol Hepatol | **liver** | **H/L** | **7** | No |
| Sang | 2013 | BMC Gastroenterol | **liver** | **H/L** | **7** | No |
| Yu | 2011 | BMC Cancer | **liver** | **H/L** | **5** | No |
| Bravi | 2007 | Hepatology | **liver** | **H/L** | **4** | No |
| Larsson | 2007 | Gastroenterology | **liver** | **H/L** | **4** | No |
| Bai | 2016 | Onco Targets Ther | **liver** | **H/L** | **3** | No |
| Yu | 2016 | Sci Rep | **liver** | **per 1 cup** | **8** | No |
| Yu | 2011 | BMC Cancer | **liver** | **per 1 cup** | **5** | No |
| Kennedy | 2017 | BMJ Open | **liver** | **per 2 cups** | **10** | Yes |
| Wang | 2016 | Sci Rep | **liver** | **per 2 cups** | **7** | No |
| Larsson | 2007 | Gastroenterology | **liver** | **per 2 cups** | **4** | No |
| Galarraga | 2016 | Cancer Epidemiol Biomarkers Prev | **lung** | **H/L** | **8** | Yes |
| Xie | 2016 | Eur J Clin Nutr | **lung** | **H/L** | **5** | No |
| Tang | 2010 | Lung Cancer | **lung** | **H/L** | **5** | No |
| Yu | 2011 | BMC Cancer | **lung** | **H/L** | **5** | No |
| Wang | 2016 | Sci Rep | **lung** | **H/L** | **4** | No |
| Wang | 2012 | Lung Cancer | **lung** | **per 1 cup** | **9** | No |
| Galarraga | 2016 | Cancer Epidemiol Biomarkers Prev | **lung** | **per 1 cup** | **8** | No |
| Yu | 2011 | BMC Cancer | **lung** | **per 1 cup** | **5** | No |
| Tang | 2010 | Lung Cancer | **lung** | **per 2 cups** | **2** | Yes |
| Han | 2016 | Iran J Public Health | **lymphoma** | **H/L** | **3** | Yes |
| Wang | 2016 | Sci Rep | **lymphoma** | **H/L** | **3** | No |
| Micek | 2018 | Int J Food Sci Nutr | **melanoma** | **H/L** | **4** | No |
| Yew | 2016 | Am J Clin Dermatol | **melanoma** | **H/L** | **10** | Yes |
| Micek | 2017 | Int J Food Sci Nutr | **melanoma** | **H/L** | **4** | No |
| Wang | 2016 | Eur J Nutr | **melanoma** | **H/L** | **8** | No |
| Liu | 2016 | PLoS One | **melanoma** | **H/L** | **7** | No |
| Wang | 2016 | Sci Rep | **melanoma** | **H/L** | **6** | No |
| Liu | 2016 | PLoS One | **melanoma** | **per 1 cup** | **7** | No |
| Yu | 2011 | BMC Cancer | **nonmelanoma** | **H/L** | **2** | No |
| Vaseghi | 2016 | Eur J Cancer Prev | **nonmelanoma** | **H/L** | **2** | Yes |
| Caini | 2017 | Eur J Nutr | **nonmelanoma** | **H/L** | **2** | No |
| Yu | 2011 | BMC Cancer | **nonmelanoma** | **per 1 cup** | **2** | No |
| Vaseghi | 2016 | Eur J Cancer Prev | **nonmelanoma** | **per 1 cup** | **2** | No |
| Li | 2016 | Oral Surg Oral Med Oral Pathol Oral Radiol | **oral** | **H/L** | **4** | Yes |
| Zhang | 2015 | Int J Clin Exp Med | **oral** | **H/L** | **3** | No |
| Miranda | 2017 | Med Oral Patol Oral Cir Bucal | **oral/pharynx** | **H/L** | **4** | No |
| Wang | 2016 | Sci Rep | **oral/pharynx** | **H/L** | **6** | Yes |
| Turati | 2011 | Ann Oncol | **oral/pharynx** | **H/L** | **1** | No |
| Berretta | 2018 | Oncotarget | **ovarian** | **H/L** | **8** | No |
| Wang | 2016 | Sci Rep | **ovarian** | **H/L** | **8** | Yes |
| Yu | 2011 | BMC Cancer | **ovarian** | **H/L** | **8** | No |
| Braem | 2012 | Am J Clin Nutr | **ovarian** | **H/L** | **7** | No |
| Steevens | 2007 | Br J Cancer | **ovarian** | **H/L** | **5** | No |
| Yu | 2011 | BMC Cancer | **ovarian** | **per 1 cup** | **8** | No |
| Li | 2019 | Int J Food Sci Nutr | **pancreatic** | **H/L** | **13** | No |
| Ran | 2016 | Pak J Med Sci | **pancreatic** | **H/L** | **20** | Yes |
| Nie | 2016 | Minerva Med | **pancreatic** | **H/L** | **20** | No |
| Turati | 2012 | Ann Oncol | **pancreatic** | **H/L** | **17** | No |
| Wang | 2016 | Sci Rep | **pancreatic** | **H/L** | **15** | No |
| Dong | 2011 | World J Gastroenterol | **pancreatic** | **H/L** | **14** | No |
| Yu | 2011 | BMC Cancer | **pancreatic** | **H/L** | **14** | No |
| Nishi | 1996 | Jpn J Clin Oncol | **pancreatic** | **H/L** | **6** | No |
| Yu | 2011 | BMC Cancer | **pancreatic** | **per 1 cup** | **14** | No |
| Turati | 2012 | Ann Oncol | **pancreatic** | **per 1 cup** | **12** | Yes |
| Yu | 2011 | BMC Cancer | **pharyngeal** | **H/L** | **3** | No |
| Yu | 2011 | BMC Cancer | **pharyngeal** | **per 1 cup** | **3** | No |
| Wang | 2016 | Sci Rep | **prostate** | **H/L** | **14** | Yes |
| Xia | 2017 | Urol J | **prostate** | **H/L** | **13** | No |
| Liu | 2015 | Nutr Cancer | **prostate** | **H/L** | **13** | No |
| Zhong | 2014 | Eur J Clin Nutr | **prostate** | **H/L** | **12** | No |
| Cao | 2014 | Carcinogenesis | **prostate** | **H/L** | **10** | No |
| Lu | 2014 | Cancer Causes Control | **prostate** | **H/L** | **9** | No |
| Huang | 2014 | Int Urol Nephrol | **prostate** | **H/L** | **8** | No |
| Yu | 2011 | BMC Cancer | **prostate** | **H/L** | **5** | No |
| Park | 2010 | BJU Int | **prostate** | **H/L** | **4** | No |
| Huang | 2014 | Int Urol Nephrol | **prostate** | **H/L** | **4** | No |
| Yu | 2011 | BMC Cancer | **prostate** | **per 1 cup** | **5** | No |
| Wang | 2016 | Sci Rep | **prostate** | **per 2 cups** | **10** | No |
| Liu | 2015 | Nutr Cancer | **prostate** | **per 2 cups** | **9** | No |
| Huang | 2014 | Int Urol Nephrol | **prostate** | **per 2 cups** | **8** | No |
| Zhong | 2014 | Eur J Clin Nutr | **prostate** | **per 3 cups** | **12** | No |
| Discacciati | 2014 | Ann Oncol | **prostate** | **per 3 cups** | **5** | No |
| Je | 2009 | Int J Cancer | **rectal** | **H/L** | **10** | No |
| Wang | 2016 | Sci Rep | **rectal** | **H/L** | **8** | No |
| Wang | 2016 | Sci Rep | **renal** | **H/L** | **5** | No |
| Gan | 2017 | Oncotarget | **rectal** | **H/L** | **15** | Yes |
| Vieira | 2017 | Ann Oncol | **rectal** | **per 1 cup** | **15** | No |
| Gan | 2017 | Oncotarget | **rectal** | **per 4 cups** | **14** | Yes |
| Han | 2017 | Int J Environ Res Public Health | **thyroid** | **H/L** | **2** | Yes |
| Zeegers | 2001 | Int J Epidemiol | **urinary tract** | **Y/N** | **3** | No |

| ST5 Description, evaluation of bias and heterogeneity in 34 associations of coffee intake and cancer incidence only including meta-analyses of cohort studies | | | | | | | | | | | | | | | | |
| --- | --- | --- | --- | --- | --- | --- | --- | --- | --- | --- | --- | --- | --- | --- | --- | --- |
|  |  |  |  |  | Summary relative risk (95% CI) | | |  |  |  |  |  |  |  |  |  |
| Author, year | Cancer type | Unit of compari-son | No of studi-es | No of cases | Fixed-effects | Random-effects | Largest study | Fixed-effects P value | Random-effects P value | 95%PI | I^2^ (95%CI) | P for Q | Egger's test | O | E | P for TES |
| Yu, 2011 [1] | ALL | H/L | 2 | 375 | 0.65 (0.47, 0.92) | 0.77 (0.35, 1.67) | 0.62 (0.44, 0.88) | 0.013 | 0.502 | / | / | 0.207 | / | 1 | 0.3 | 0.166 |
| Godos, 2017 [2] | Biliary tract cancer | H/L | 10 | 1,201 | 0.93 (0.69, 1.26) | 0.93 (0.69, 1.26) | 0.94 (0.64, 1.37) | 0.657 | 0.657 | (0.14, 6.43) | 0 (0, 89.6) | 0.68 | 0.243 | 0 | 0.24 | - |
| Wang, 2016 [3] | Bladder cancer | H/L | 10 | 1,563 | 1.13 (0.94, 1.34) | 1.21 (0.95, 1.55) | 0.95 (0.69, 1.30) | 0.191 | 0.131 | (0.65, 2.27) | 40 (0, 71.4) | 0.091 | 0.01 | 0 | 2.68 | - |
| Wang, 2016 [3] | Breast cancer | H/L | 17 | 29,178 | 0.99 (0.94, 1.04) | 0.99 (0.94, 1.04) | 0.98 (0.89, 1.07) | 0.608 | 0.608 | (0.94, 1.04) | 0 (0, 51.1) | 0.557 | 0.739 | 1 | 2.6 | - |
| Gan, 2017 [4] | Colon cancer | H/L | 16 | 13,853 | 0.91 (0.84, 0.98) | 0.92 (0.83, 1.02) | 0.92 (0.80, 1.06) | 0.011 | 0.097 | (0.72, 1.18) | 29.9 (0, 61.6) | 0.124 | 0.699 | 3 | 3.14 | - |
| Wang, 2016 [3] | Colorectal cancer | H/L | 21 | 23,289 | 0.96 (0.91, 1.02) | 0.96 (0.89, 1.04) | 1.06 (0.95, 1.18) | 0.156 | 0.321 | (0.80, 1.16) | 24.4 (0, 55.6) | 0.151 | 0.774 | 3 | 4.72 | - |
| Zhou, 2015 [5] | Endometrial cancer | H/L | 14 | 10,100 | 0.80 (0.74, 0.86) | 0.76 (0.69, 0.84) | 0.92 (0.82, 1.04) | 1.28E-09 | 1.89E-07 | (0.60, 0.97) | 28.6 (0, 62.3) | 0.15 | 0.04 | 7 | 4.1 | 0.089 |
| Wang, 2016 [3] | Esophageal cancer | H/L | 6 | 1,068 | 0.86 (0.71, 1.04) | 0.86 (0.71, 1.04) | 0.95 (0.70, 1.29) | 0.123 | 0.123 | (0.65, 1.13) | 0 (0, 74.6) | 0.645 | 0.699 | 1 | 0.82 | 0.831 |
| Deng, 2016 [6] | Gastric cancer | H/L | 13 | 3,484 | 1.16 (1.03, 1.32) | 1.15 (0.98, 1.36) | 1.09 (0.84, 1.43) | 0.019 | 0.083 | (0.78, 1.72) | 31.2 (0, 64.5) | 0.133 | 0.53 | 3 | 2.83 | 0.909 |
| Malerba, 2013 [7] | Glioma | H/L | 4 | 1,369 | 0.98 (0.80, 1.20) | 0.98 (0.80, 1.20) | 0.98 (0.70, 1.41) | 0.85 | 0.876 | (0.57, 1.70) | 6.4 (0, 85.7) | 0.361 | 0.162 | 0 | 0.85 | - |
| Wijarnpreecha, 2017 [8] | Kidney cancer | H/L | 6 | 2,736 | 0.88 (0.77, 1.01) | 0.88 (0.70, 1.10) | 0.84 (0.67, 1.05) | 0.065 | 0.272 | (0.48, 1.62) | 50.6 (0, 80.3) | 0.072 | 0.964 | 0 | 1.53 | - |
| Ouyang, 2014 [9] | Laryngeal cancer | H/L | 1 | 178 | 1.01 (0.71, 1.44) | 1.01 (0.71, 1.44) | 1.01 (0.71, 1.44) | 0.956 | 0.956 | / | / | / | / | 0 | 0.05 | - |
| Bravi, 2016 [10] | Liver cancer | H/L | 11 | 3,275 | 0.50 (0.44, 0.57) | 0.50 (0.43, 0.58) | 0.46 (0.34, 0.63) | 1.66E-24 | 9.86E-19 | (0.36, 0.68) | 20 (0, 59.6) | 0.253 | 0.76 | 9 | 1.97 | 0 |
| Galarraga, 2016 [11] | Lung cancer | H/L | 8 | 11,145 | 1.07 (1.05, 1.10) | 1.33 (1.16, 1.52) | 1.04 (1.01, 1.07) | 3.92E-25 | 3.192E-05 | (0.92, 1.91) | 88 (78.6, 93.3) | 0 | 0.05 | 5 | 4.06 | 0.506 |
| Han, 2016 [12] | Lymphoma | H/L | 3 | 219 | 1.30 (0.81, 2.10) | 1.30 (0.81, 2.10) | 1.09 (0.58, 2.06) | 0.282 | 0.282 | (0.06, 29.2) | 0 (0, 89.6) | 0.699 | 0.123 | 0 | 0.3 | - |
| Yew, 2016 [13] | Melanoma | H/L | 9 | 941 | 0.77 (0.70, 0.85) | 0.77 (0.68, 0.87) | 0.80 (0.69, 0.93) | 3.79E-07 | 1.555E-05 | (0.61, 0.96) | 16.2 (0, 58.3) | 0.298 | 0.796 | 4 | 1.63 | 0.04 |
| Vaseghi, 2016 [14] | Nonmelanoma | H/L | 2 | 26,078 | 0.91 (0.82, 1.02) | 0.92 (0.80, 1.05) | 0.90 (0.80, 1.01) | 0.114 | 0.198 | / | / | 0.308 | / | 0 | 0.23 | - |
| Li, 2016 [15] | Oral cancer | H/L | 4 | 1,758 | 0.66 (0.56, 0.78) | 0.66 (0.45, 0.98) | 0.51 (0.40, 0.64) | 4.76E-07 | 0.038 | (0.12, 3.60) | 78.8 (43.1, 92.1) | 0.003 | 0.988 | 2 | 2.04 | - |
| Wang, 2016 [3] | Oral/pharynx cancer | H/L | 6 | 1,910 | 0.69 (0.60, 0.80) | 0.69 (0.48, 0.99) | 0.51 (0.41, 0.64) | 9.32E-07 | 0.042 | (0.24, 2.02) | 73.7 (39.8, 88.5) | 0.002 | 0.998 | 2 | 2.36 | - |
| Wang, 2016 [3] | Ovarian cancer | H/L | 8 | 3,026 | 1.03 (0.89, 1.20) | 1.08 (0.90, 1.31) | 0.77 (0.59, 1.02) | 0.653 | 0.412 | (0.70, 1.68) | 32.6 (0, 70.1) | 0.168 | 0.012 | 0 | 3.33 | - |
| Ran, 2016 [16] | Pancreatic cancer | H/L | 20 | 2,872 | 0.96 (0.84, 1.11) | 0.91 (0.75, 1.11) | 1.07 (0.86, 1.33) | 0.607 | 0.354 | (0.55, 1.50) | 28.2 (0, 58.4) | 0.117 | 0.355 | 3 | 4.06 | - |
| Wang, 2016 [3] | Prostate cancer | H/L | 14 | 37,362 | 0.88 (0.84, 0.93) | 0.88 (0.81, 0.96) | 0.94 (0.87, 1.02) | 6.80E-06 | 0.003 | (0.72, 1.07) | 30.8 (0, 63.5) | 0.13 | 0.826 | 3 | 3.34 | - |
| Gan, 2017 [4] | Rectal cancer | H/L | 15 | 6,200 | 1.07 (0.97, 1.18) | 1.06 (0.95, 1.19) | 1.20 (1.00, 1.44) | 0.185 | 0.285 | (0.86, 1.31) | 13 (0, 50.9) | 0.308 | 0.822 | 1 | 3.13 | - |
| Han, 2017 [17] | Thyroid cancer | H/L | 2 | 265 | 1.00 (0.75, 1.33) | 1.00 (0.75, 1.33) | 1.00 (0.68, 1.48) | 1 | 1 | / | / | 1 | / | 0 | 0.1 | - |
| Li, 2013 [18] | Breast cancer | Per 1 cup | 15 | 27,446 | 0.99 (0.98, 1.00) | 0.99 (0.98, 1.00) | 0.99 (0.98, 1.01) | 0.03816 | 0.0381553 | (0.98, 1.00) | 0 (0, 53.6) | 0.557 | 0.534 | 1 | 2.29 | - |
| Gan, 2017 [4] | Colon cancer | Per 1 cup | 15 | 13,650 | 0.98 (0.97, 0.99) | 0.98 (0.97, 1.00) | 0.97 (0.95, 0.99) | 0.0006 | 0.0190135 | (0.95, 1.02) | 22.6 (0, 58.1) | 0.203 | 0.818 | 3 | 3 | - |
| Gan, 2017 [4] | Colorectal cancer | Per 1 cup | 17 | 22,360 | 0.99 (0.98, 1.00) | 0.99 (0.98, 1.01) | 0.97 (0.96, 0.99) | 0.0102 | 0.292 | (0.96, 1.03) | 34.4 (0, 63.4) | 0.081 | 0.434 | 3 | 4.78 | - |
| Huang, 2013 [19] | Endometrial cancer | Per 1 cup | 7 | 3,571 | 0.94 (0.91, 0.96) | 0.93 (0.90, 0.97) | 0.94 (0.90, 0.97) | 6.63E-07 | 0.0003213 | (0.85, 1.02) | 35 (0, 72.5) | 0.161 | 0.274 | 3 | 1.3 | 0.098 |
| Li, 2015 [20] | Gastric cancer | Per 1 cup | 9 | 2,298 | 1.01 (0.99, 1.03) | 1.01 (0.99, 1.03) | 1.02 (0.98, 1.06) | 0.551 | 0.452 | (0.96, 1.06) | 23.1 (0, 63.6) | 0.238 | 0.085 | 1 | 1.89 | - |
| Malerba, 2013 [7] | Glioma | Per 1 cup | 3 | 1,369 | 1.01 (0.97, 1.04) | 1.01 (0.96, 1.07) | 1.00 (0.96, 1.05) | 0.709 | 0.693 | (0.58, 1.76) | 53.8 (0, 86.8) | 0.115 | 0.692 | 0 | 0.68 | - |
| Kennedy, 2017 [21] | Liver cancer | Per 1 cup | 10 | 2,905 | 0.86 (0.83, 0.88) | 0.84 (0.80, 0.88) | 0.90 (0.86, 0.95) | 4.19E-25 | 2.55E-14 | (0.75, 0.94) | 41.2 (0, 71.9) | 0.083 | 0.014 | 8 | 3.46 | 0.003 |
| Tang, 2010 [22] | Lung cancer | Per 1 cup | 2 | 235 | 1.14 (1.05, 1.23) | 1.14 (1.00, 1.31) | 1.07 (0.96, 1.19) | 0.00202 | 0.0492661 | / | / | 0.103 | / | 1 | 0.63 | 0.573 |
| Turati, 2012 [23] | Pancreatic cancer | Per 1 cup | 12 | 1,465 | 0.99 (0.96, 1.03) | 1.00 (0.95, 1.05) | 0.94 (0.88, 1.00) | 0.651 | 0.962 | (0.88, 1.13) | 39.2 (0, 69.2) | 0.079 | 0.34 | 2 | 3.36 | - |
| Gan, 2017 [4] | Rectal cancer | Per 1 cup | 14 | 6,134 | 1.01 (0.99, 1.03) | 1.01 (0.99, 1.03) | 0.99 (0.96, 1.02) | 0.263 | 0.211 | (0.98, 1.05) | 11.3 (0, 50.4) | 0.323 | 0.383 | 0 | 3.16 | - |

1. Yu X, Bao Z, Zou J, Dong J: Coffee consumption and risk of cancers: a meta-analysis of cohort studies. BMC cancer 2011, 11:96.

2. Godos J, Micek A, Marranzano M, Salomone F, Del Rio D, Ray S: Coffee consumption and risk of biliary tract cancers and liver cancer: A dose–response meta-analysis of prospective cohort studies. Nutrients 2017, 9(9).

3. Wang A, Wang S, Zhu C, Huang H, Wu L, Wan X, Yang X, Zhang H, Miao R, He L *et al*: Coffee and cancer risk: A meta-analysis of prospective observational studies. Scientific reports 2016, 6:33711.

4. Gan Y, Wu J, Zhang S, Li L, Cao S, Mkandawire N, Ji K, Herath C, Gao C, Xu H *et al*: Association of coffee consumption with risk of colorectal cancer: a meta-analysis of prospective cohort studies. Oncotarget 2017, 8(12):18699-18711.

5. Zhou Q, Luo ML, Li H, Li M, Zhou JG: Coffee consumption and risk of endometrial cancer: a dose-response meta-analysis of prospective cohort studies. Scientific reports 2015, 5:13410.

6. Deng W, Yang H, Wang J, Cai J, Bai Z, Song J, Zhang Z: Coffee consumption and the risk of incident gastric cancer - A meta-analysis of prospective cohort studies. Nutrition and cancer 2016, 68(1):40-47.

7. Malerba S, Galeone C, Pelucchi C, Turati F, Hashibe M, La Vecchia C, Tavani A: A meta-analysis of coffee and tea consumption and the risk of glioma in adults. Cancer causes & control : CCC 2013, 24(2):267-276.

8. Wijarnpreecha K, Thongprayoon C, Thamcharoen N, Panjawatanan P, Cheungpasitporn W: Association between coffee consumption and risk of renal cell carcinoma: A Meta-analysis. Internal medicine journal 2017.

9. Ouyang Z, Wang Z, Jin J: Association between tea and coffee consumption and risk of laryngeal cancer: a meta-analysis. International journal of clinical and experimental medicine 2014, 7(12):5192-5200.

10. Bravi F, Tavani A, Bosetti C, Boffetta P, La Vecchia C: Coffee and the risk of hepatocellular carcinoma and chronic liver disease: a systematic review and meta-analysis of prospective studies. European journal of cancer prevention : the official journal of the European Cancer Prevention Organisation (ECP) 2016.

11. Galarraga V, Boffetta P: Coffee drinking and risk of lung cancer-a meta-analysis. Cancer epidemiology, biomarkers & prevention : a publication of the American Association for Cancer Research, cosponsored by the American Society of Preventive Oncology 2016, 25(6):951-957.

12. Han T, Li J, Wang L, Xu H: Coffee and the risk of lymphoma: a meta-analysis article. Iranian journal of public health 2016, 45(9):1126-1135.

13. Yew YW, Lai YC, Schwartz RA: Coffee consumption and melanoma: a systematic review and meta-analysis of observational studies. American journal of clinical dermatology 2016, 17(2):113-123.

14. Vaseghi G, Haghjoo-Javanmard S, Naderi J, Eshraghi A, Mahdavi M, Mansourian M: Coffee consumption and risk of nonmelanoma skin cancer: a dose-response meta-analysis. European journal of cancer prevention : the official journal of the European Cancer Prevention Organisation (ECP) 2016.

15. Li YM, Peng J, Li LZ: Coffee consumption associated with reduced risk of oral cancer: a meta-analysis. Oral surgery, oral medicine, oral pathology and oral radiology 2016, 121(4):381-389.e381.

16. Ran HQ, Wang JZ, Sun CQ: Coffee Consumption and Pancreatic Cancer Risk: An Update Meta-analysis of Cohort Studies. Pakistan journal of medical sciences 2016, 32(1):253-259.

17. Han MA, Kim JH: Coffee Consumption and the Risk of Thyroid Cancer: A Systematic Review and Meta-Analysis. International journal of environmental research and public health 2017, 14(2).

18. Li XJ, Ren ZJ, Qin JW, Zhao JH, Tang JH, Ji MH, Wu JZ: Coffee consumption and risk of breast cancer: an up-to-date meta-analysis. PloS one 2013, 8(1):e52681.

19. Huang SJ, Xu H, Wei W: Correlation between coffee and risk of endometrial cancer: A meta-analysis. Chinese Journal of Evidence-Based Medicine 2013, 13(3):313-319.

20. Li L, Gan Y, Wu C, Qu X, Sun G, Lu Z: Coffee consumption and the risk of gastric cancer: a meta-analysis of prospective cohort studies. BMC cancer 2015, 15:733.

21. Kennedy OJ, Roderick P, Buchanan R, Fallowfield JA, Hayes PC, Parkes J: Coffee, including caffeinated and decaffeinated coffee, and the risk of hepatocellular carcinoma: a systematic review and dose-response meta-analysis. BMJ open 2017, 7(5):e013739.

22. Tang N, Wu Y, Ma J, Wang B, Yu R: Coffee consumption and risk of lung cancer: a meta-analysis. Lung cancer (Amsterdam, Netherlands) 2010, 67(1):17-22.

23. Turati F, Galeone C, Edefonti V, Ferraroni M, Lagiou P, La Vecchia C, Tavani A: A meta-analysis of coffee consumption and pancreatic cancer. Annals of oncology : official journal of the European Society for Medical Oncology 2012, 23(2):311-318.

| **ST6 Summary of evidence grading for meta-analyses associating coffee intake and cancer incidence** | | |  |
| --- | --- | --- | --- |
| **Level of evidence** | **Criteria used** | **Decreased risk** | **Increased risk** |
| Convincing | P in random-effects model =< 0.001 Number of cases > 1000 I^2^ =<50% 95% predictive intervals exclude the null value Small study effects P > 0.1 Excess significance bias P > 0.1 | None | None |
| Highly suggestive | P in random-effects model =< 0.001 Number of cases > 1000 I^2^= <75% | Liver cancer (Per 1 cup); Liver cancer (H/L); Endometrial cancer (H/L); Endometrial cancer (Per 1 cup) | None |
| Suggestive | P in random-effects model =< 0.001 Number of cases > 500 | Melanoma (H/L) | Lung cancer (H/L) |
| Weak | P in random-effects model <= 0.05 | Breast cancer (Per 1 cup); Colon cancer (Per 1 cup); Oral cancer (H/L); Oral/pharynx cancer (H/L); Prostate cancer (H/L) | Lung cancer (Per 1 cup) |
| No association | P in random-effects model > 0.05 | Pancreatic cancer (H/L); Breast cancer (H/L); Colon cancer (H/L); Gastric cancer (H/L); Esophageal cancer (H/L); Bladder cancer (H/L); Nonmelanoma (H/L); Rectal cancer (Per 1 cup); Kidney cancer (H/L); Lymphoma (H/L); Rectal cancer (H/L); Colorectal cancer (Per 1 cup); Colorectal cancer (H/L); Pancreatic cancer (H/L); Ovarian cancer (H/L); Gastric cancer (Per 1 cup); Glioma (H/L); ALL (H/L); Laryngeal cancer (H/L); Pancreatic cancer (Per 1 cup); Thyroid cancer (H/L); Biliary tract cancer (H/L). | |
