# Supplementary material for: Coffee drinking and cancer risk: an umbrella review of meta-analyses of observational studies
Source: BMC Cancer. 2020 Feb 5;20:101. doi: 10.1186/s12885-020-6561-9 (PMC7003434; doi:10.1186/s12885-020-6561-9)

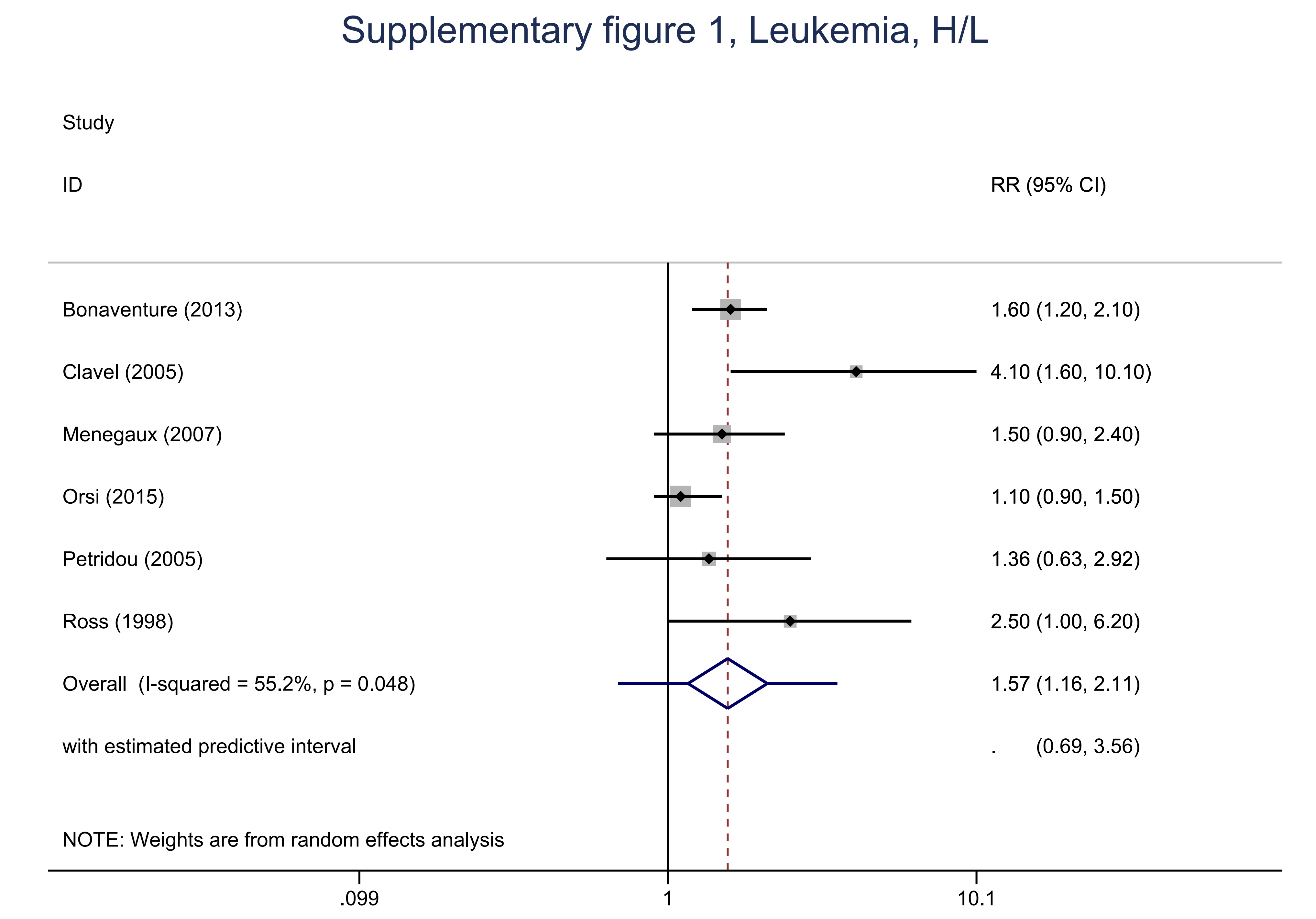
Supplementary figure 1, Leukemia, H/L
Study
ID
RR (95% CI)
Bonaventure (2013)
1.60 (1.20, 2.10)
Clavel (2005)
4.10 (1.60, 10.10)
Menegaux (2007)
1.50 (0.90, 2.40)
Orsi (2015)
1.10 (0.90, 1.50)
Petridou (2005)
1.36 (0.63, 2.92)
Ross (1998)
2.50 (1.00, 6.20)
Overall (I-squared = 55.2%, p = 0.048)
1.57 (1.16, 2.11)
with estimated predictive interval
. (0.69, 3.56)
NOTE: Weights are from random effects analysis
.099
1
10.1

Supplementary figure 2, Acute lymphocytic leukemia, H/L

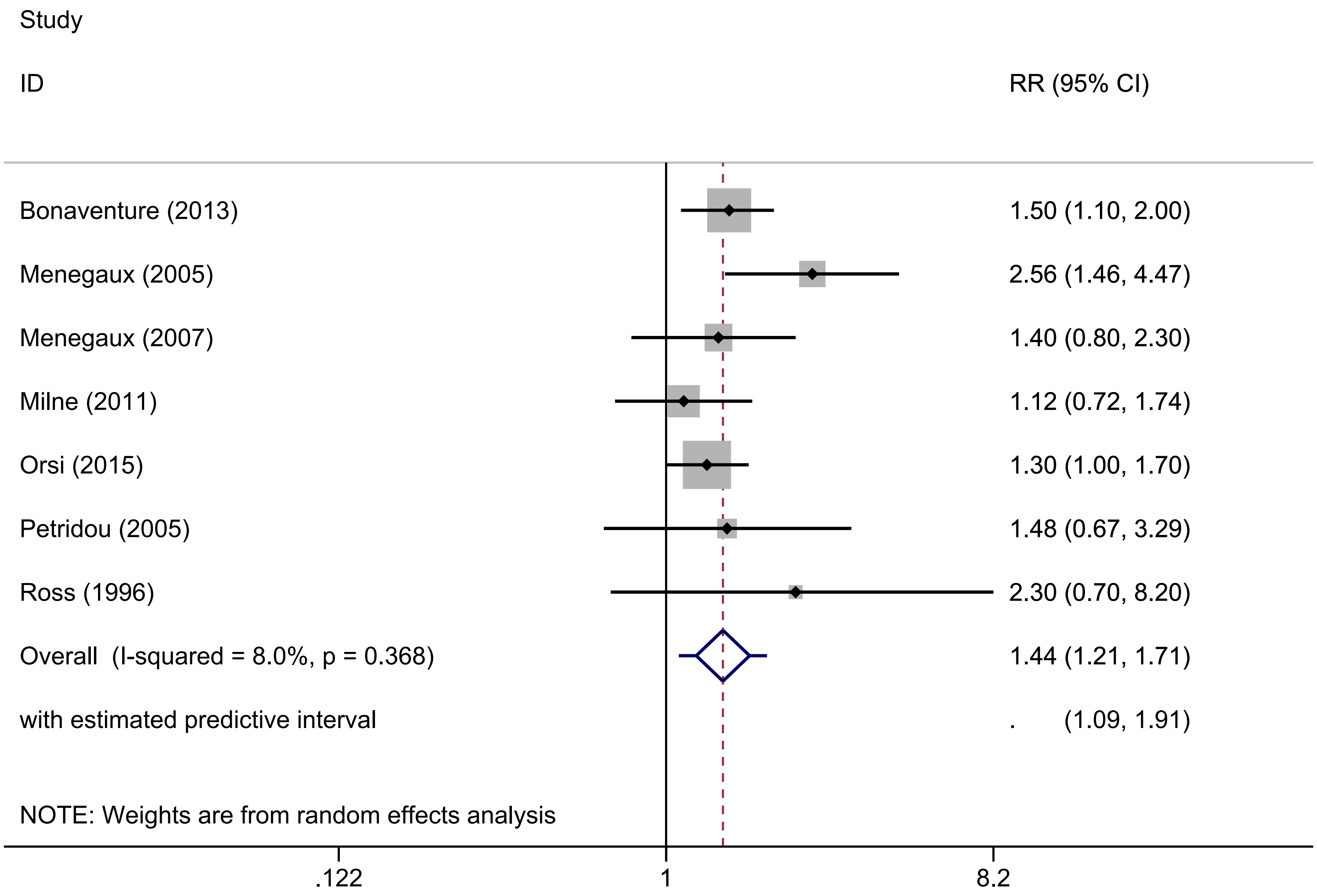

Supplementary figure 3, Acute myelogenous leukemia, H/L

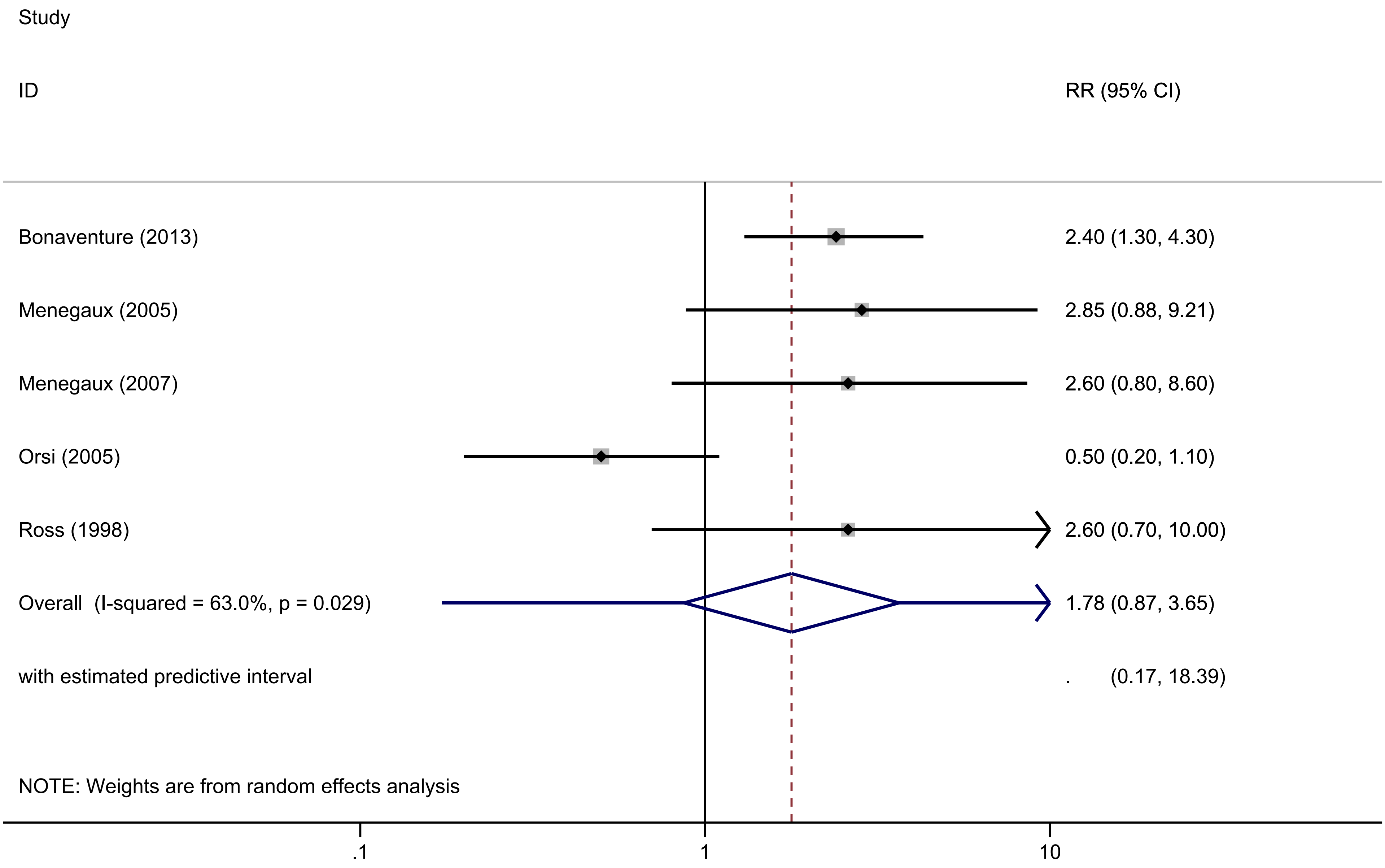

Supplementary figure 4, Bladder cancer, H/L

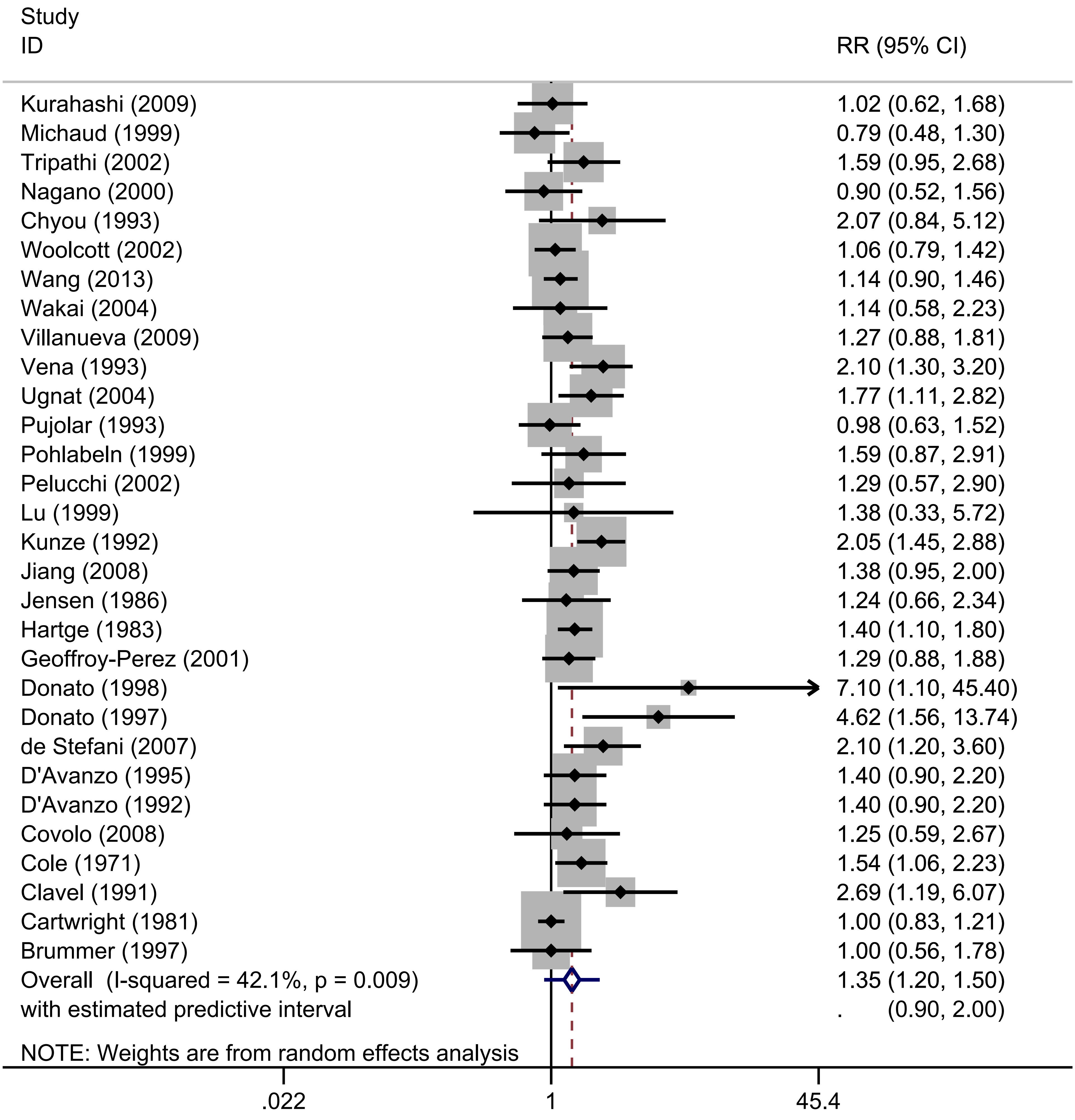

Supplementary figure 5, Breast cancer, H/L

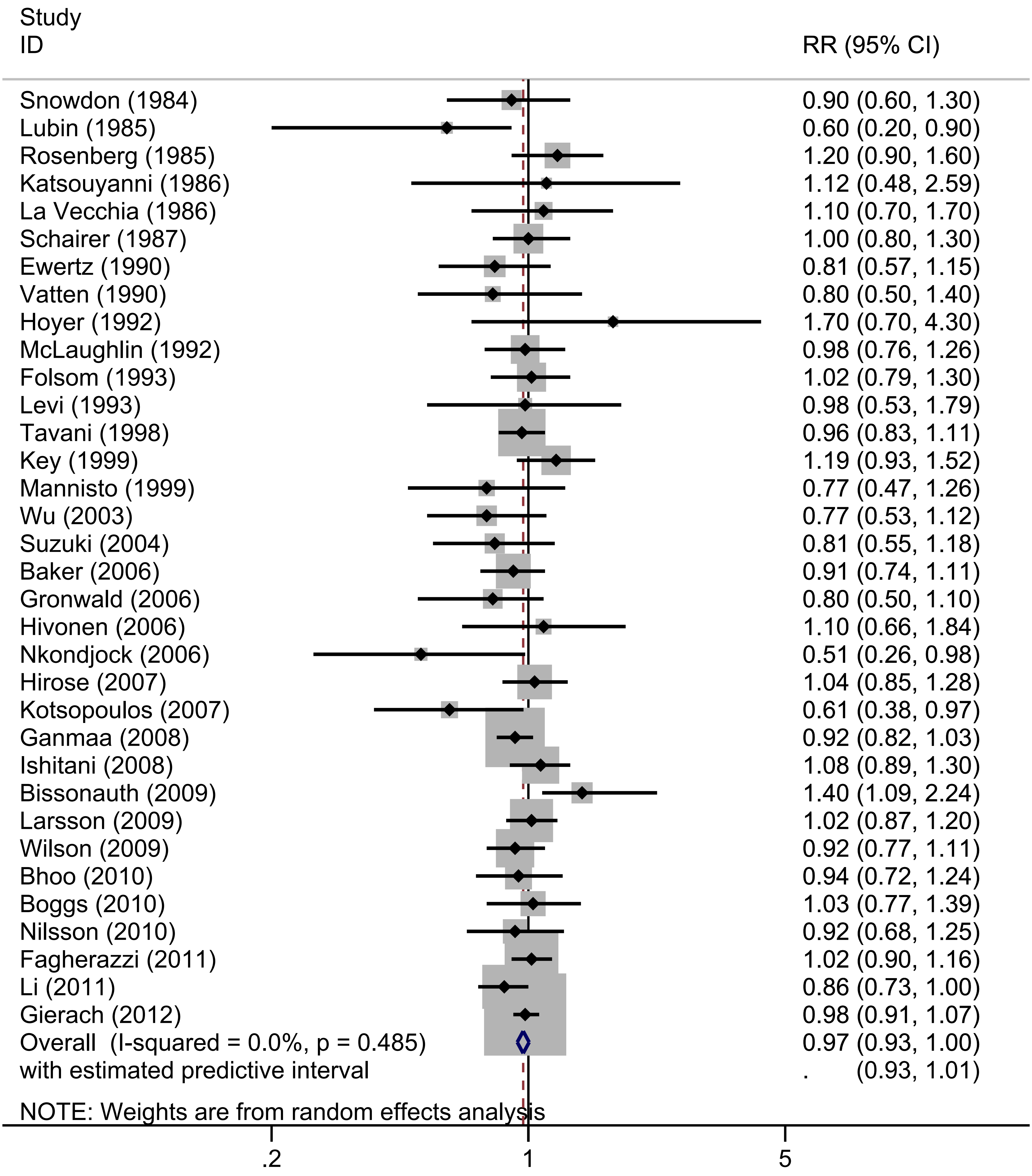

Supplementary figure 6, Breast cancer, Per 1 cup

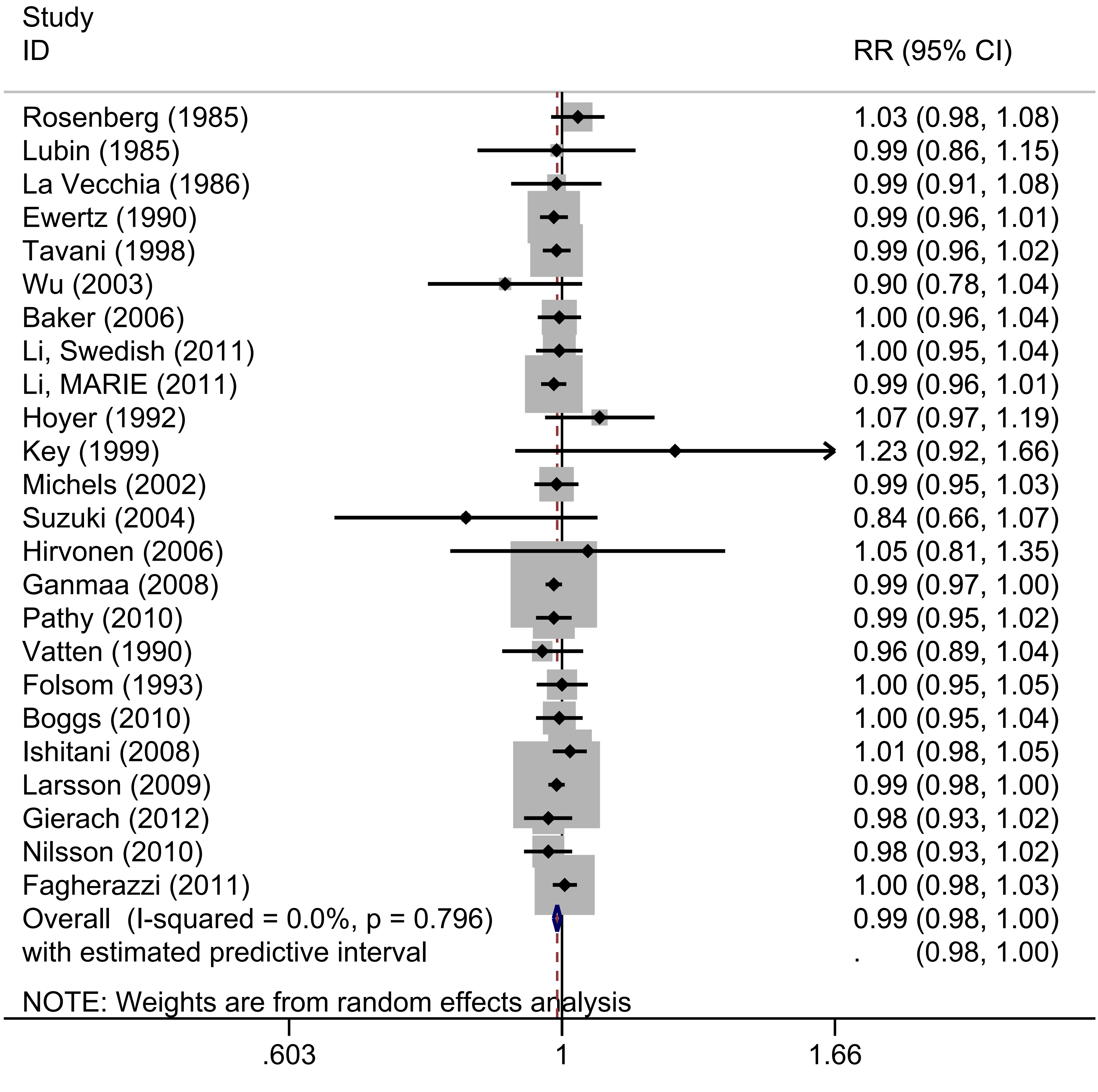

Supplementary figure 7, Colon cancer, H/L

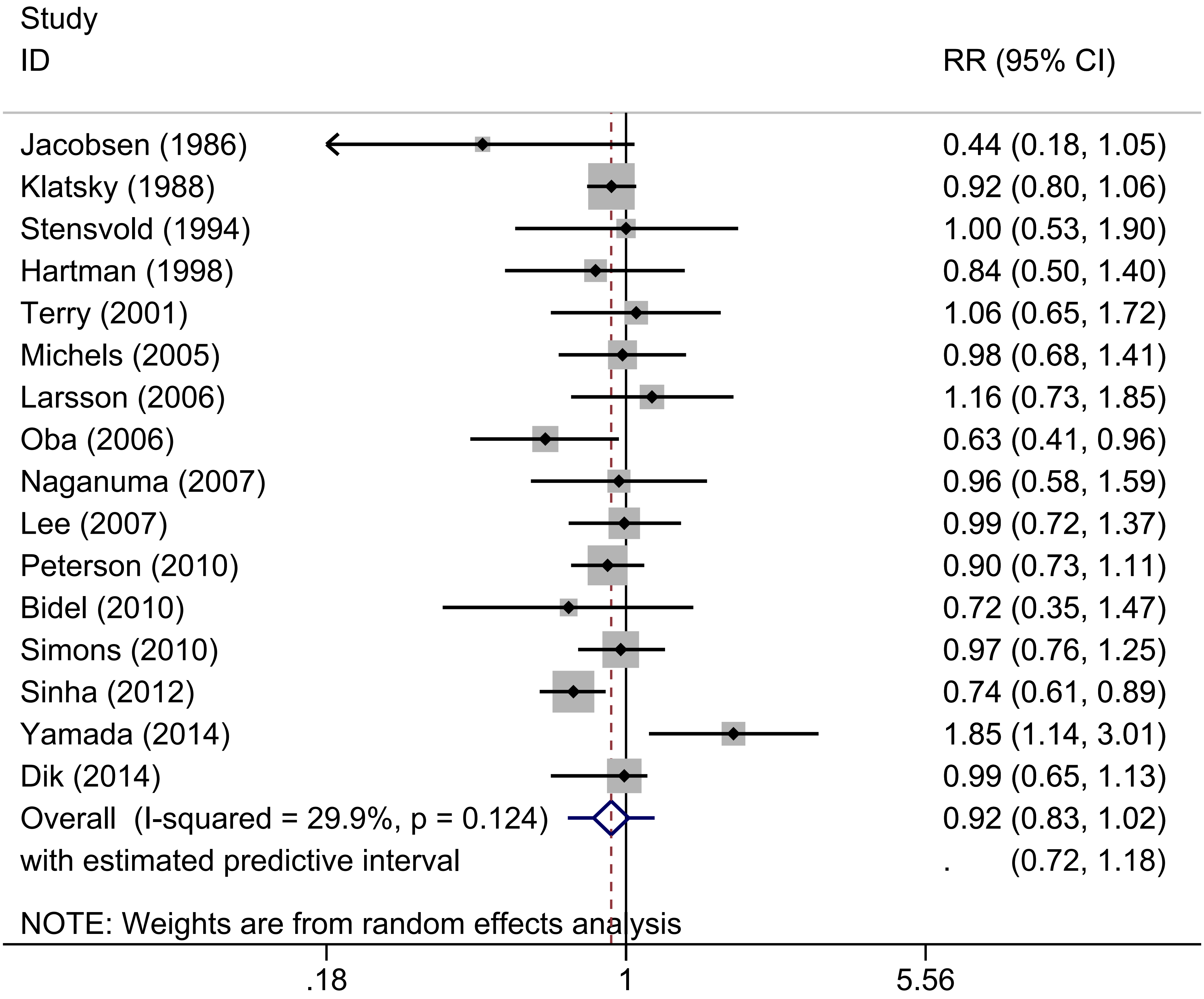

Supplementary figure 8, Colon cancer, Per 1 cup

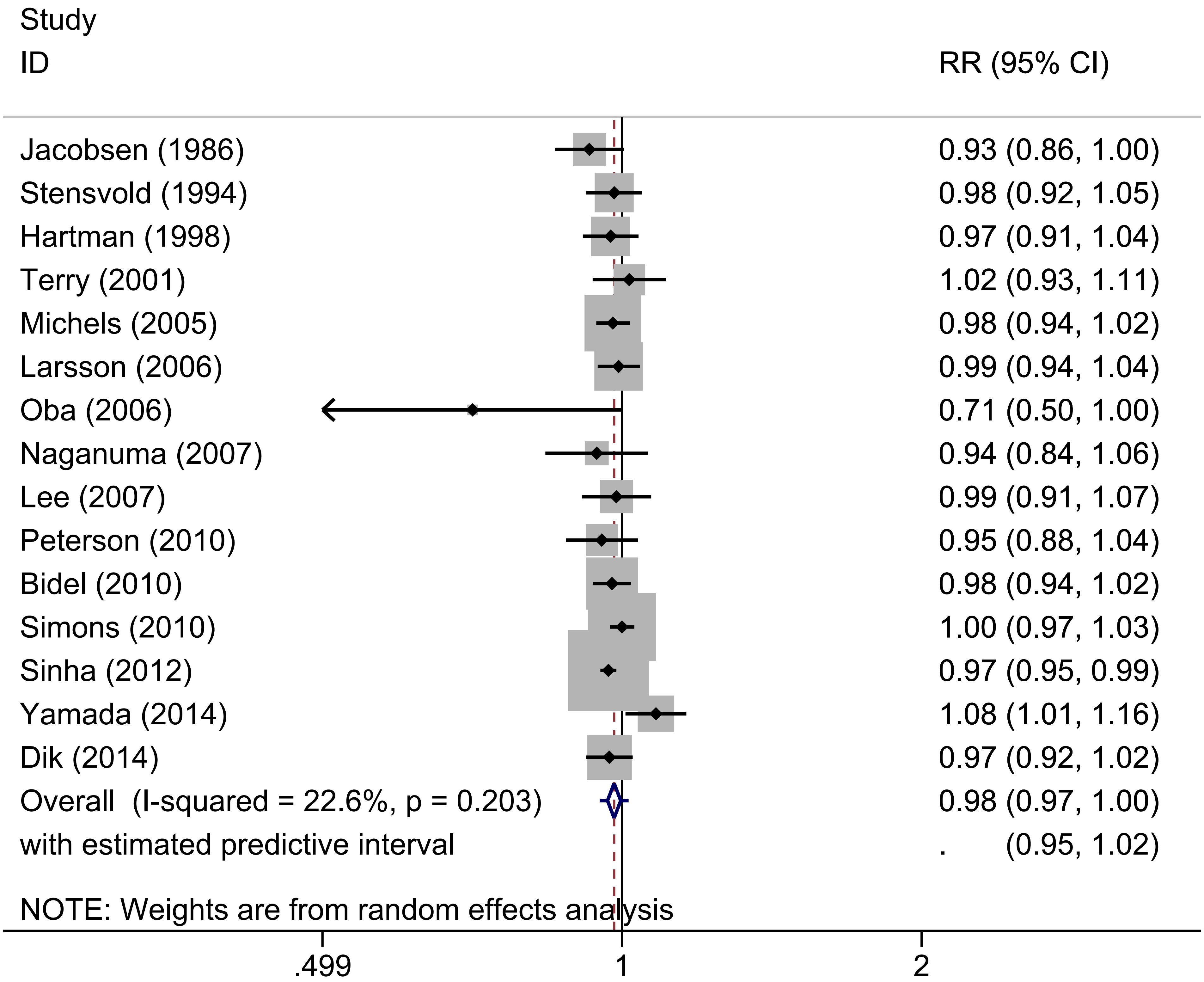

Supplementary figure 9, Colorectal cancer, H/L

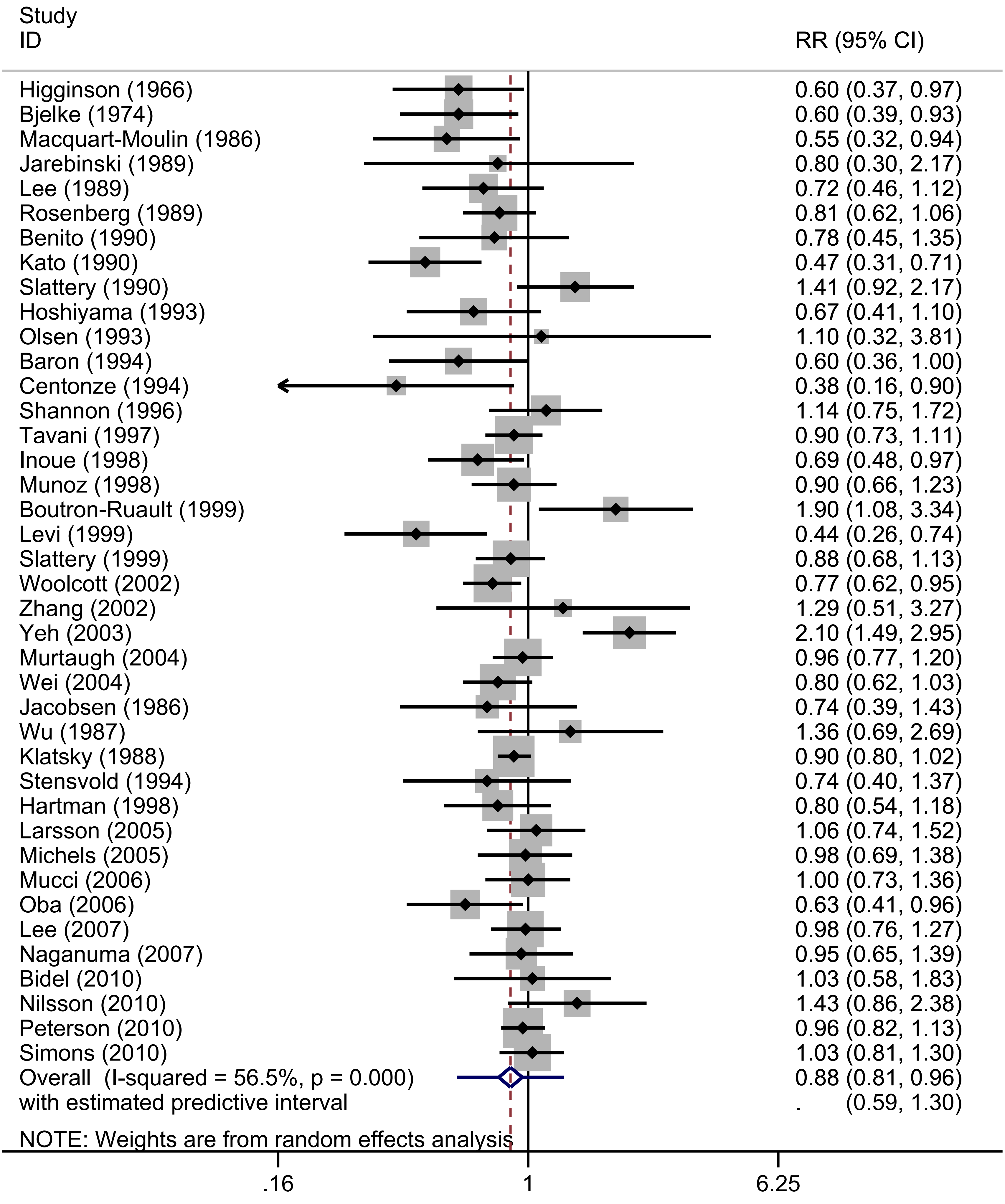

Supplementary figure 10, Colorectal cancer, Per 1 cup

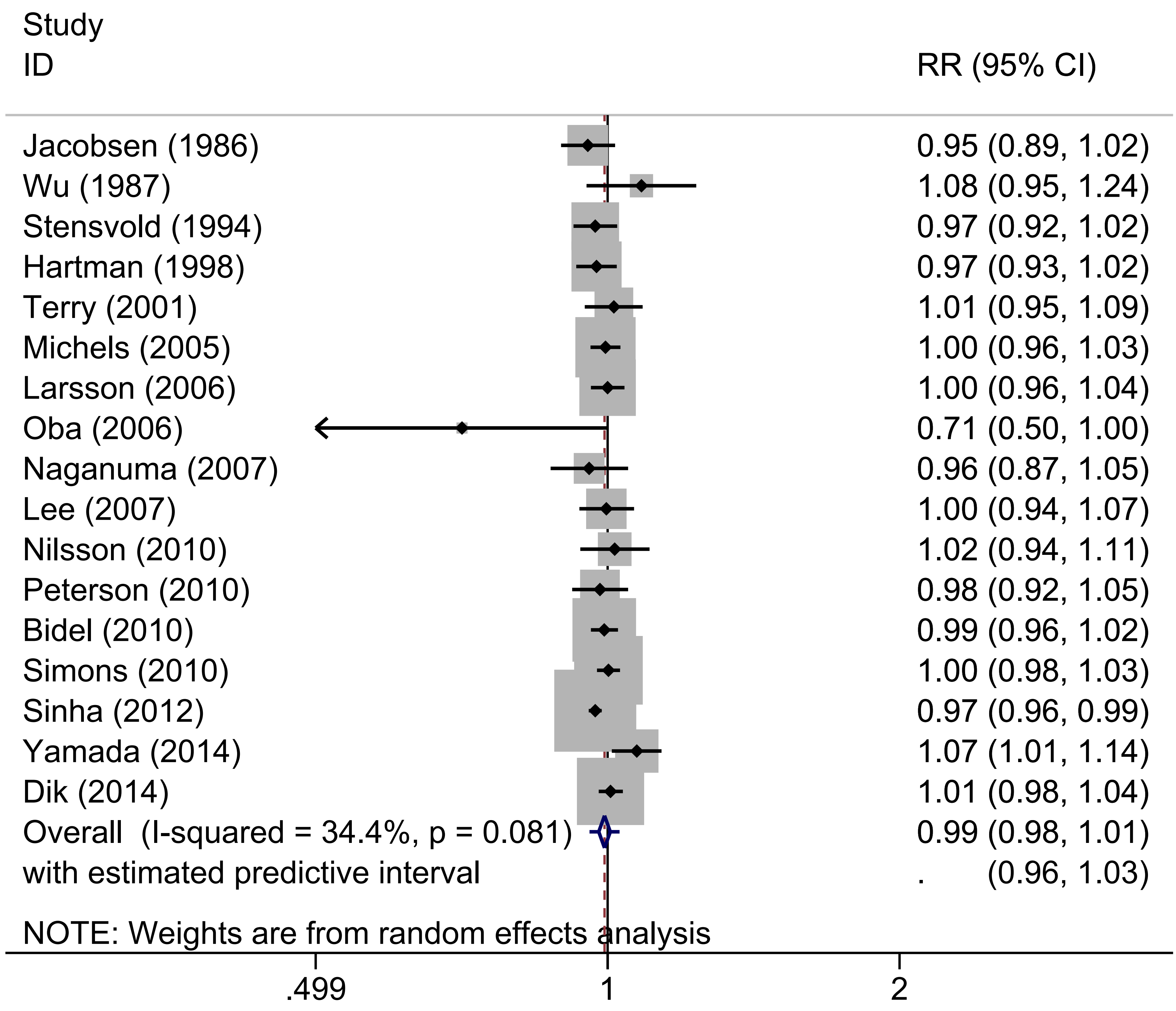

Supplementary figure 11, Endometrial cancer, H/L

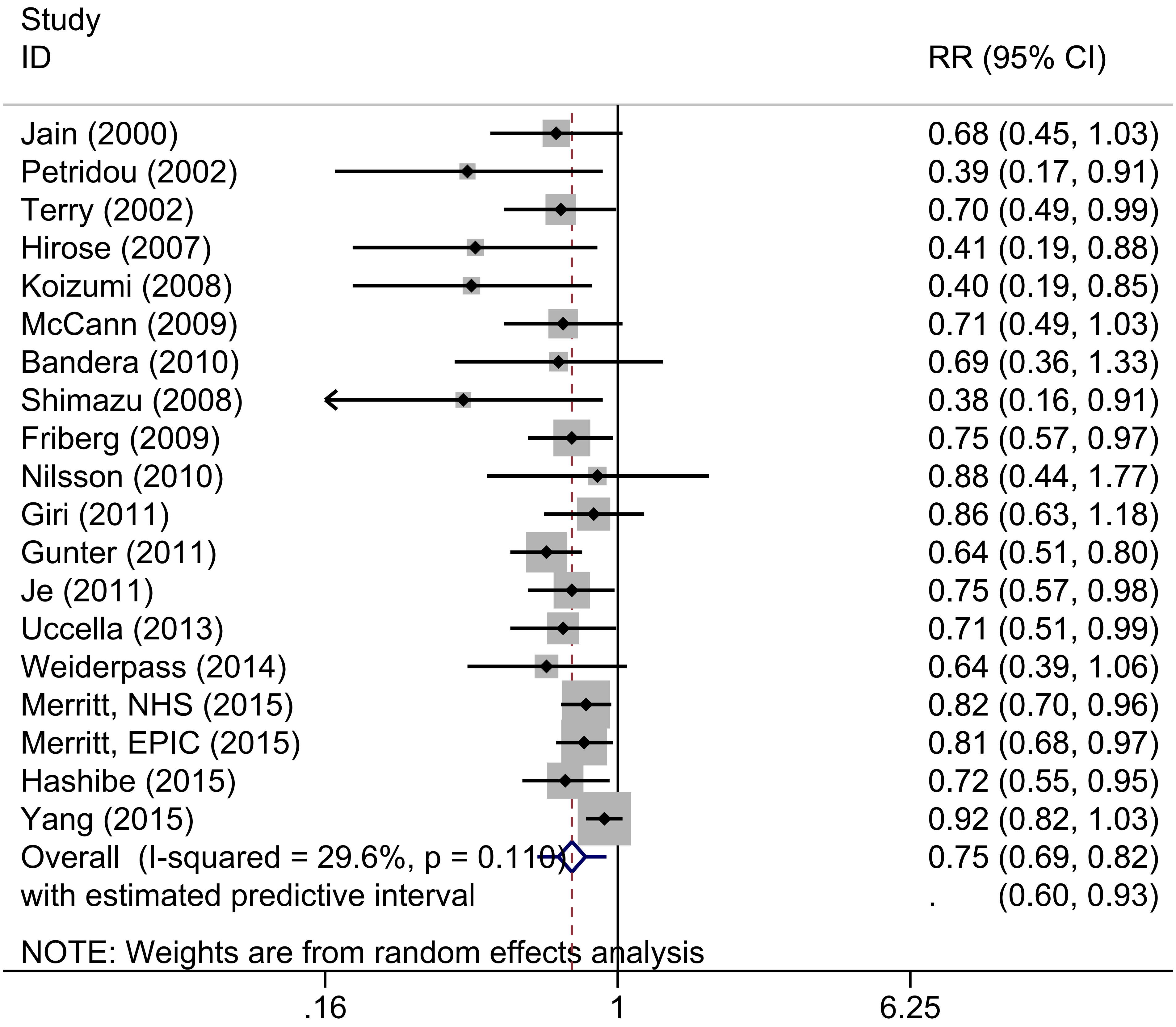

Supplementary figure 12, Endometrial cancer, Per 1 cup

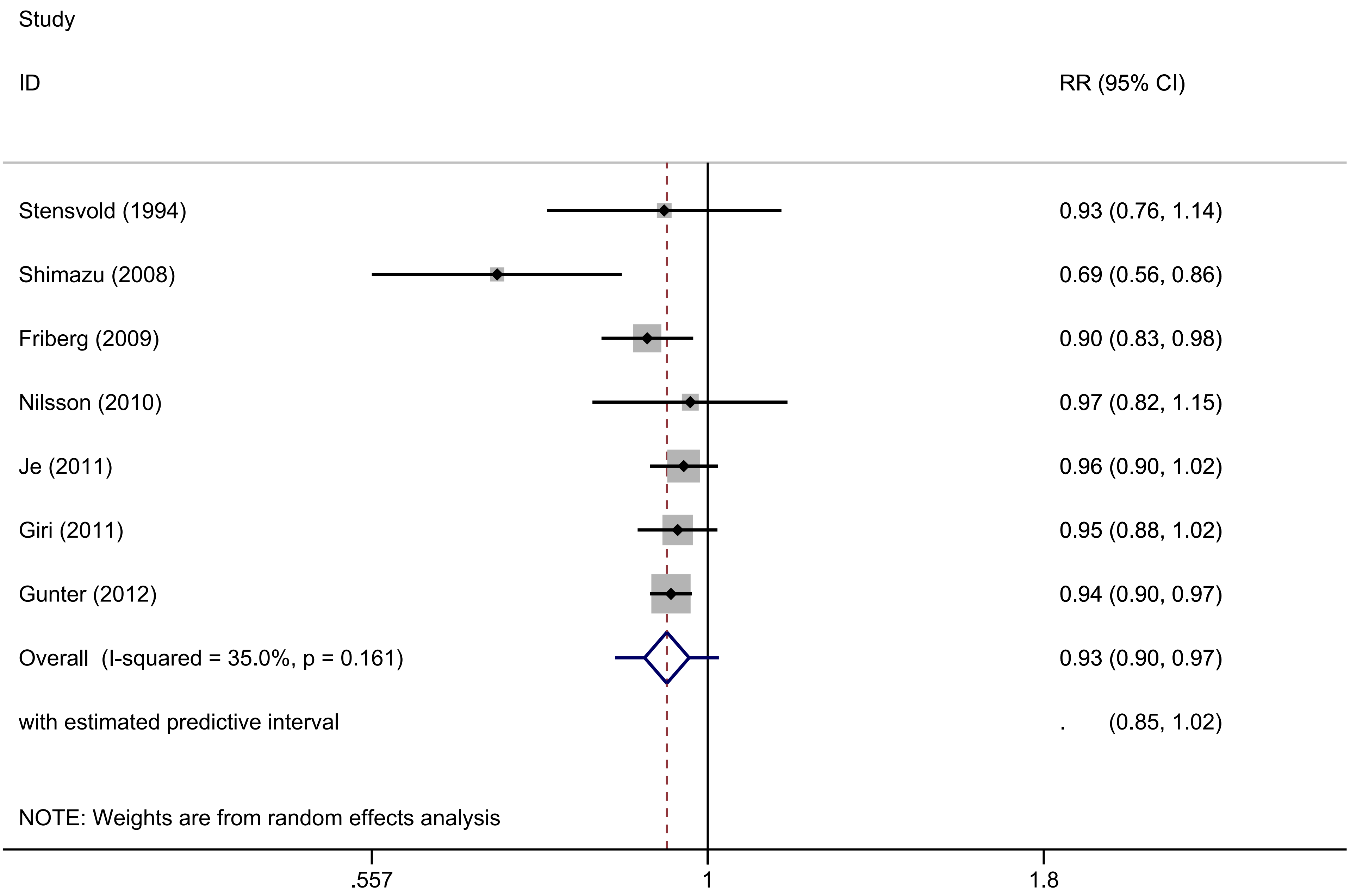

Supplementary figure 13, Esophageal cancer, H/L

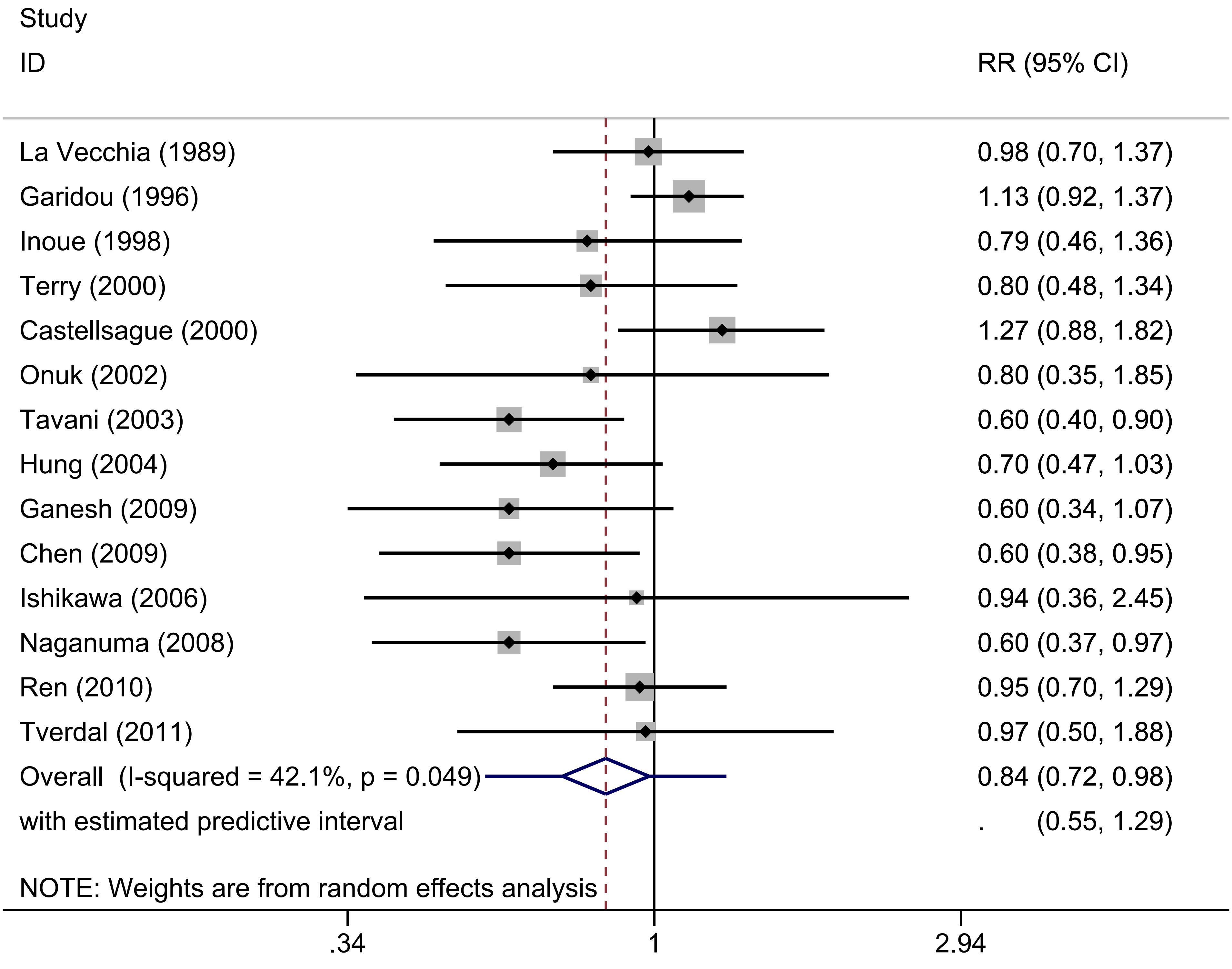

Supplementary figure 14, Gastric cancer, H/L

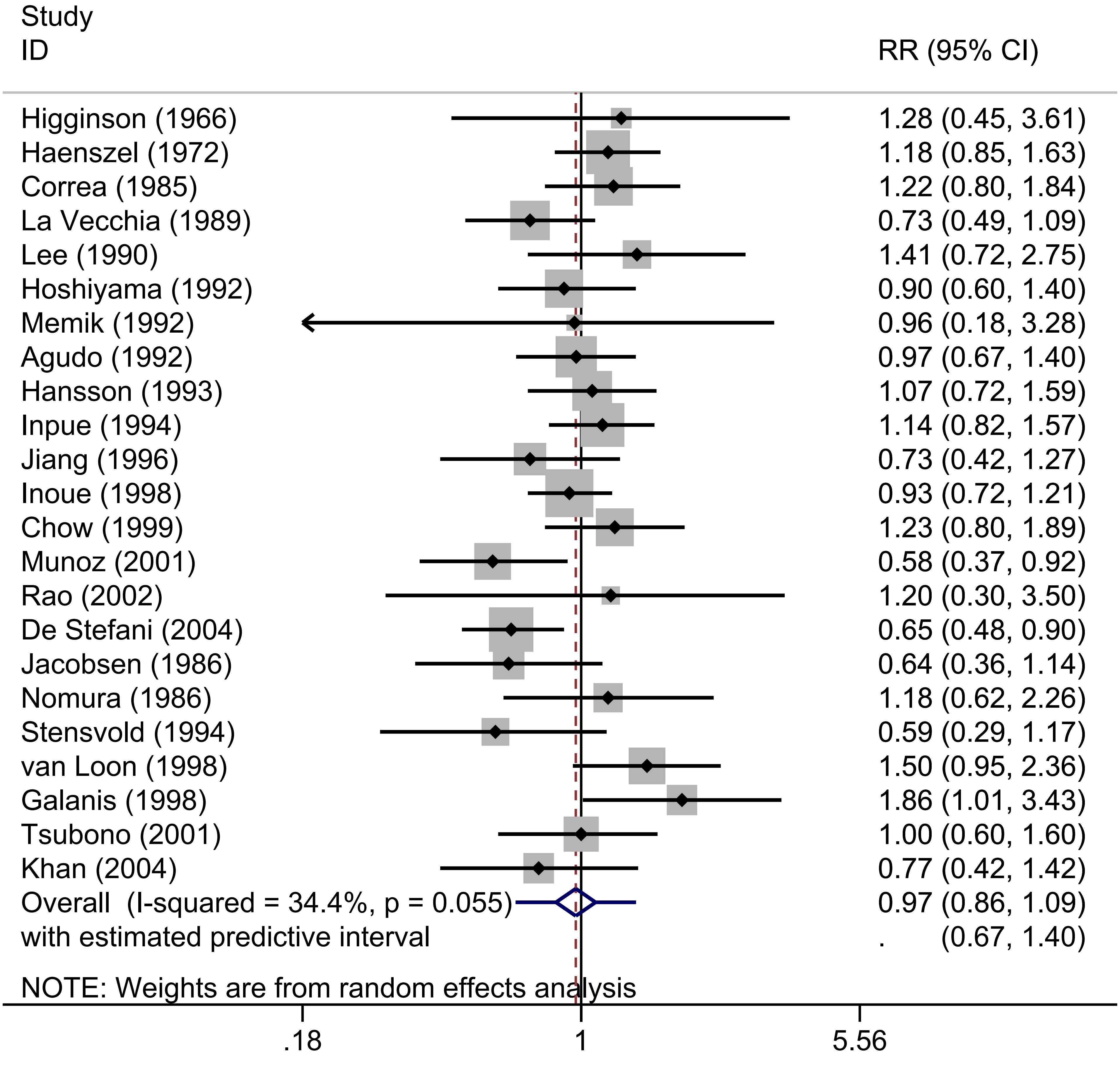

Supplementary figure 15, Gastric cancer, Per 1 cup

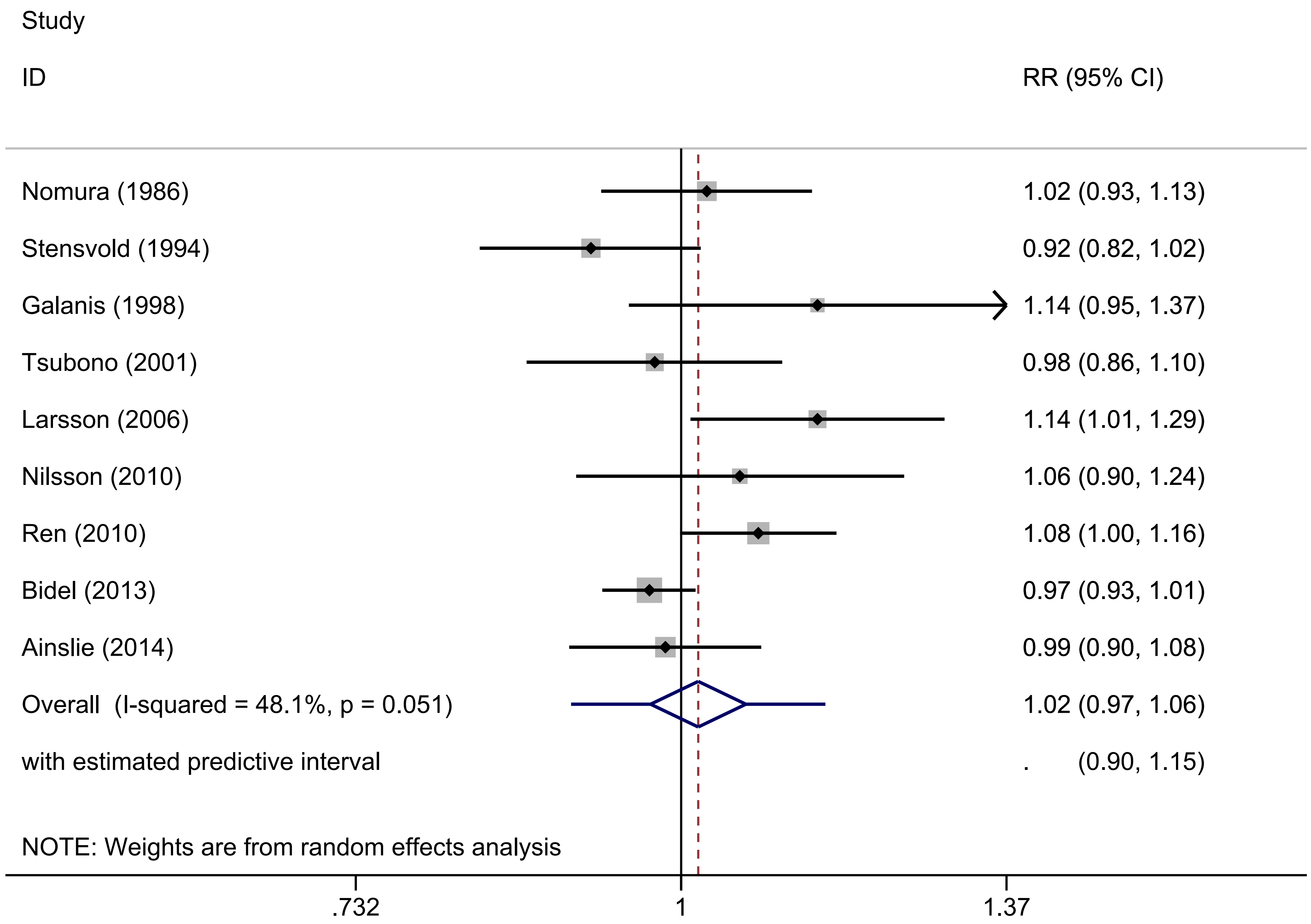

Supplementary figure 16, Glioma, H/L

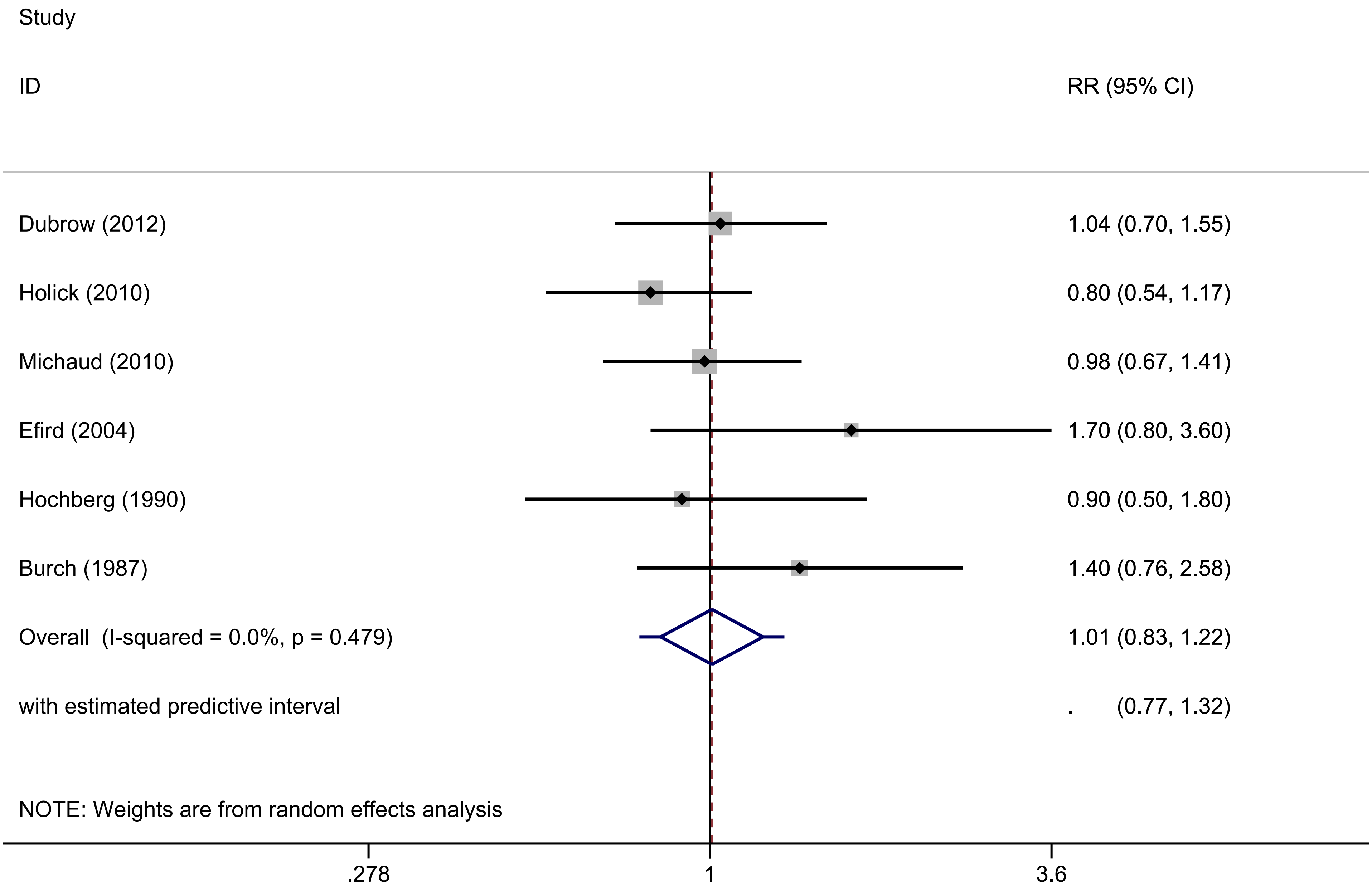

Supplementary figure 17, Glioma, Per 1 cup

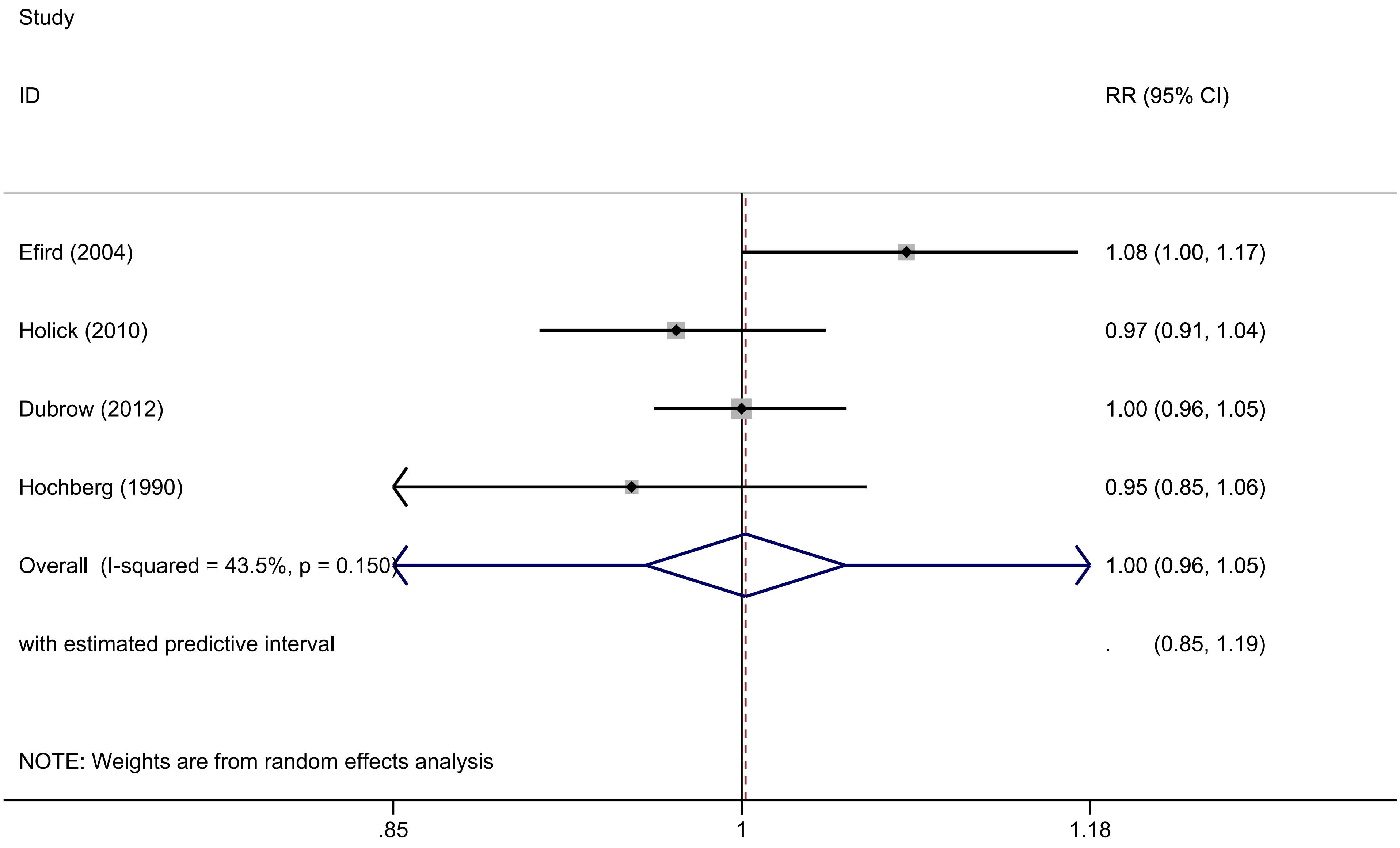

Supplementary figure 18, Kidney cancer, H/L

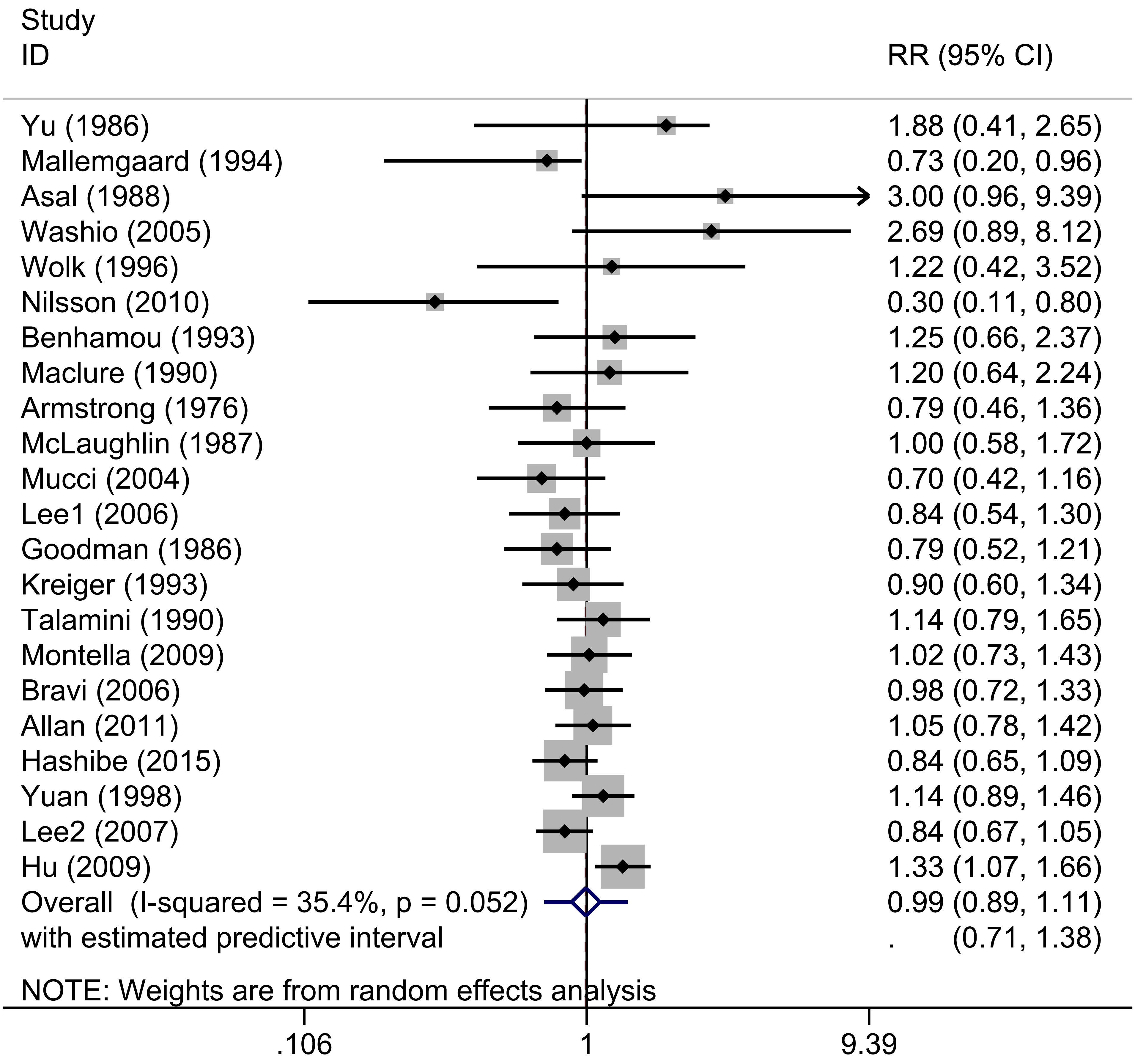

Supplementary figure 19, Laryngeal cancer, H/L

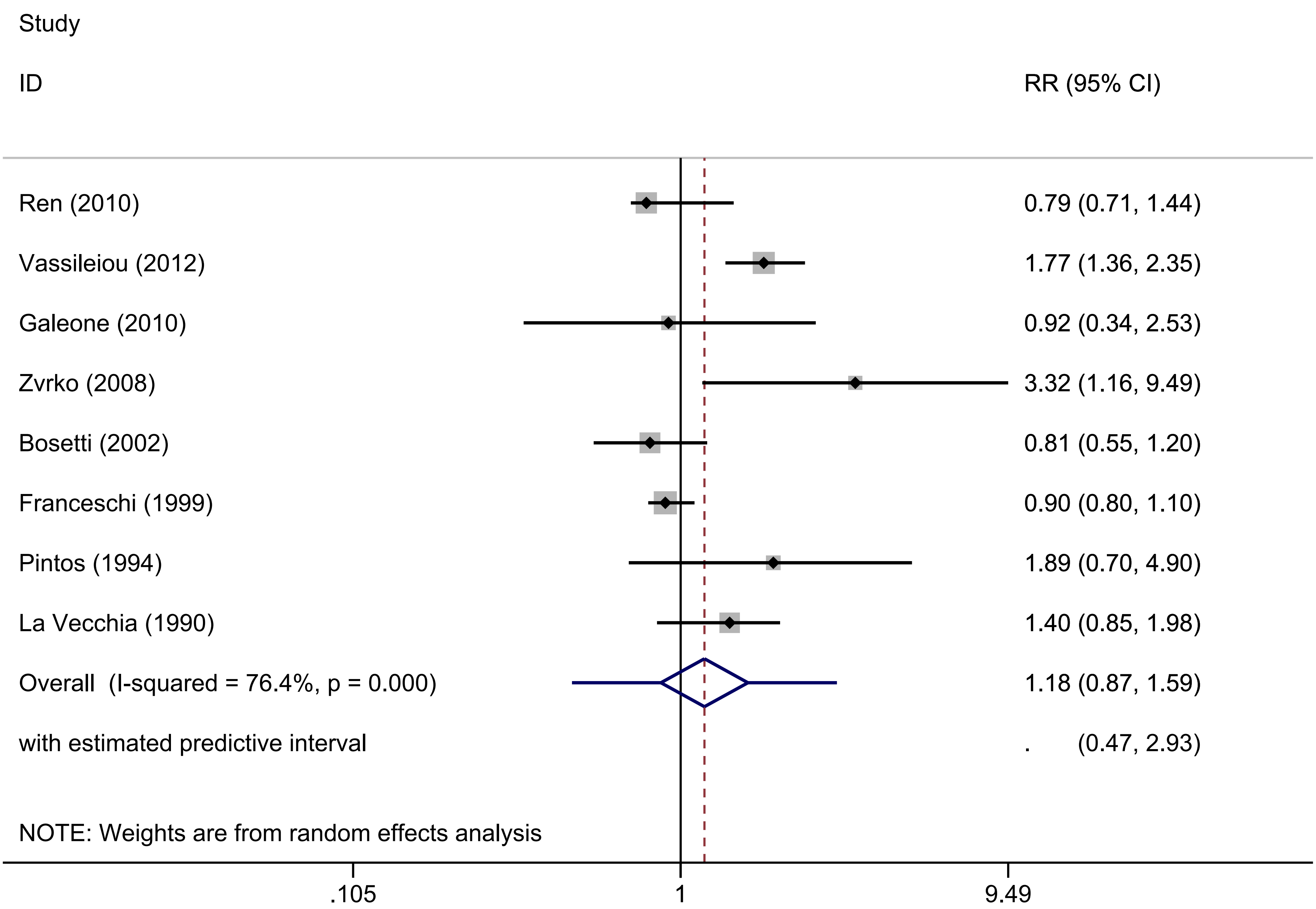

Supplementary figure 20, Liver cancer, H/L

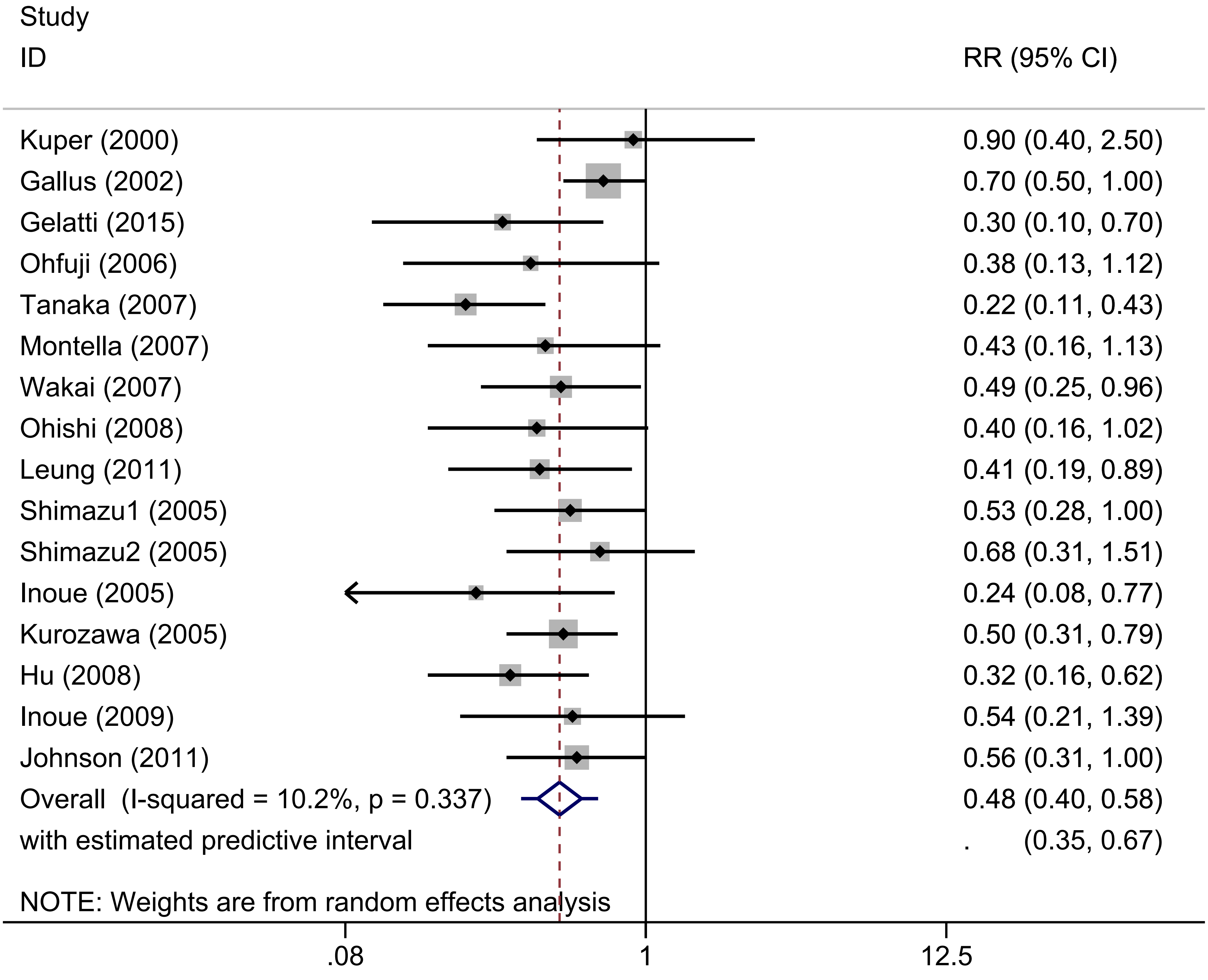

Supplementary figure 21, Liver cancer, Per 1 cup

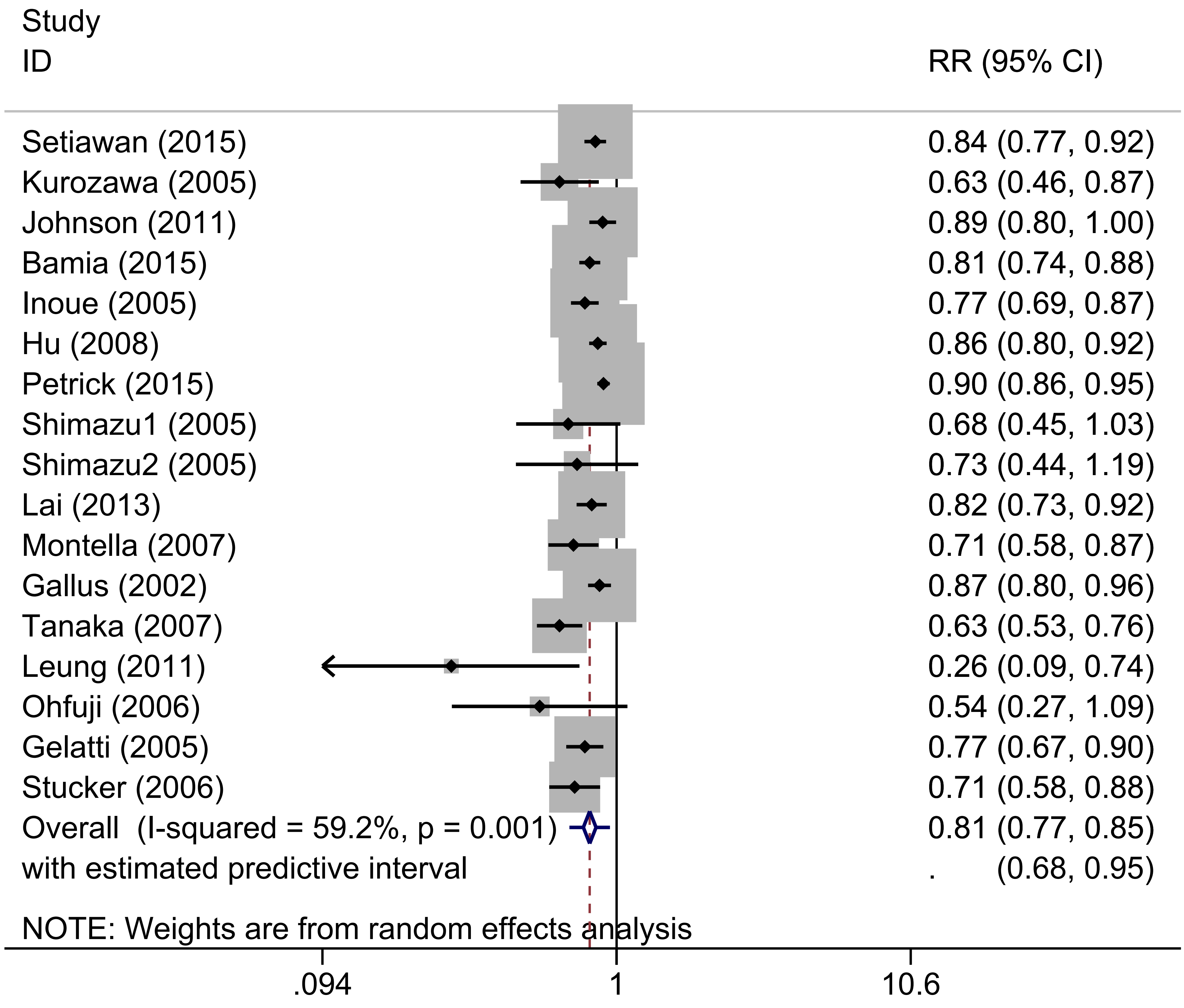

Supplementary figure 22, Lung cancer, H/L

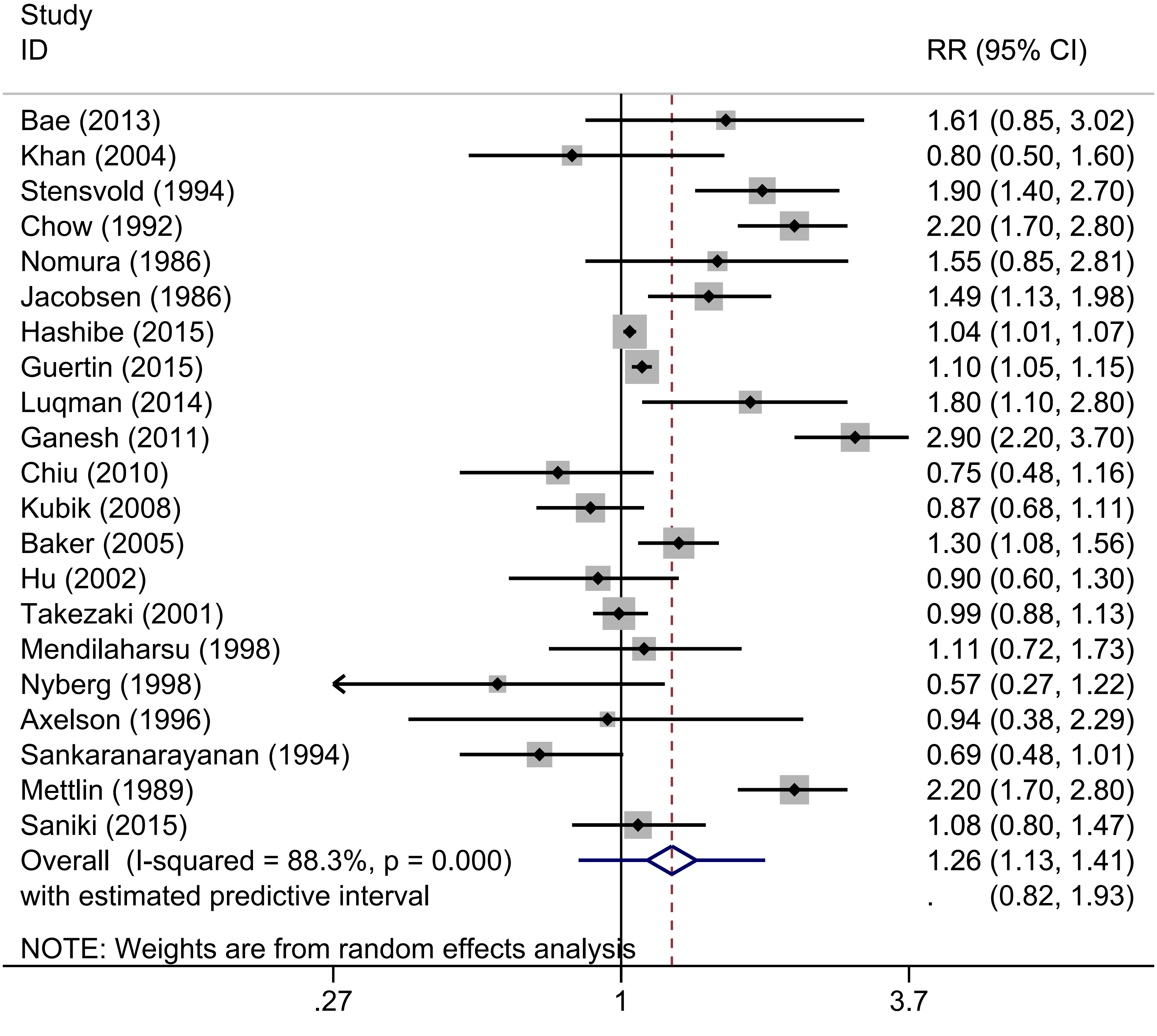

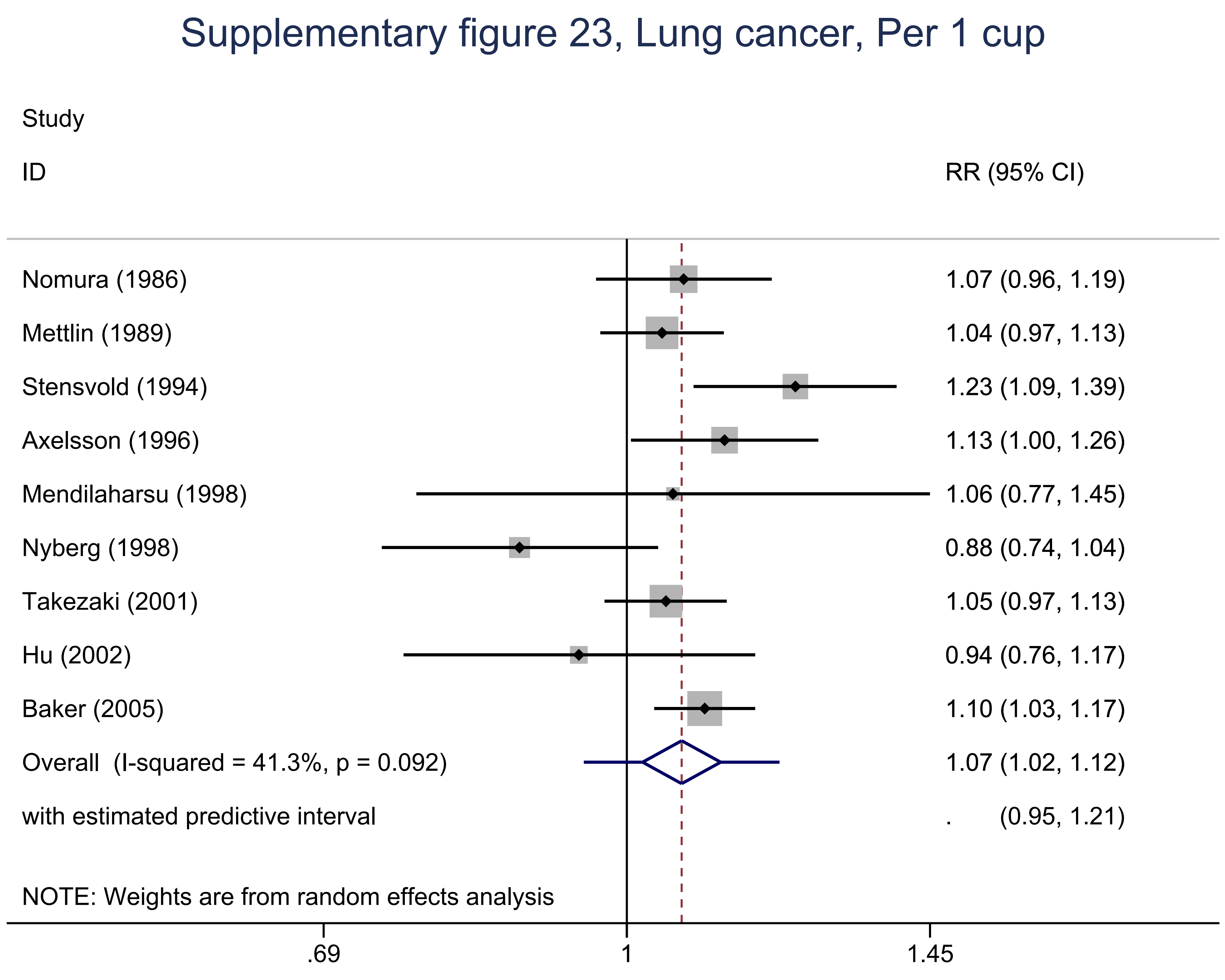
Supplementary figure 23, Lung cancer, Per 1 cup
Study
ID
RR (95% CI)
Nomura (1986)
1.07 (0.96, 1.19)
Mettlin (1989)
1.04 (0.97, 1.13)
Stensvold (1994)
1.23 (1.09, 1.39)
Axelsson (1996)
1.13 (1.00, 1.26)
Mendilaharsu (1998)
1.06 (0.77, 1.45)
Nyberg (1998)
0.88 (0.74, 1.04)
Takezaki (2001)
1.05 (0.97, 1.13)
Hu (2002)
0.94 (0.76, 1.17)
Baker (2005)
1.10 (1.03, 1.17)
Overall (I-squared = 41.3%, p = 0.092)
1.07 (1.02, 1.12)
with estimated predictive interval
. (0.95, 1.21)
NOTE: Weights are from random effects analysis
.69
1
1.45

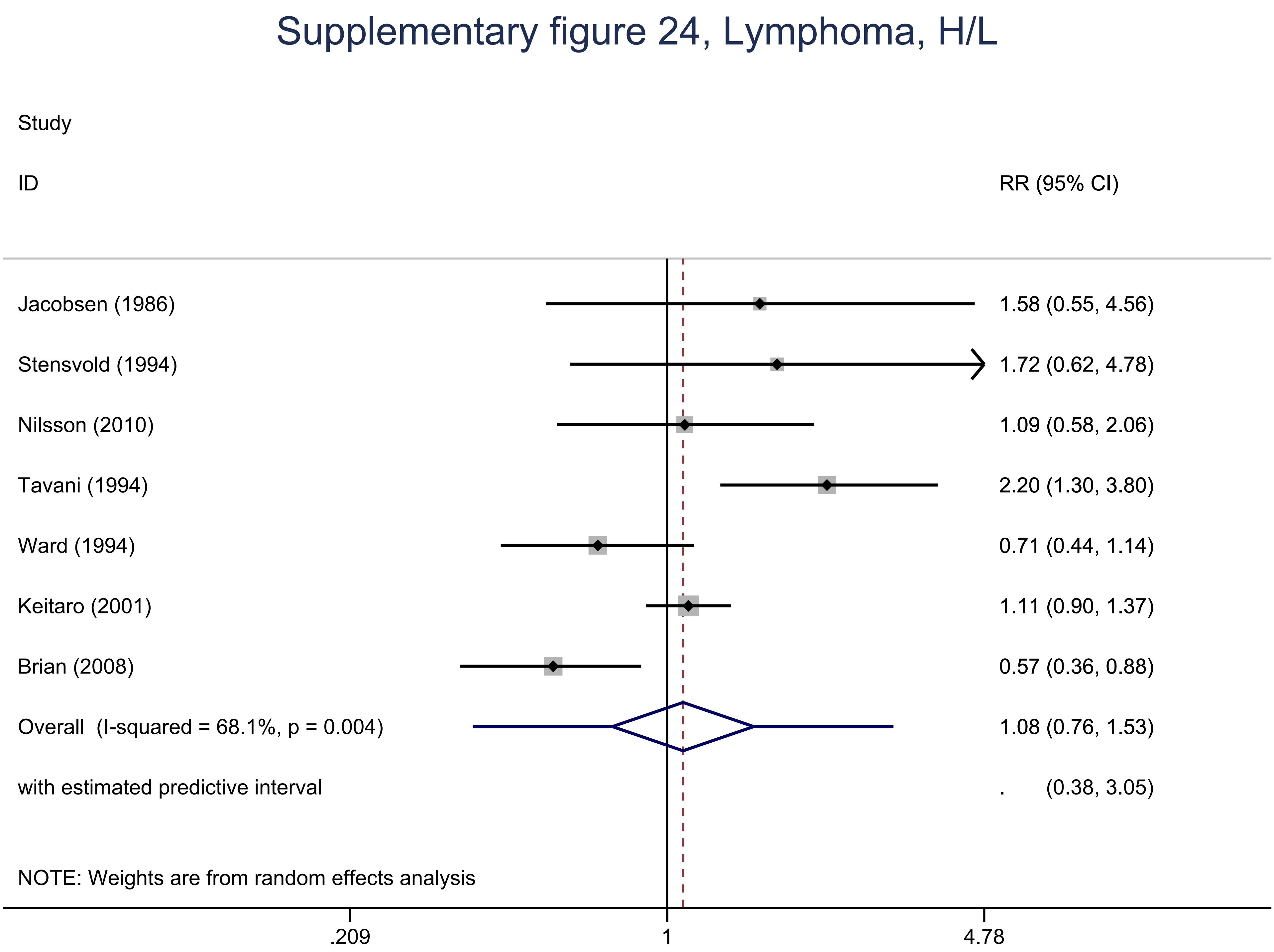
Supplementary figure 24, Lymphoma, H/L
Study
ID
RR (95% CI)
Jacobsen (1986)
1.58 (0.55, 4.56)
Stensvold (1994)
1.72 (0.62, 4.78)
Nilsson (2010)
1.09 (0.58, 2.06)
Tavani (1994)
2.20 (1.30, 3.80)
Ward (1994)
0.71 (0.44, 1.14)
Keitaro (2001)
1.11 (0.90, 1.37)
Brian (2008)
0.57 (0.36, 0.88)
Overall (I-squared = 68.1%, p = 0.004)
1.08 (0.76, 1.53)
with estimated predictive interval
. (0.38, 3.05)
NOTE: Weights are from random effects analysis
.209
1
4.78

Supplementary figure 25, Melanoma, H/L

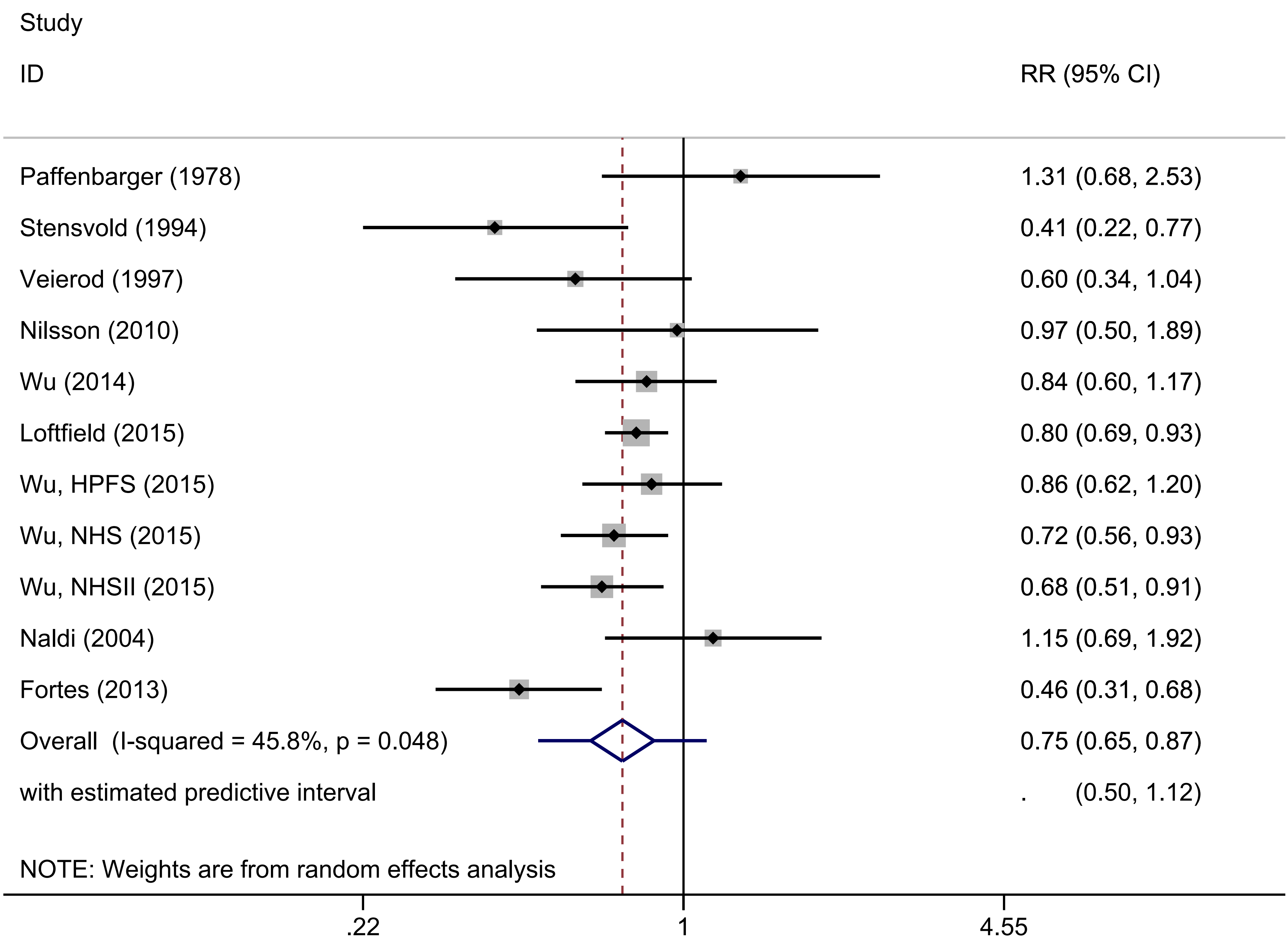

Supplementary figure 26, Nonmelanoma, H/L

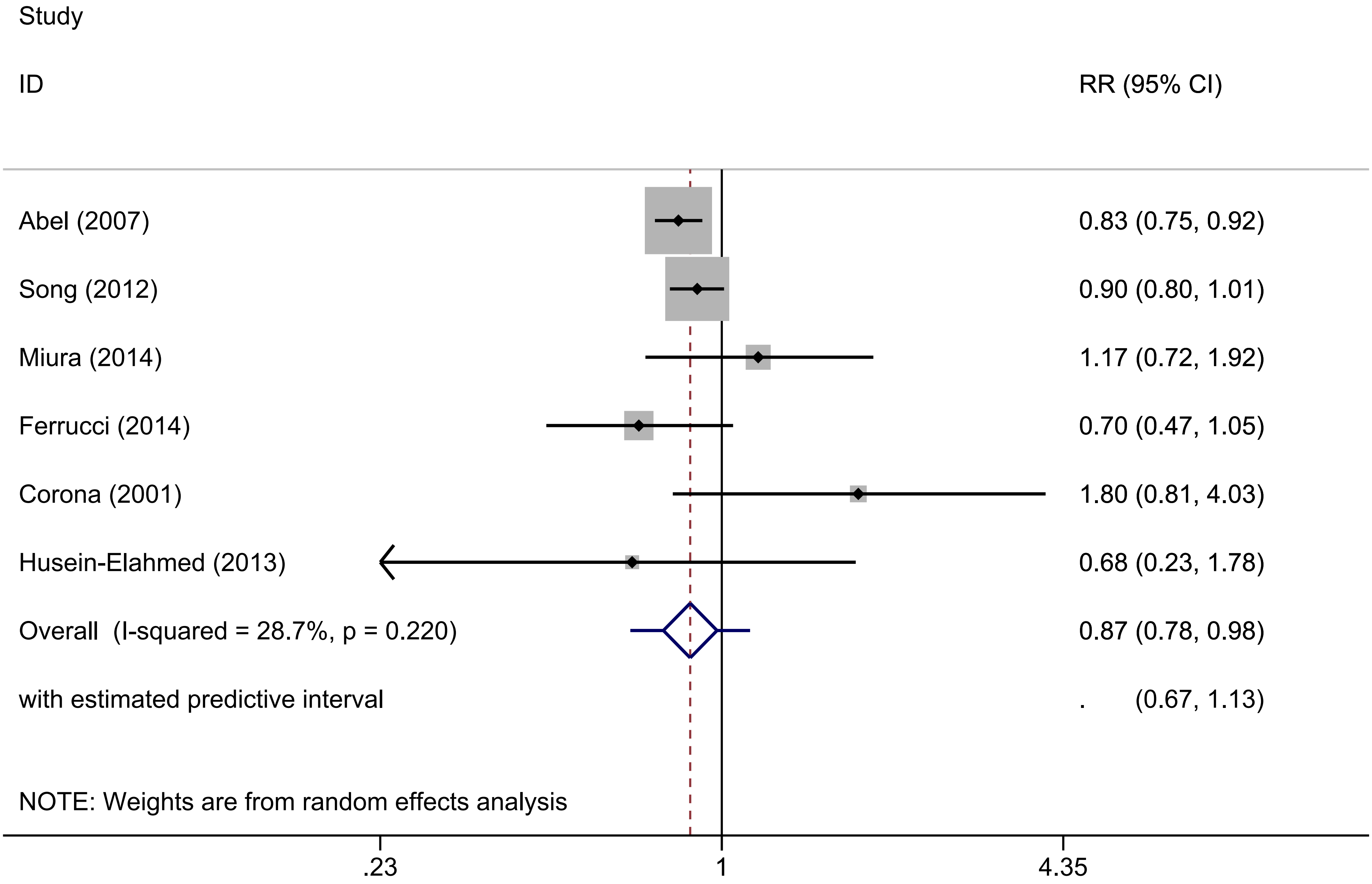

Supplementary figure 27, Oral cancer, H/L

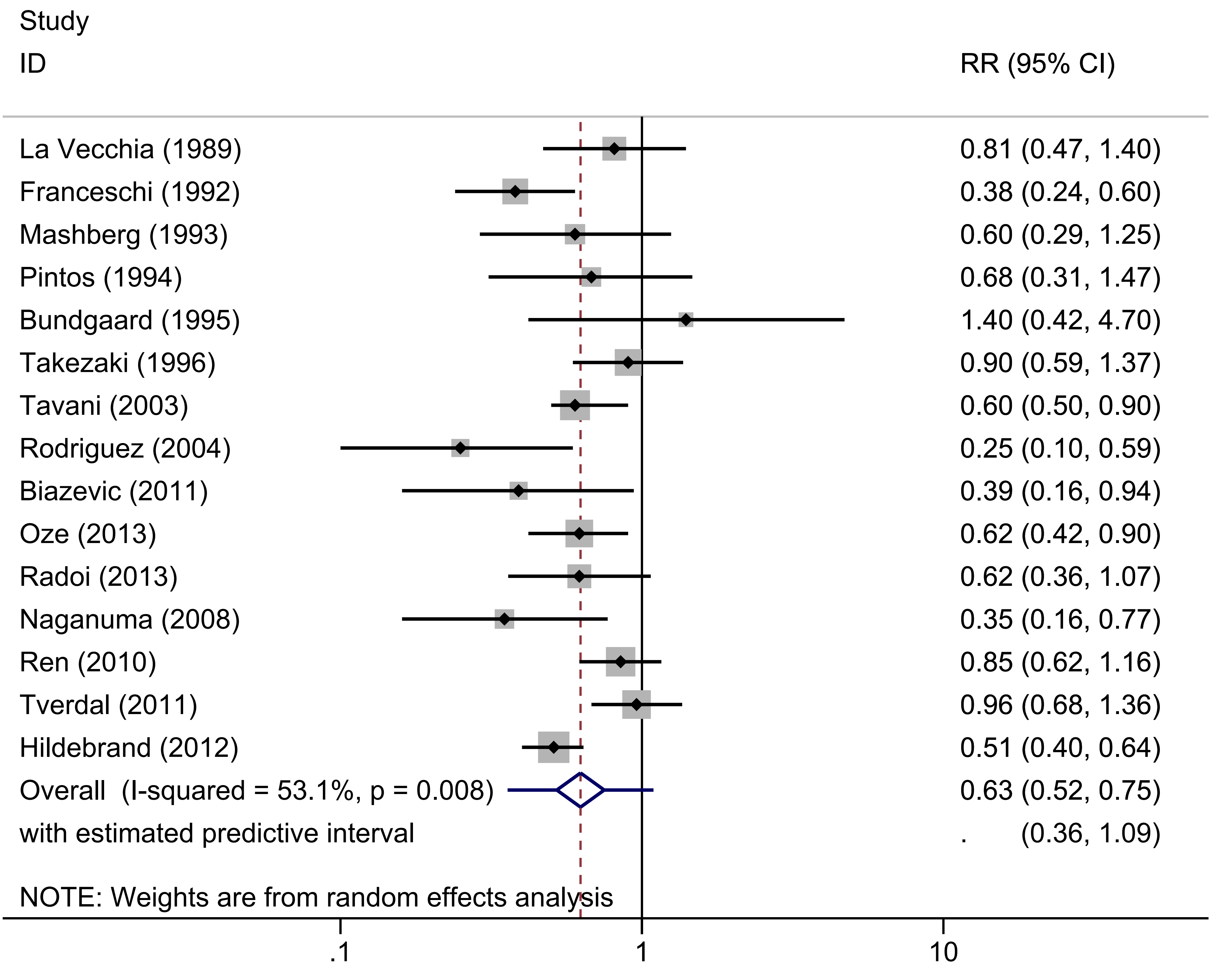

Supplementary figure 28, Oral/Pharyngeal cancer, H/L

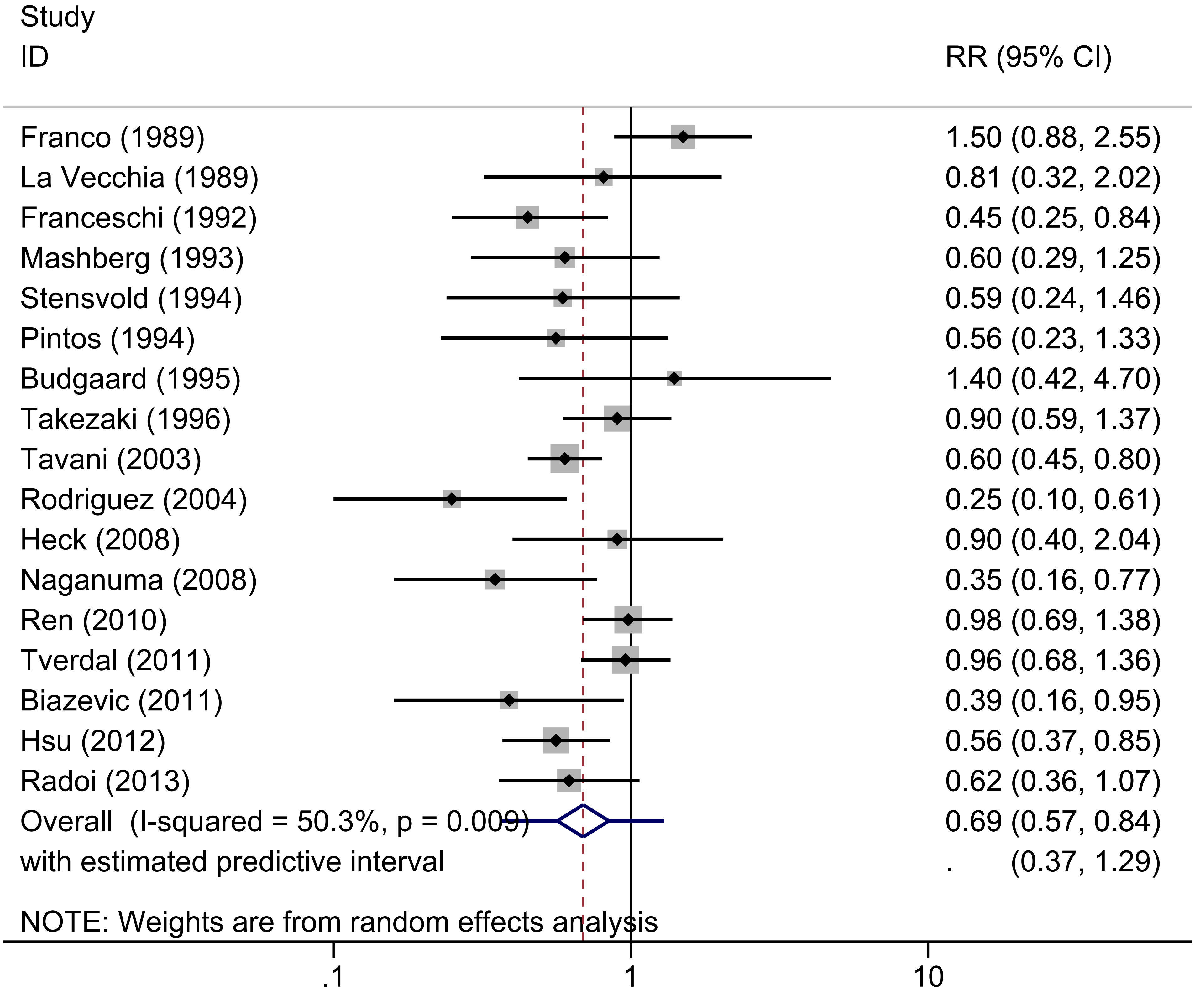

Supplementary figure 29, Ovarian cancer, H/L

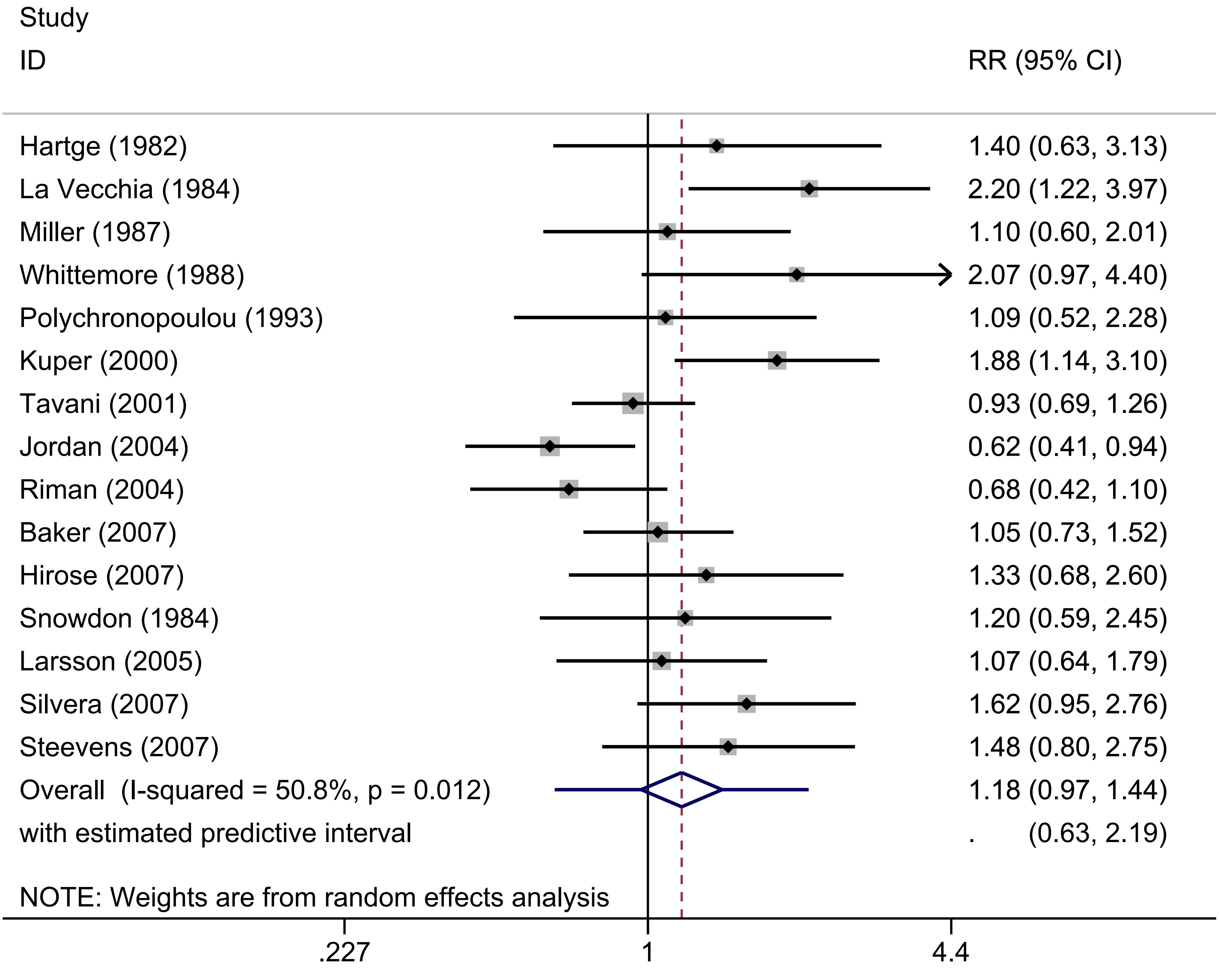

Supplementary figure 30, Pancreatic cancer, H/L

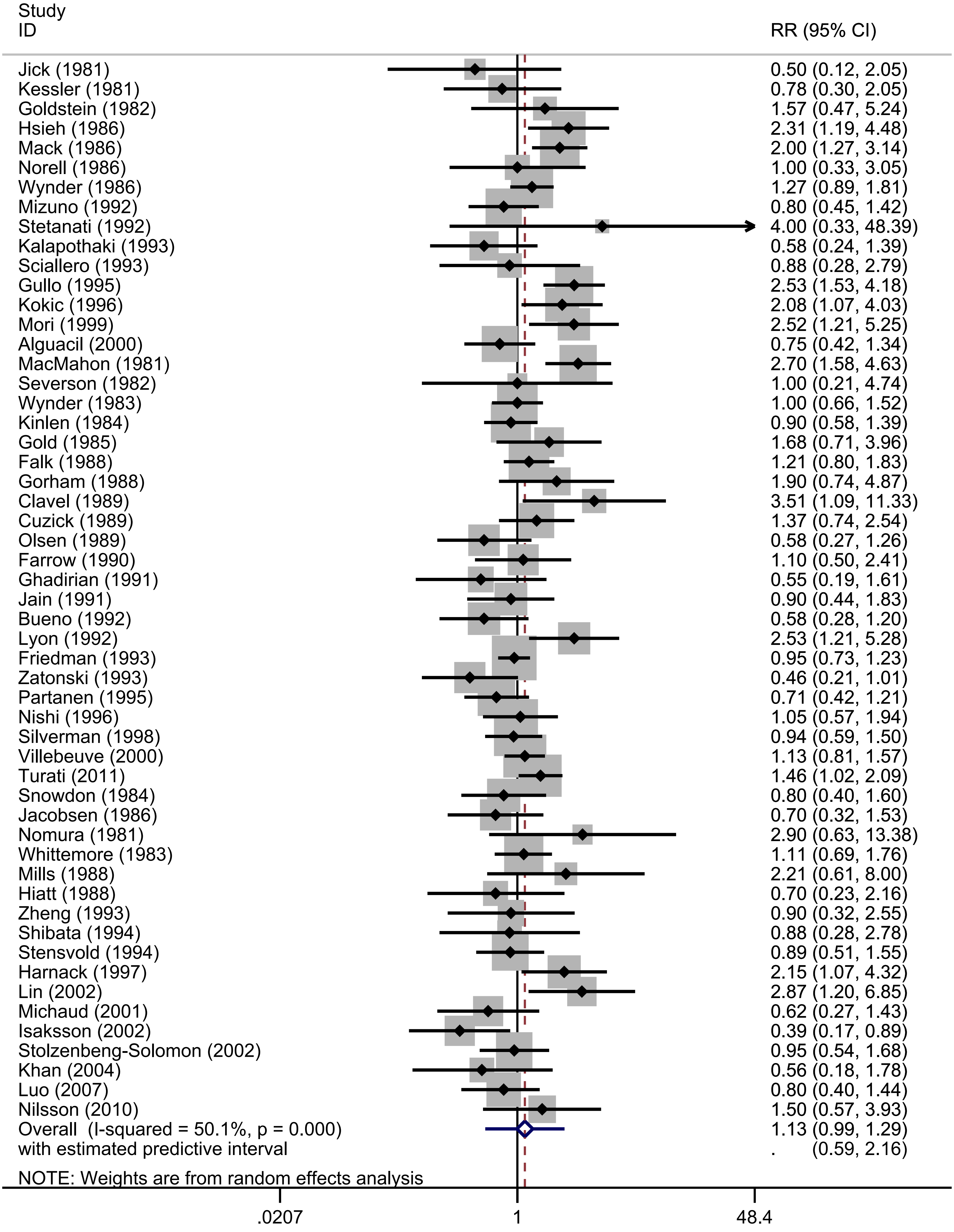

Supplementary figure 31, Pancreatic cancer, Per 1 cup

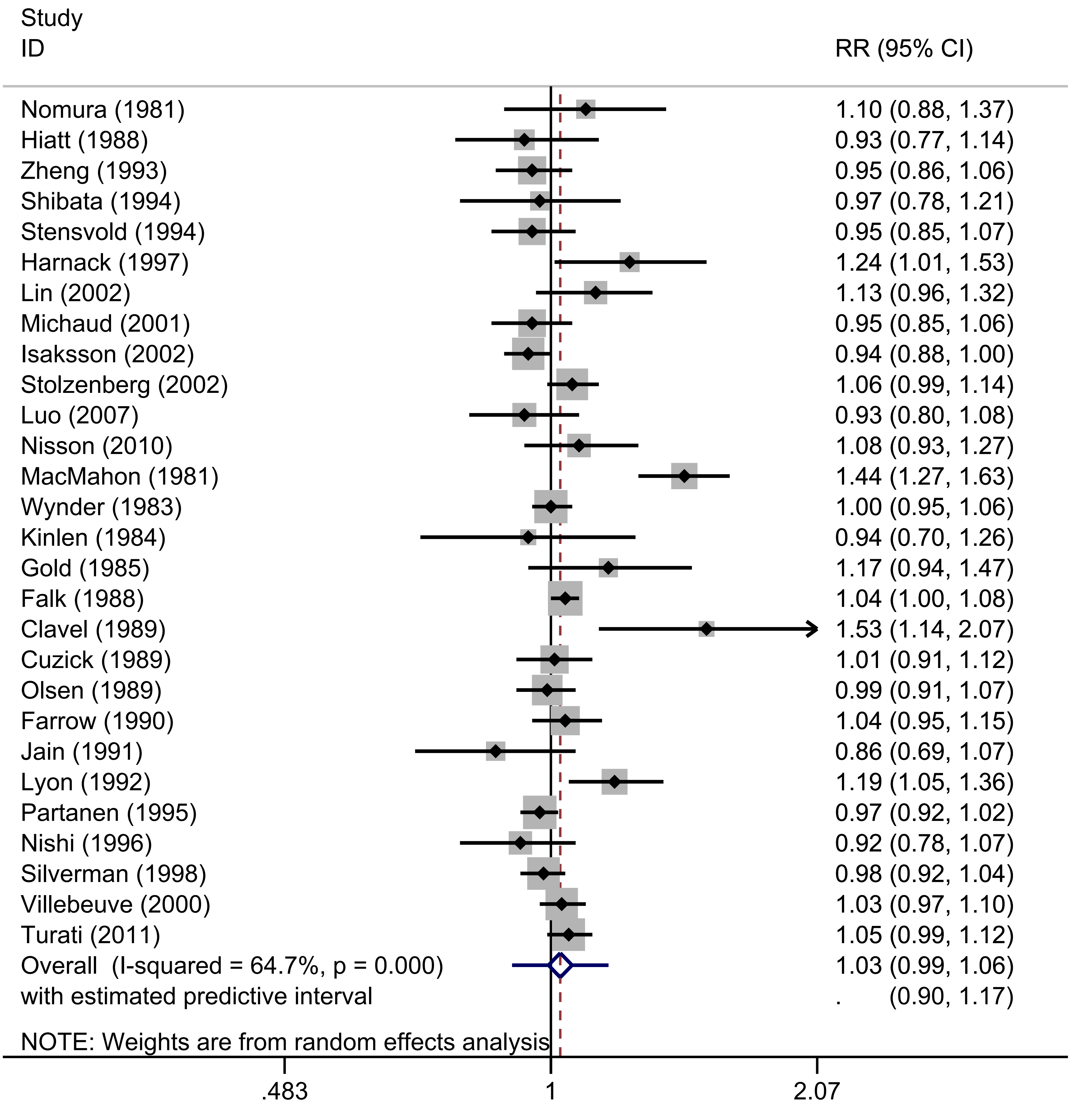

Supplementary figure 32, Prostate cancer, H/L

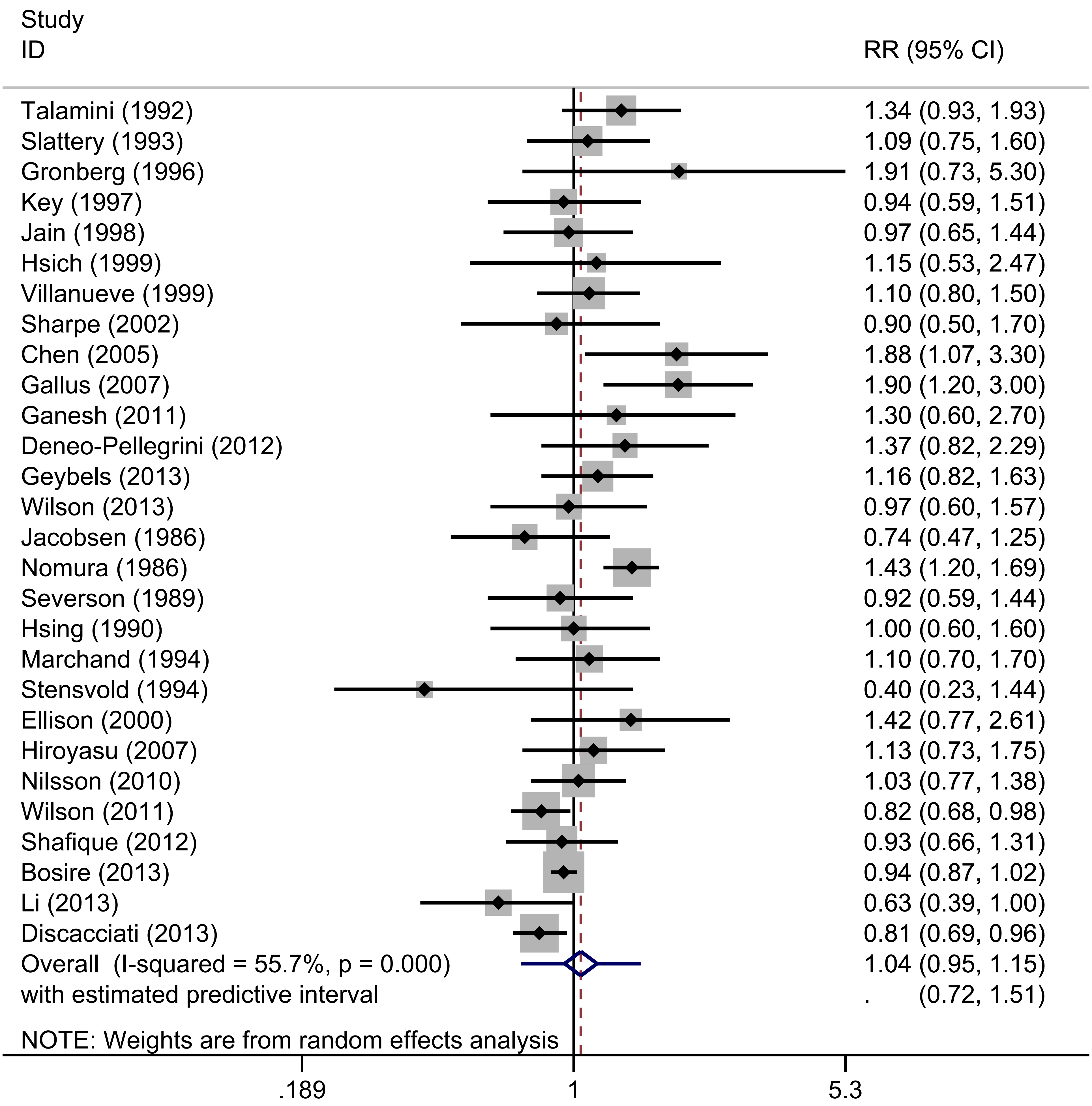

Supplementary figure 33, Rectal cancer, H/L

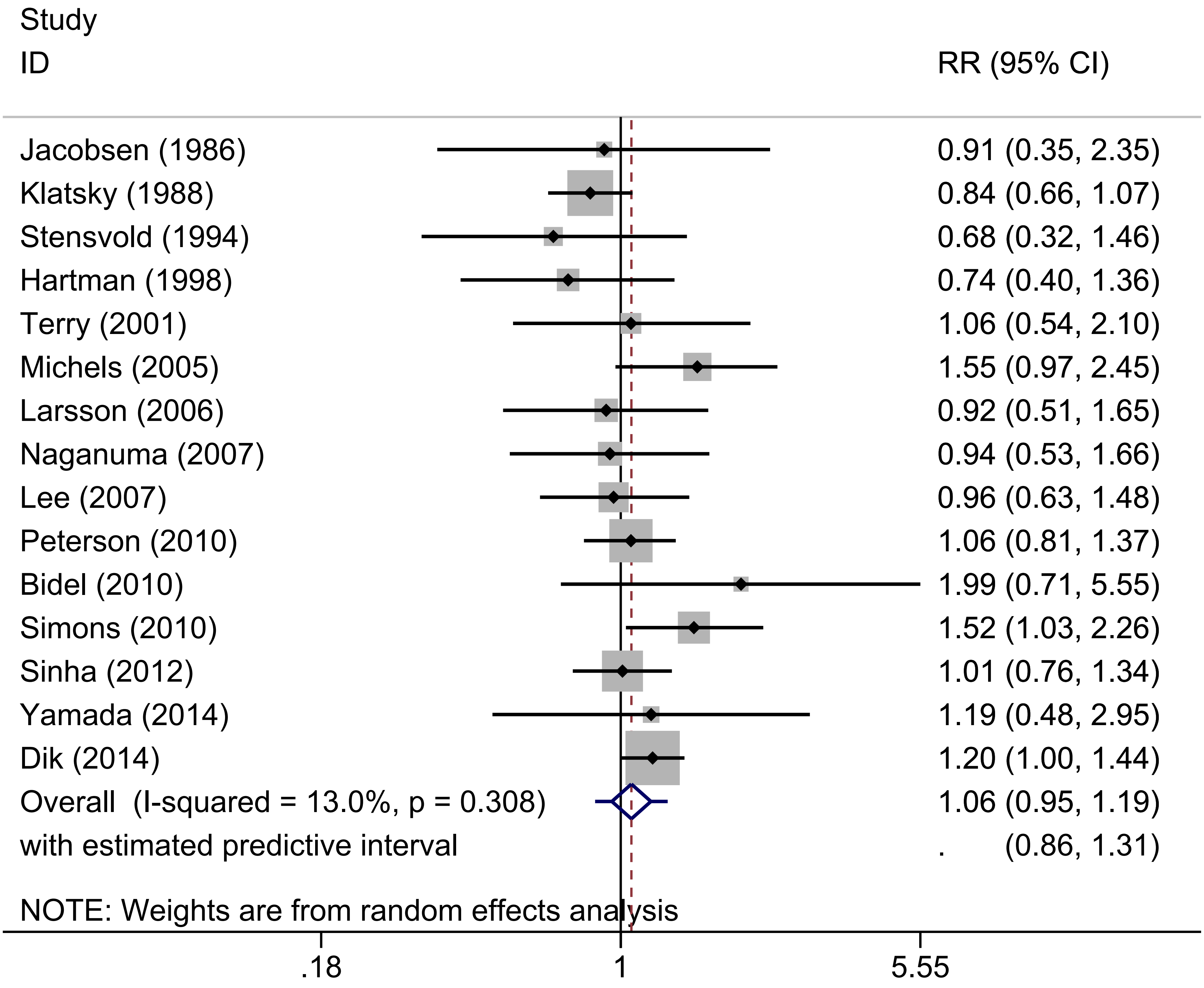

Supplementary figure 34, Rectal cancer, Per 1 cup

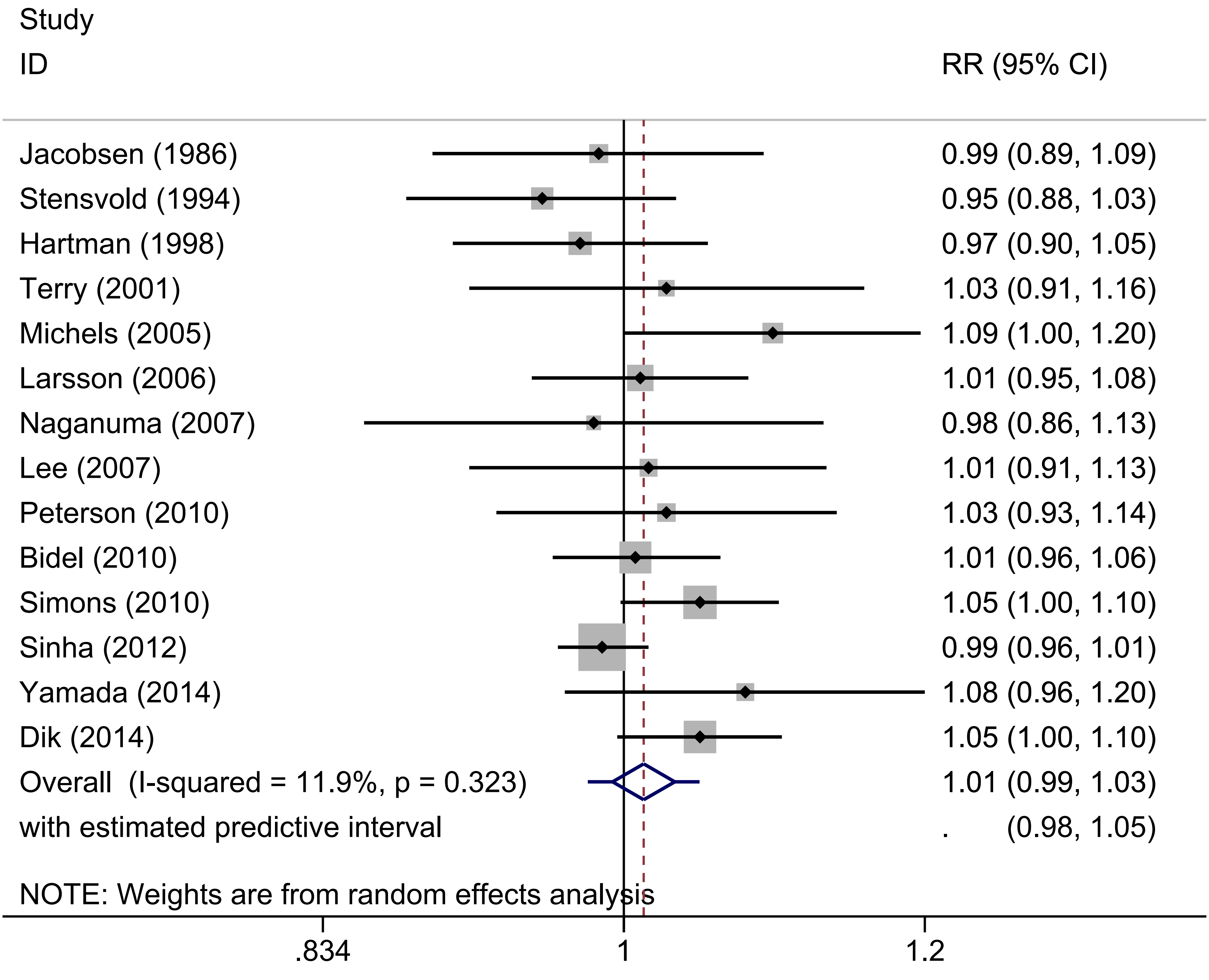

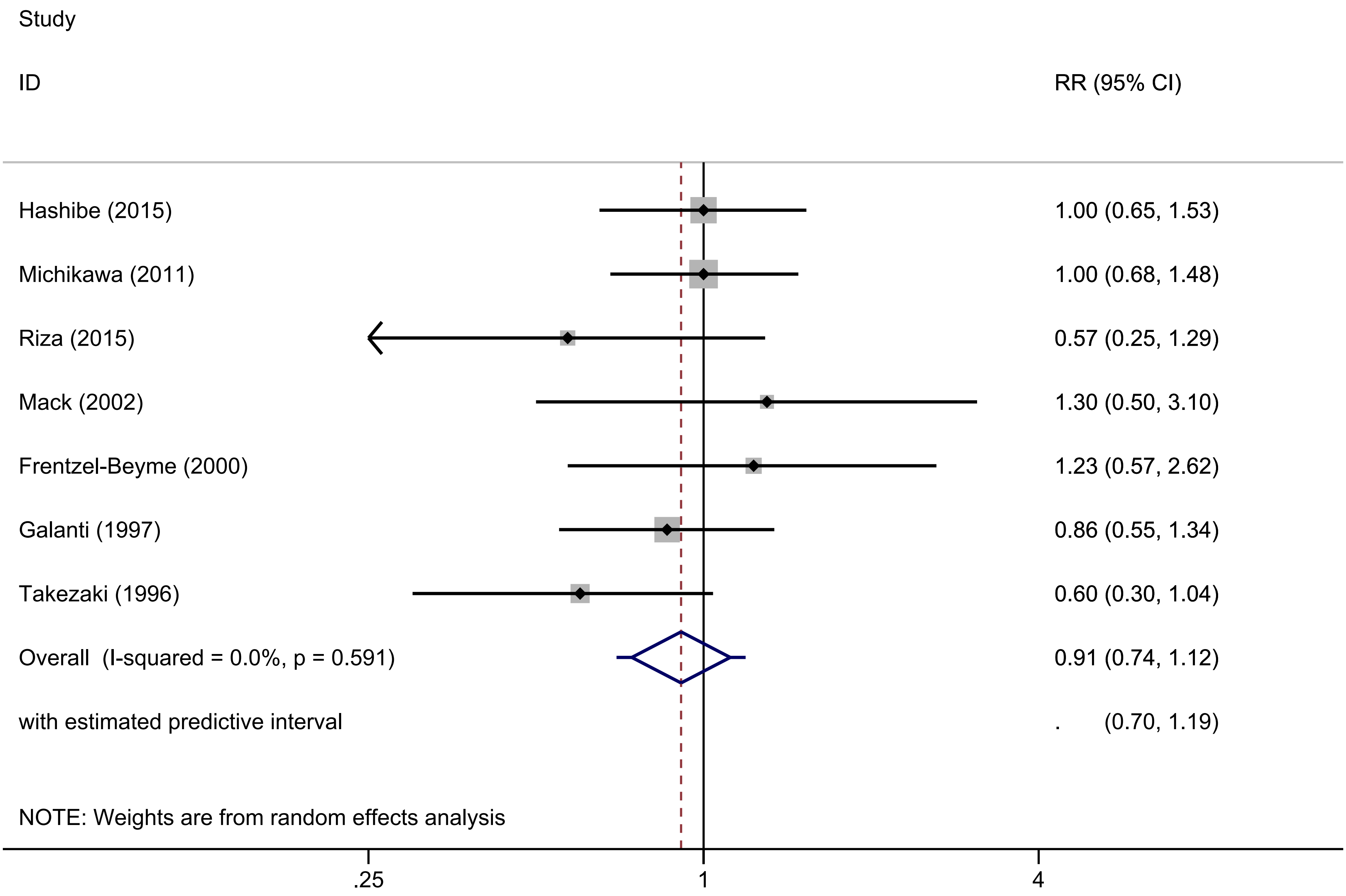

Supplementary figure 35, Thyroid cancer, H/L

Supplementary figure 36, Biliary tract cancer, H/L

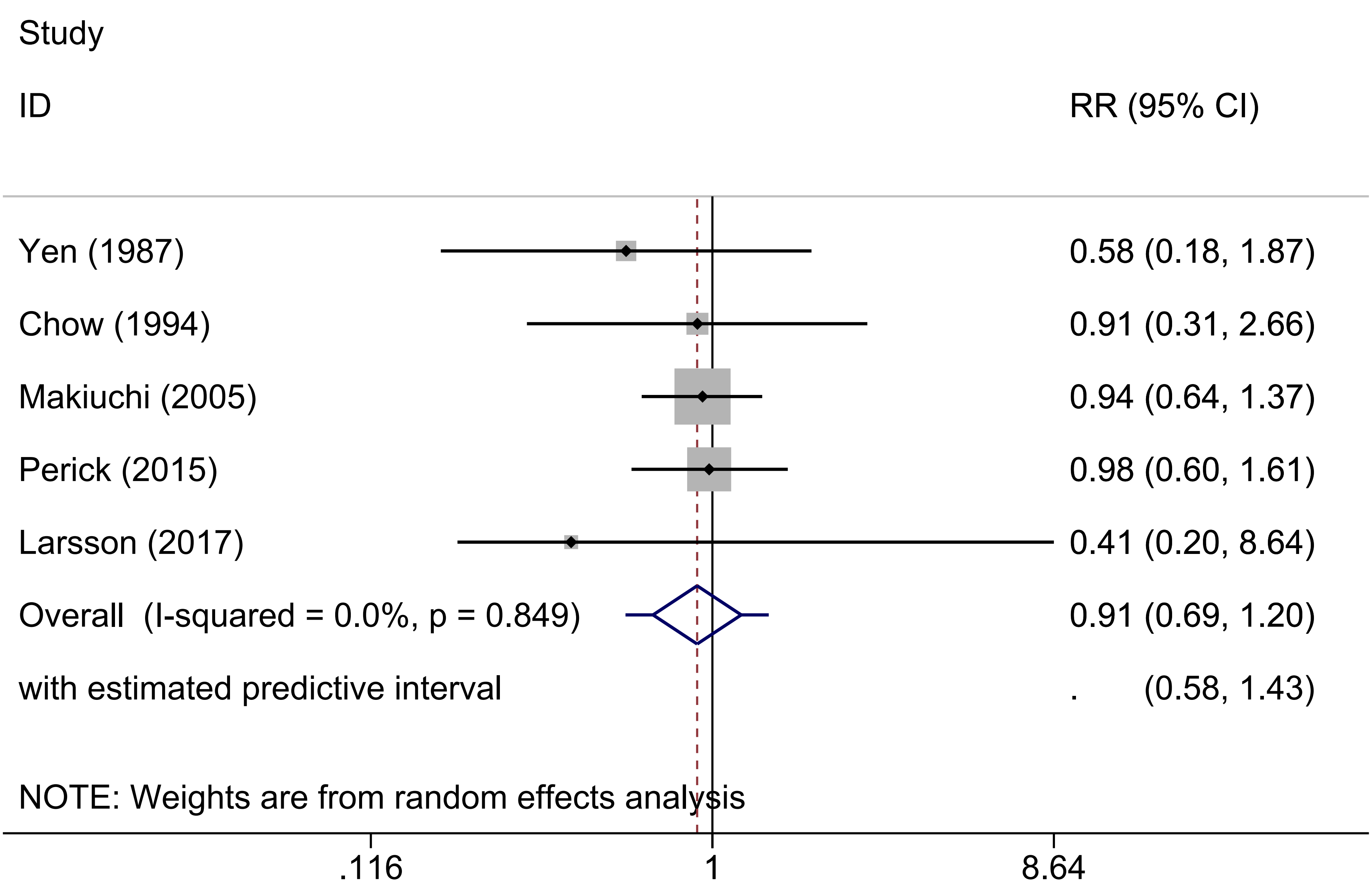

Supplementary figure 37, Bladder cancer, H/L, only cohort studies

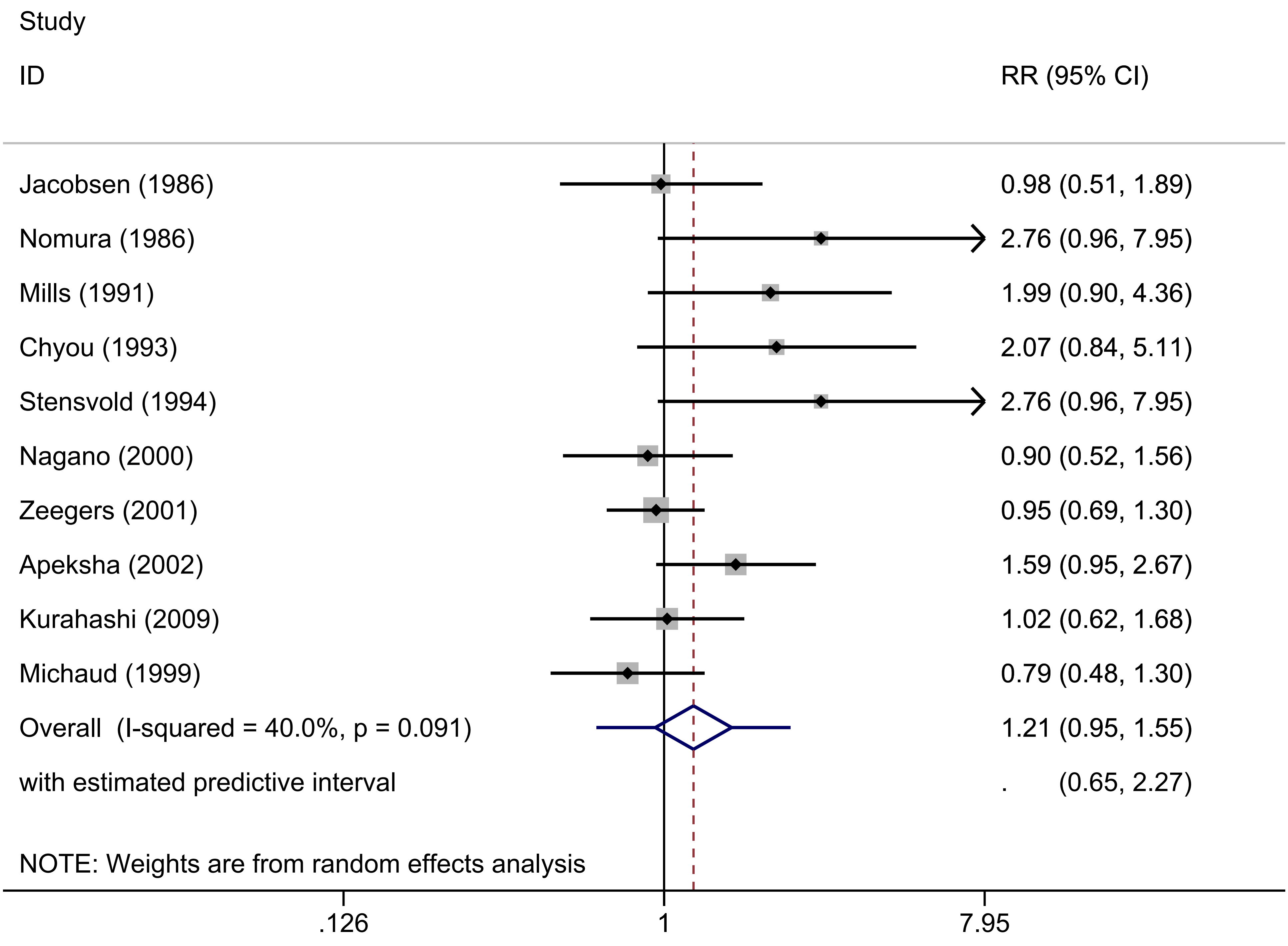

Supplementary figure 38, Breast cancer, H/L, only cohort studies

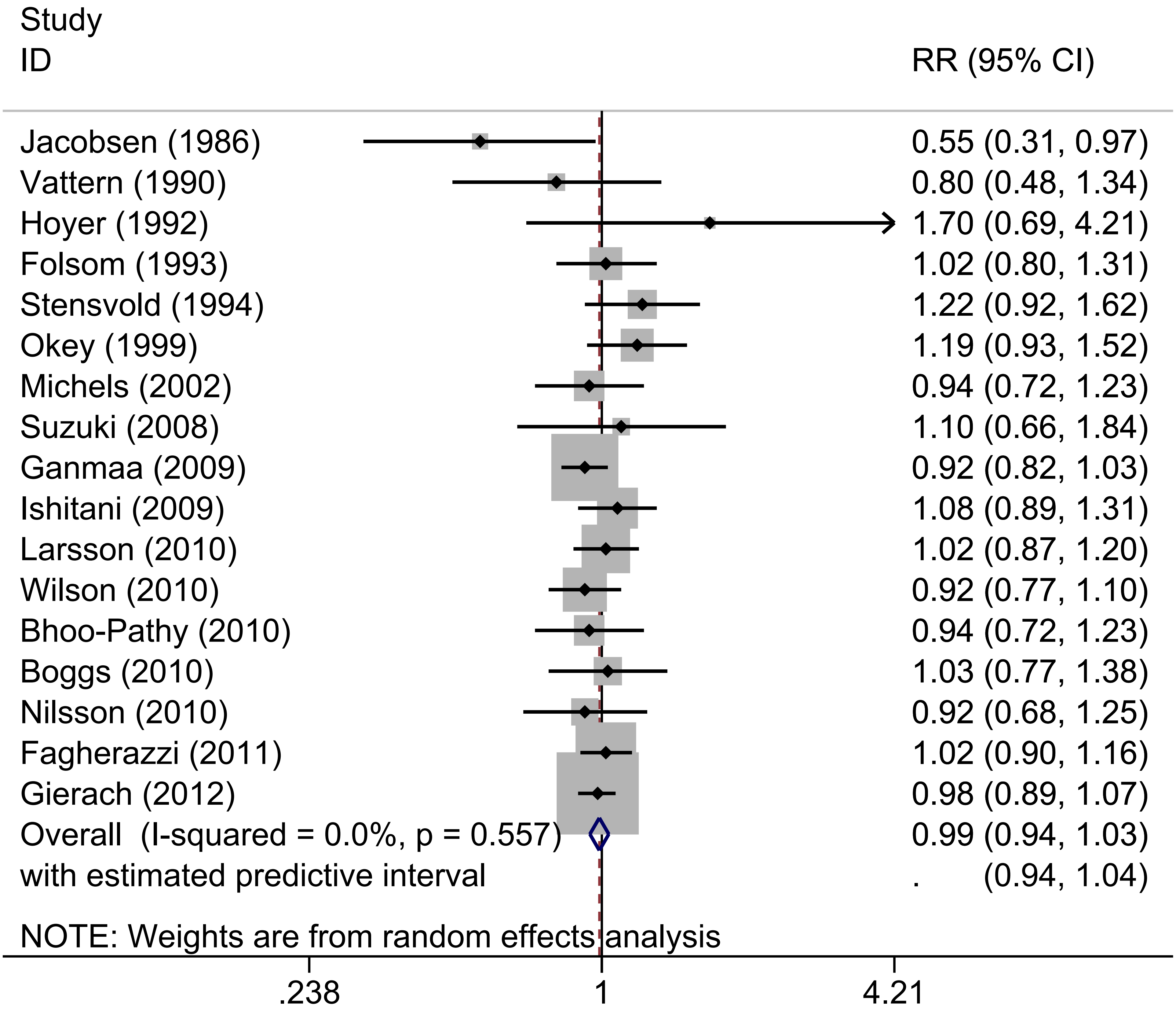

Supplementary figure 39, Breast cancer, Per 1 cup, only cohort studies

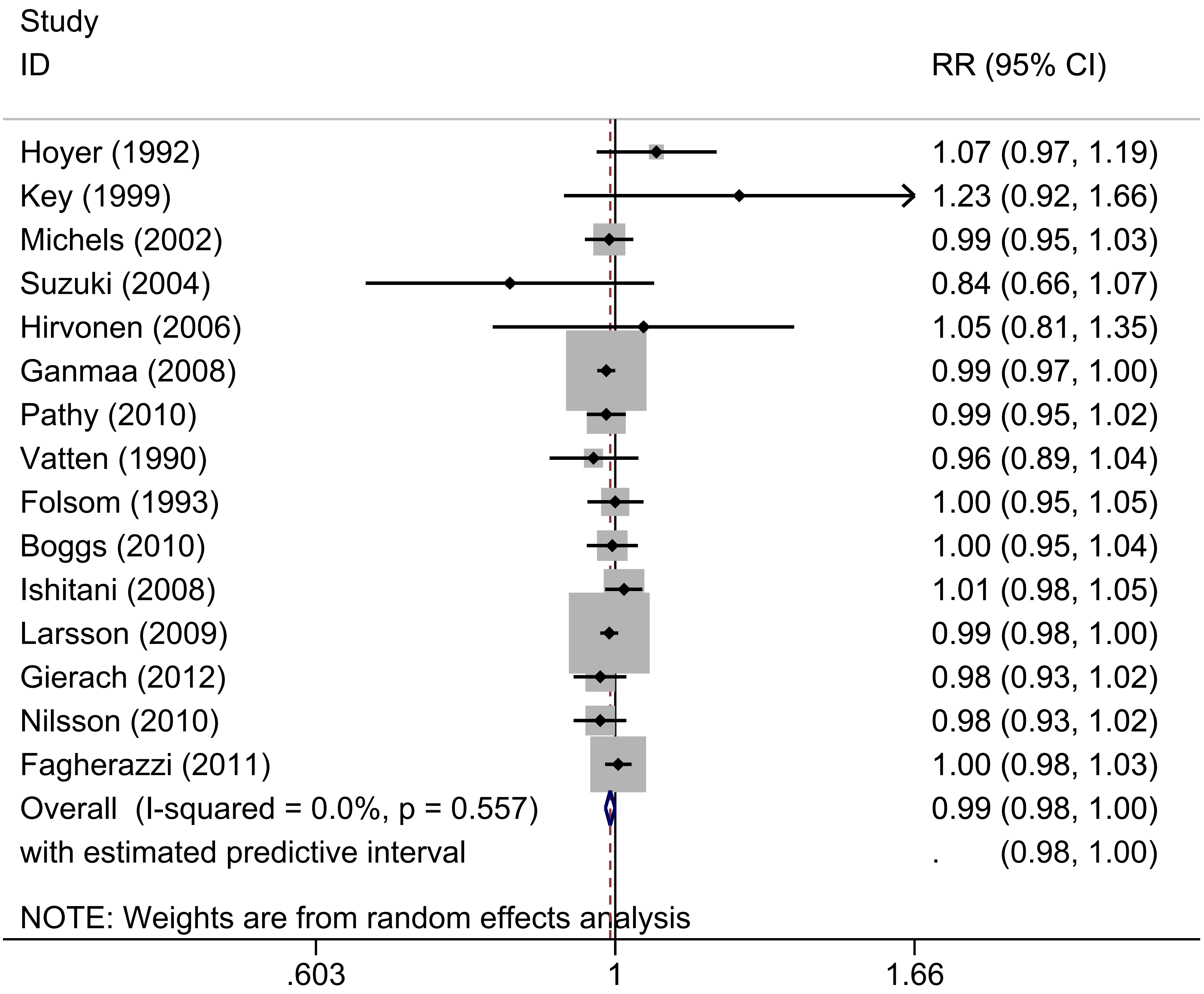

Supplementary figure 40, Colon cancer, H/L, only cohort studies

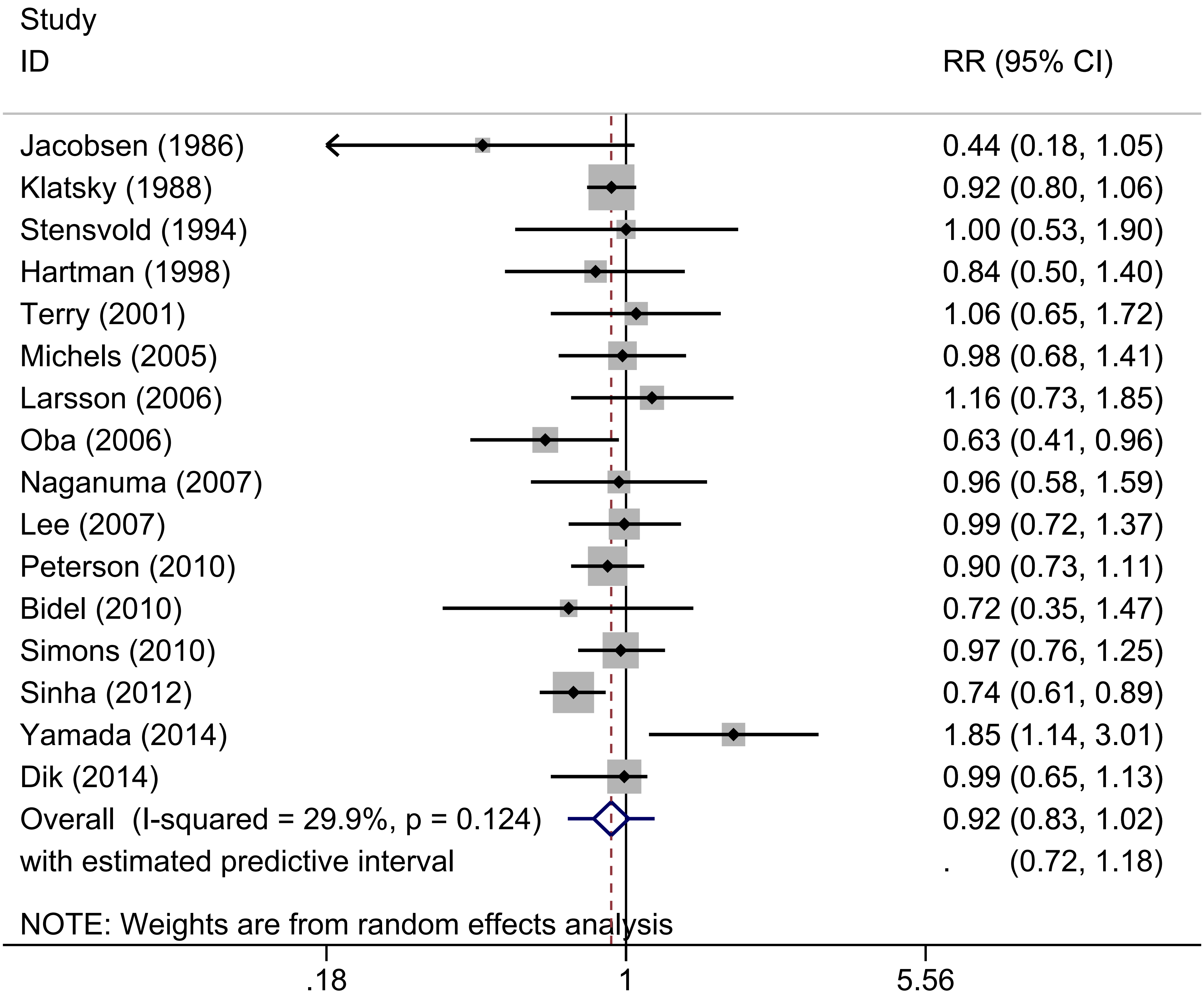

Supplementary figure 41, Colon cancer, Per 1 cup, only cohort studies

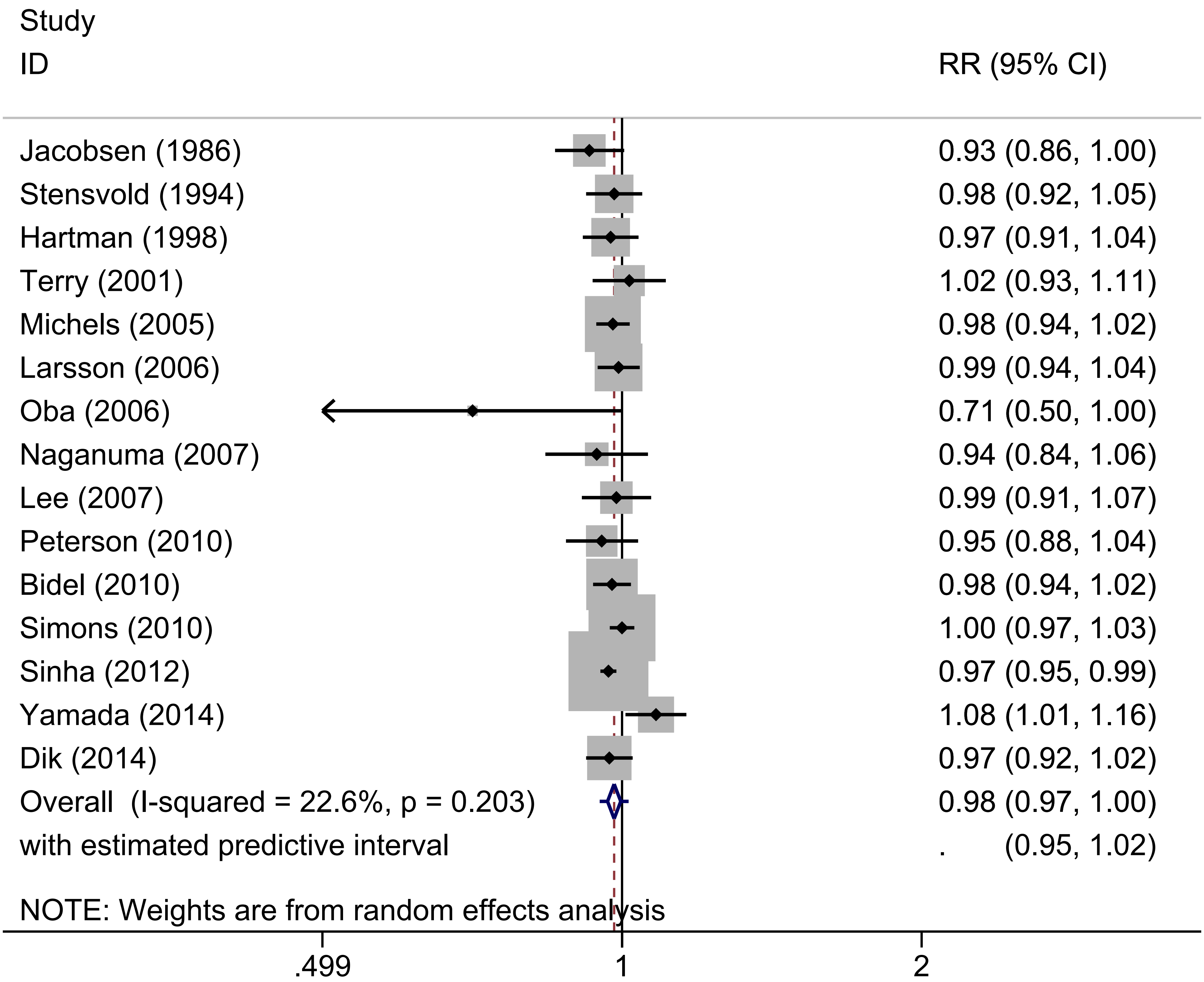

Supplementary figure 42, Colorectal cancer, H/L, only cohort studies

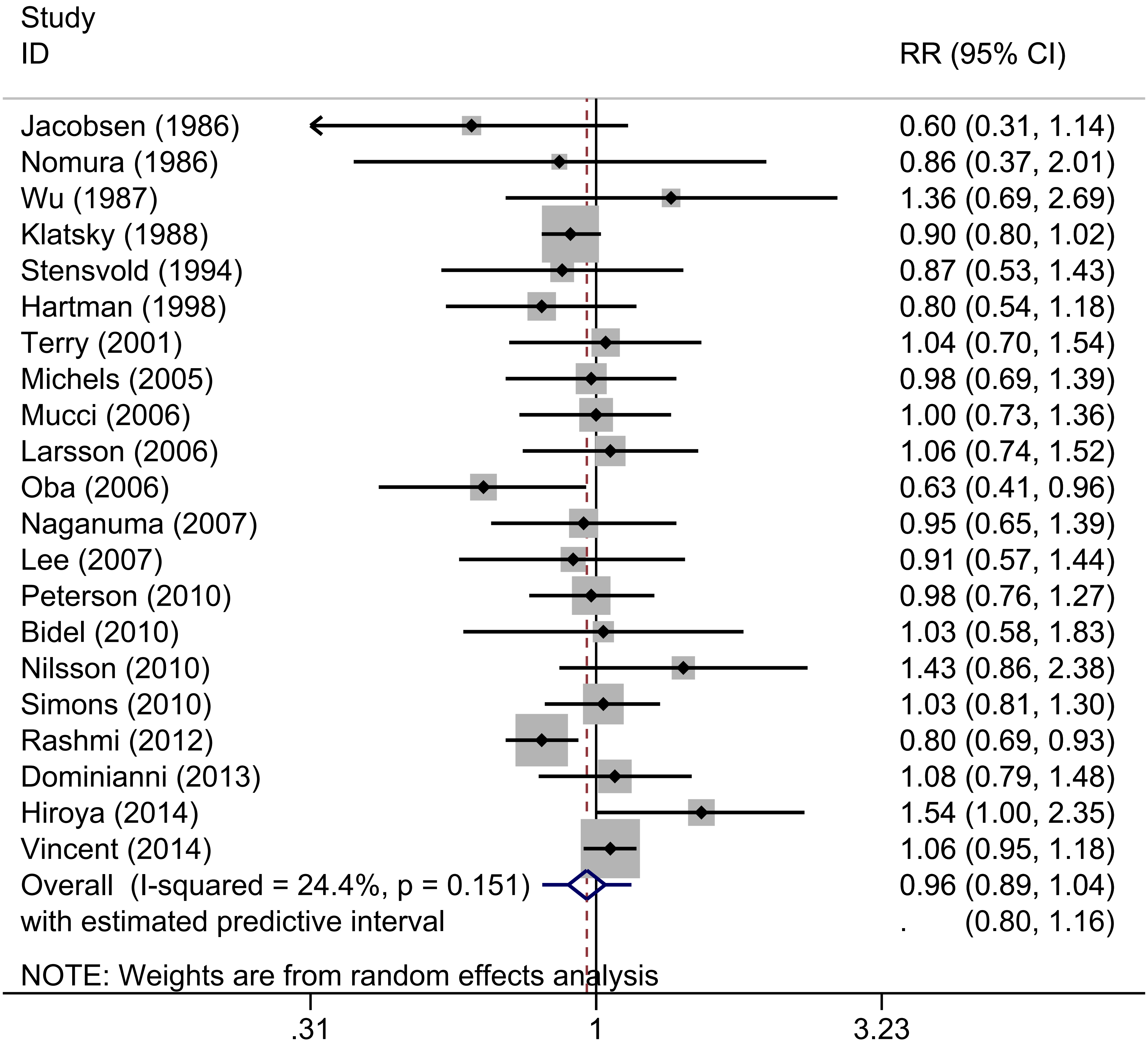

Supplementary figure 43, Colorectal cancer, Per 1 cup, only cohort studies

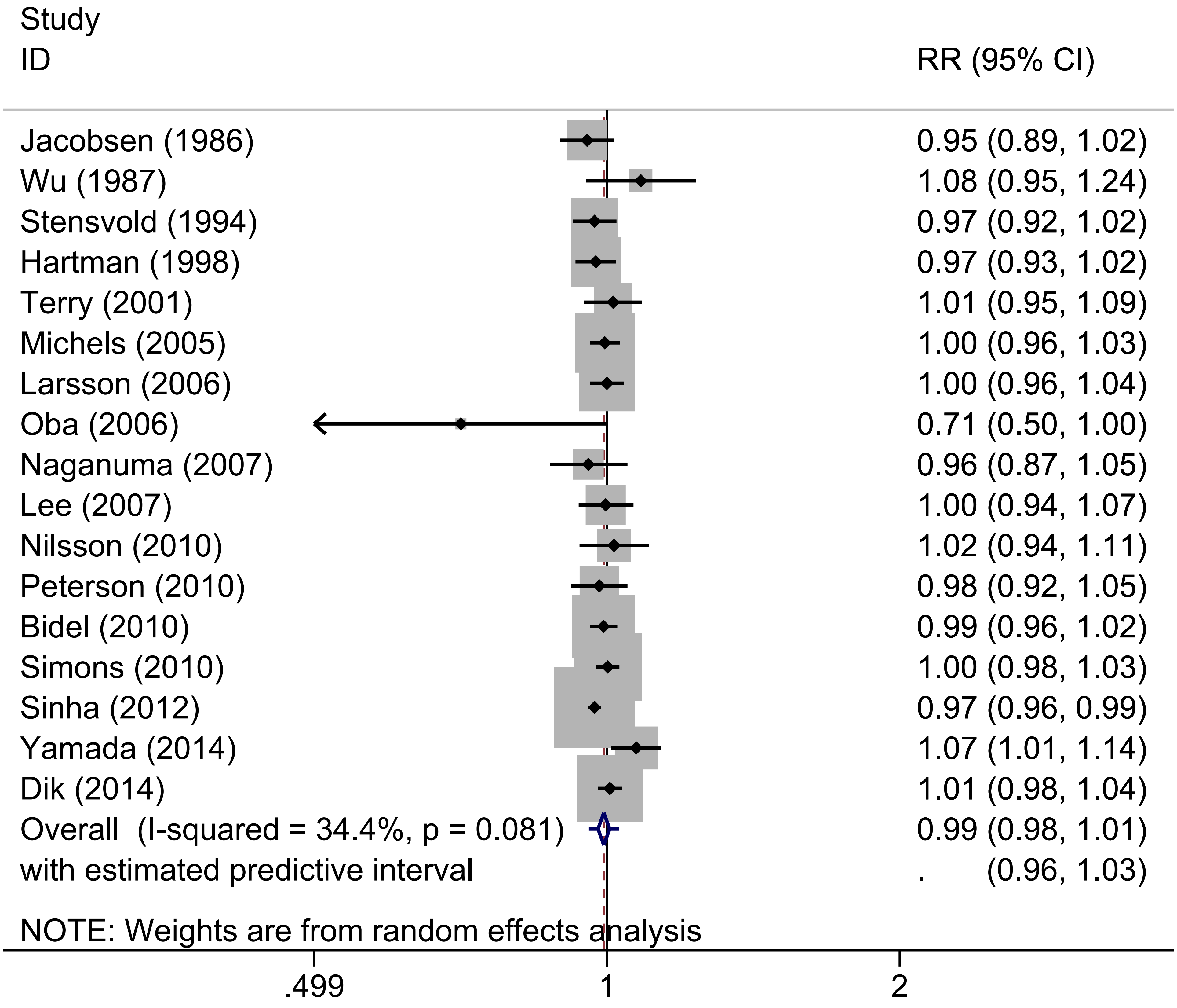

Supplementary figure 44, Endometrial cancer, H/L, only cohort studies

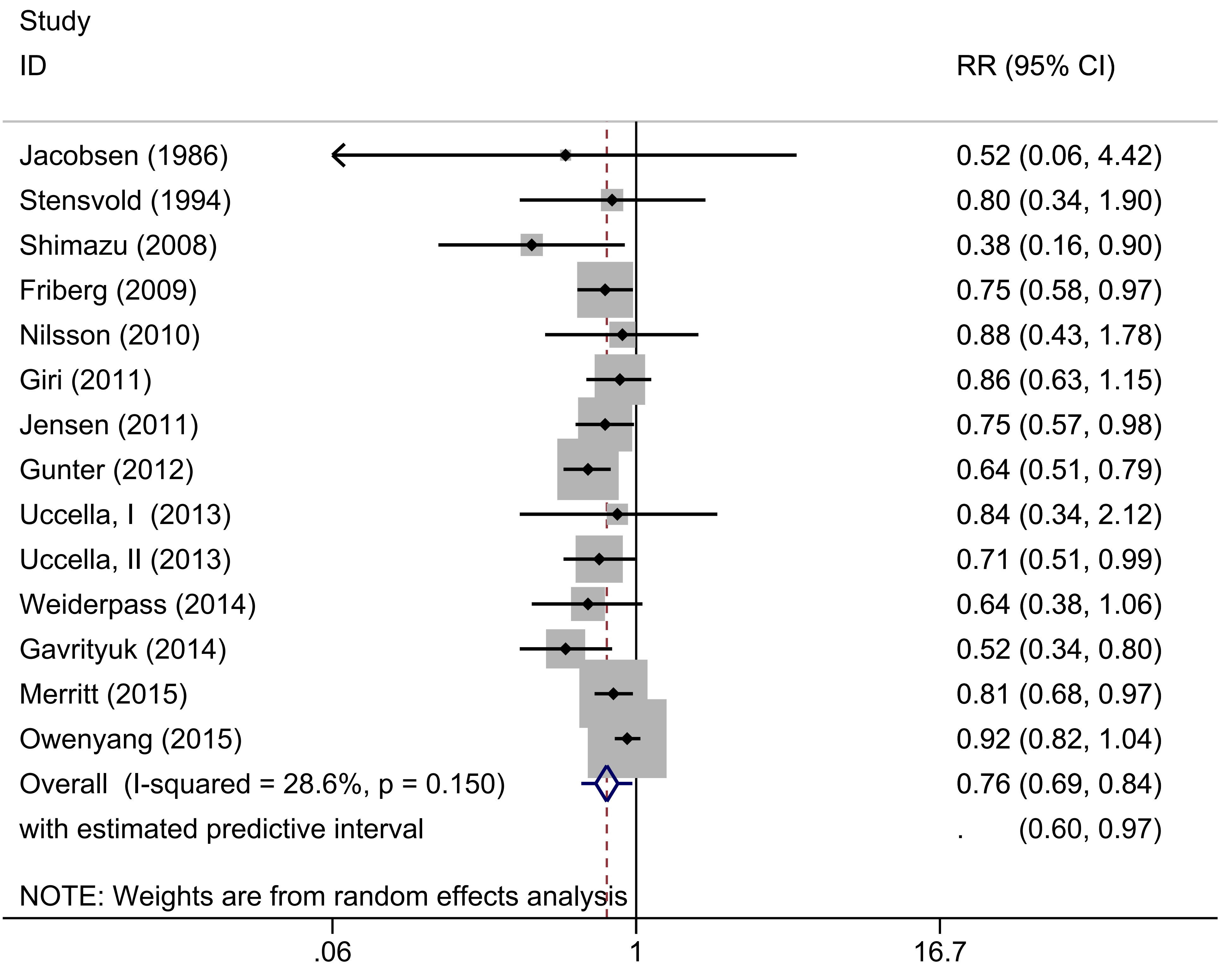

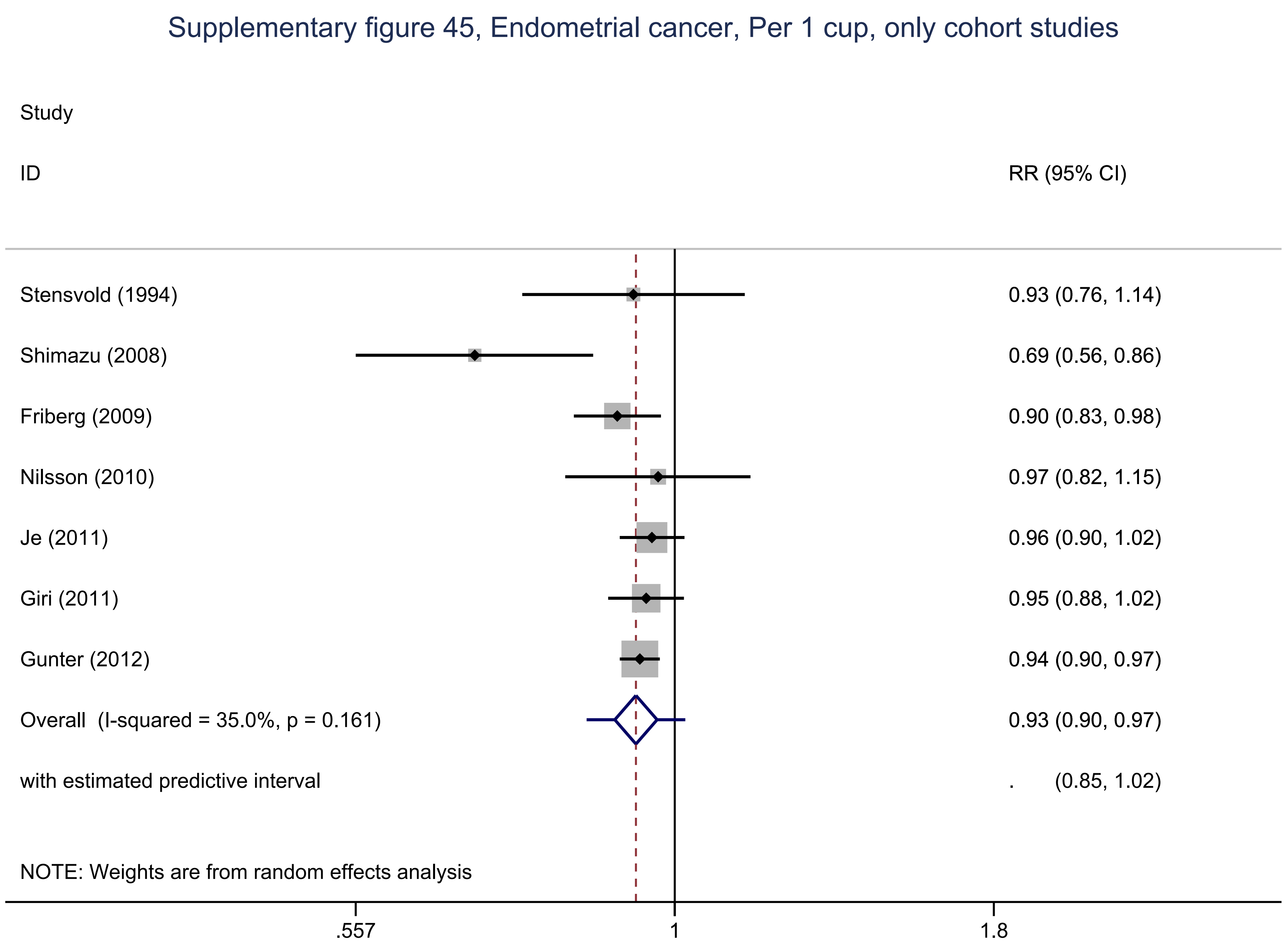
Supplementary figure 45, Endometrial cancer, Per 1 cup, only cohort studies
Study
ID
RR (95% CI)
Stensvold (1994)
0.93 (0.76, 1.14)
Shimazu (2008)
0.69 (0.56, 0.86)
Friberg (2009)
0.90 (0.83, 0.98)
Nilsson (2010)
0.97 (0.82, 1.15)
Je (2011)
0.96 (0.90, 1.02)
Giri (2011)
0.95 (0.88, 1.02)
Gunter (2012)
0.94 (0.90, 0.97)
Overall (I-squared = 35.0%, p = 0.161)
0.93 (0.90, 0.97)
with estimated predictive interval
. (0.85, 1.02)
NOTE: Weights are from random effects analysis
.557
1
1.8

Supplementary figure 46, Esophageal cancer, H/L, only cohort studies

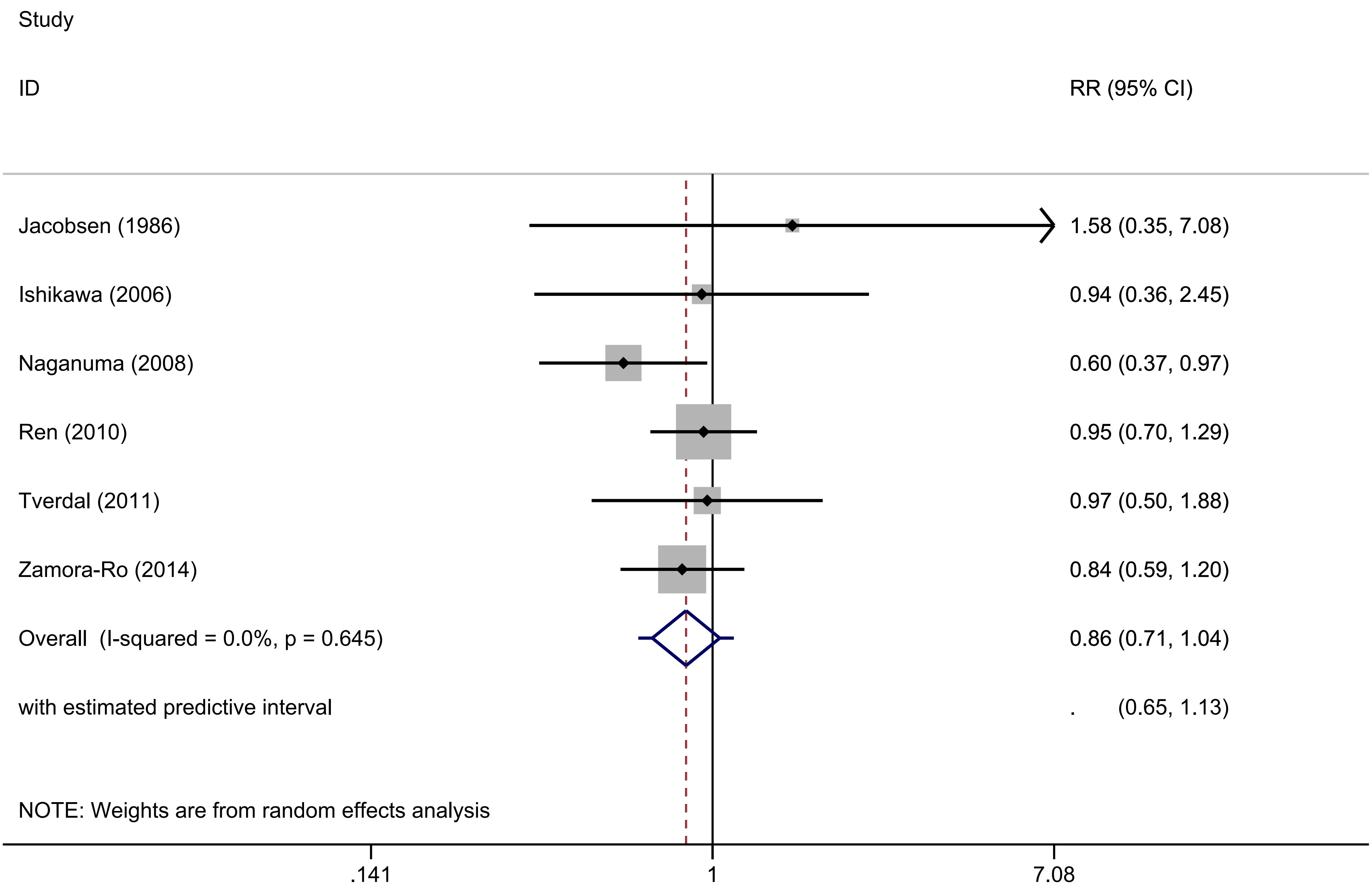

Supplementary figure 47, Gastric cancer, H/L, only cohort studies

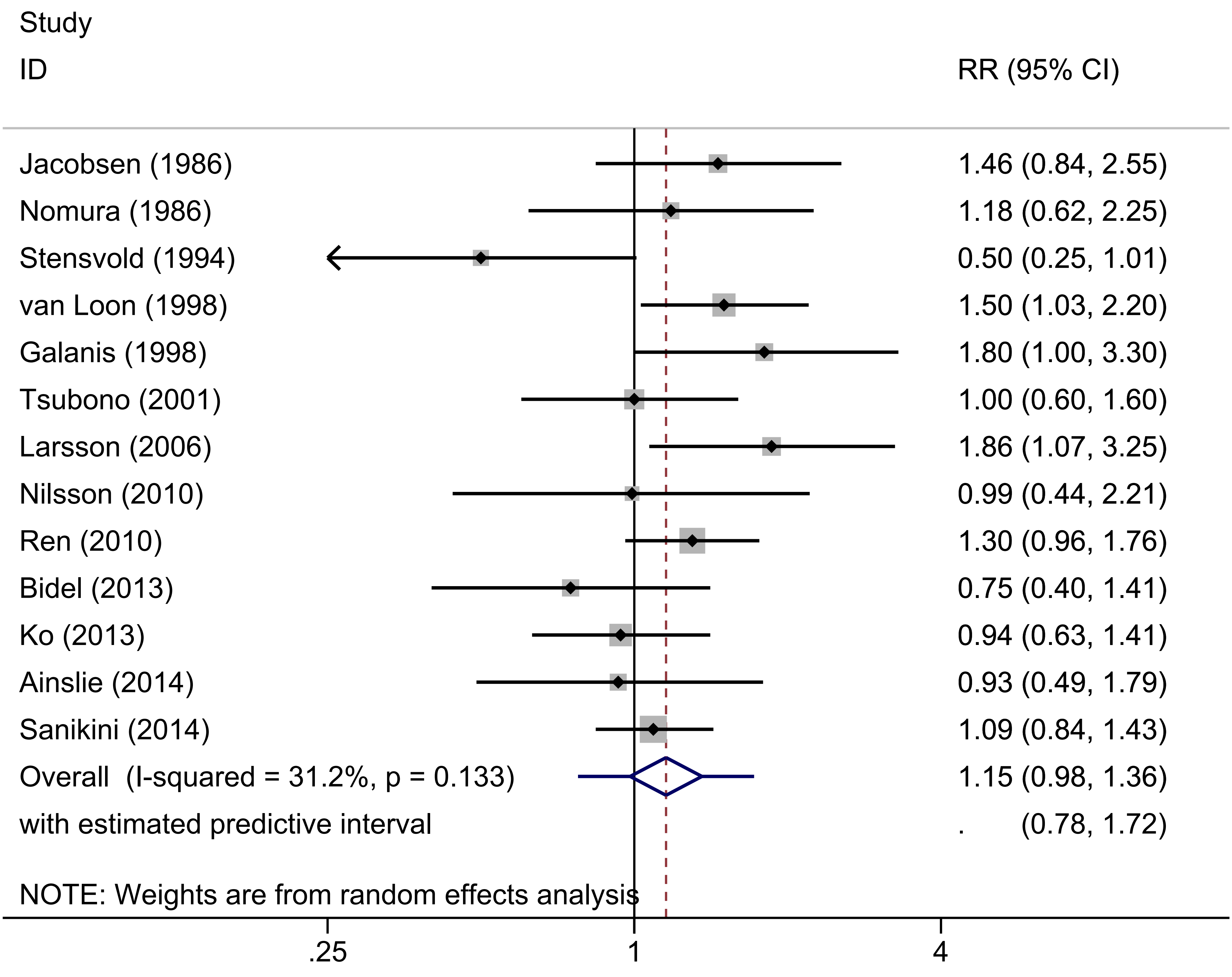

Supplementary figure 48, Gastric cancer, Per 1 cup, only cohort studies

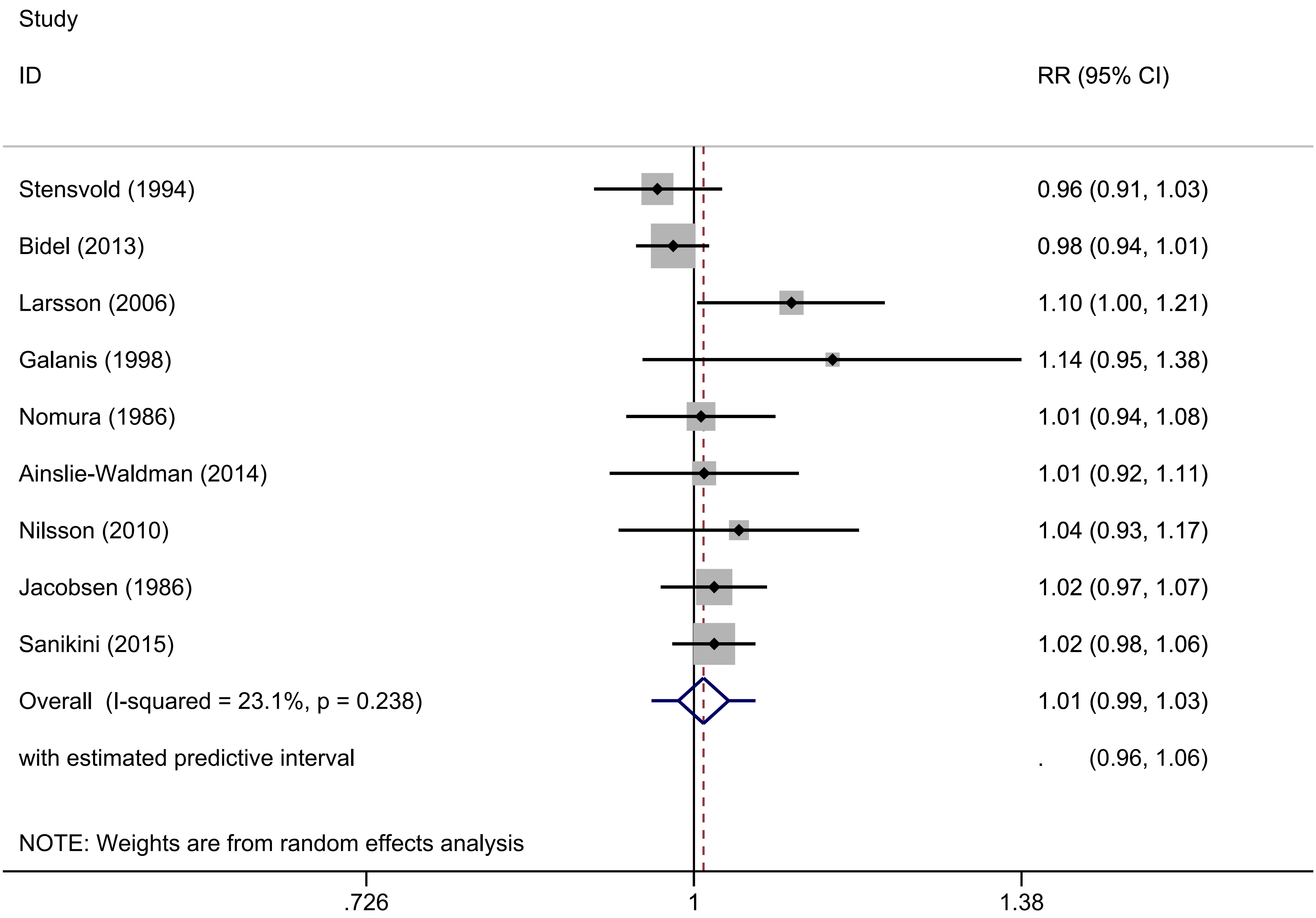

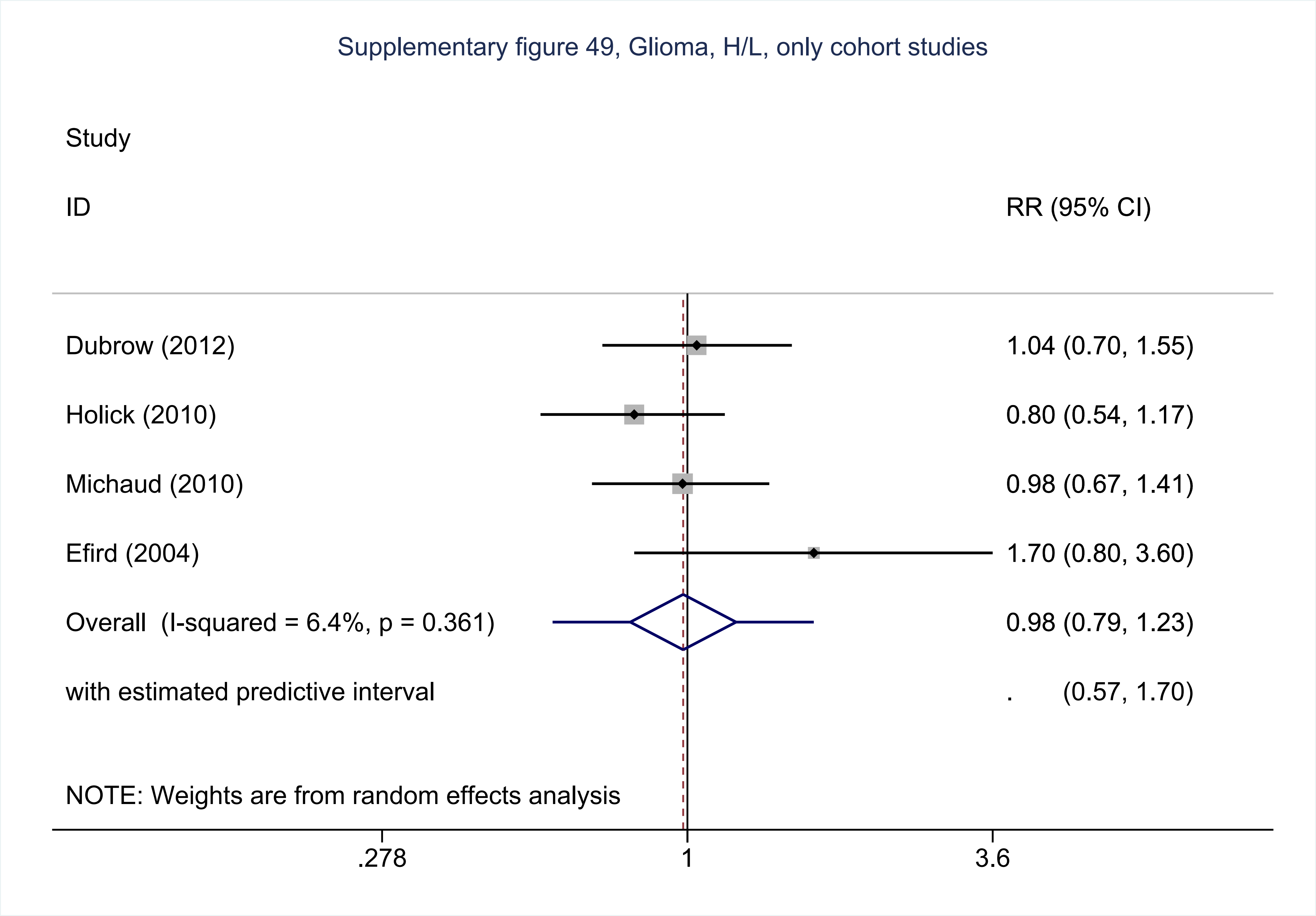

Supplementary figure 49, Glioma, H/L, only cohort studies
Study
ID
RR (95% CI)
Dubrow (2012)
1.04 (0.70, 1.55)
Holick (2010)
0.80 (0.54, 1.17)
Michaud (2010)
0.98 (0.67, 1.41)
Efird (2004)
1.70 (0.80, 3.60)
Overall (I-squared = 6.4%, p = 0.361)
0.98 (0.79, 1.23)
with estimated predictive interval
. (0.57, 1.70)
NOTE: Weights are from random effects analysis
.278
1
3.6

Supplementary figure 50, Kidney cancer, H/L, only cohort studies

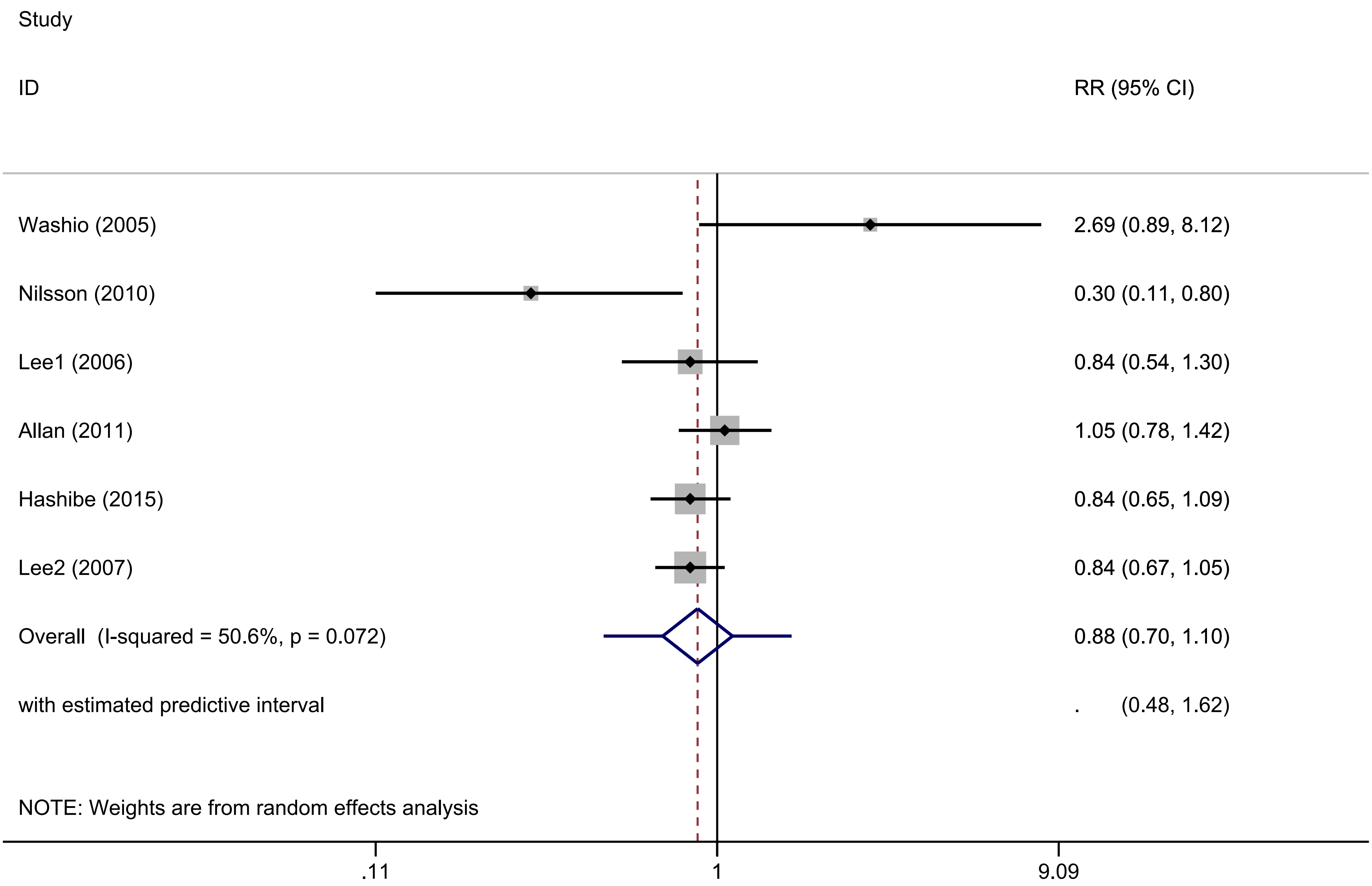

Supplementary figure 51, Laryngeal cancer, H/L, only cohort studies

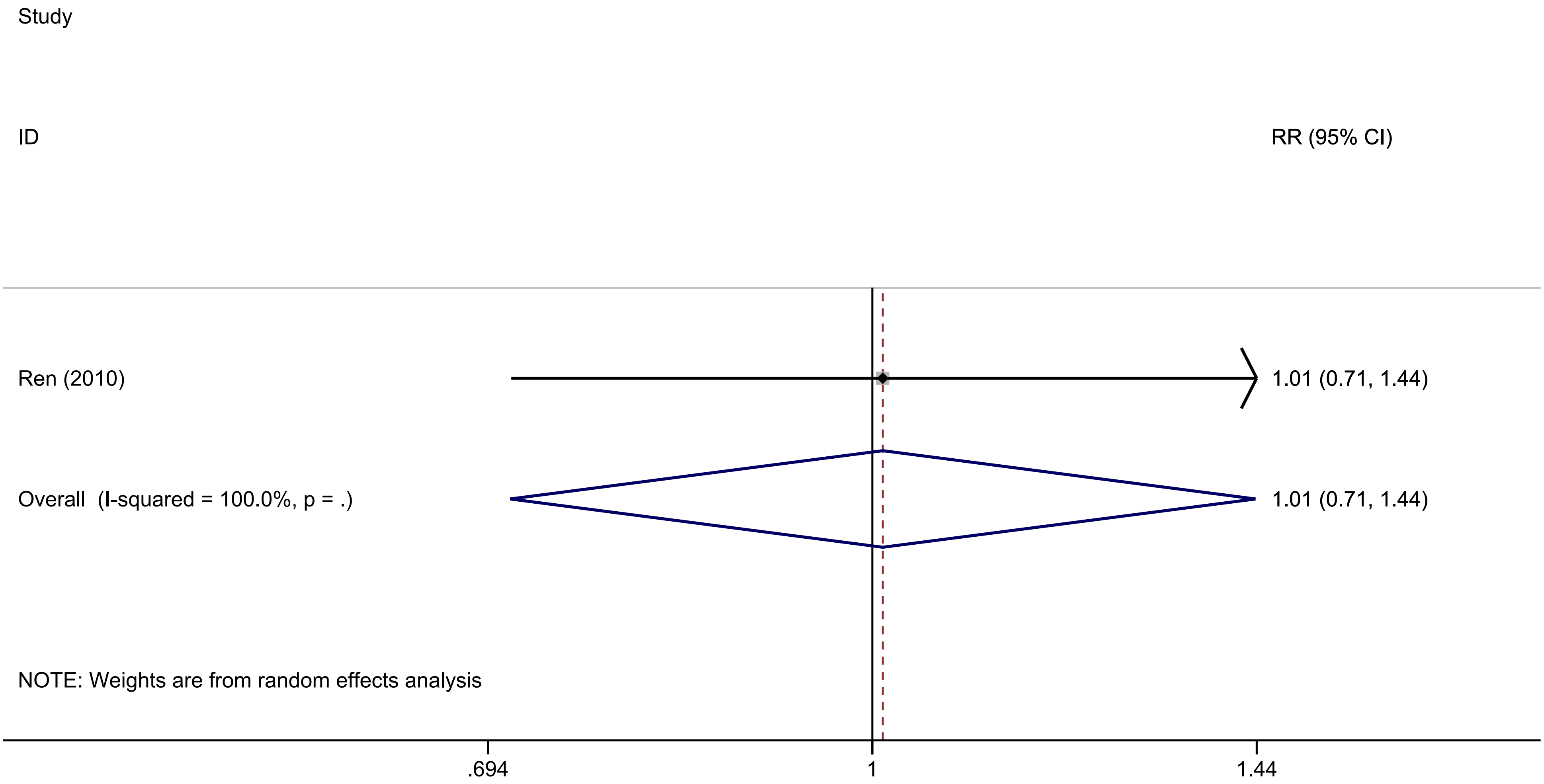

Supplementary figure 52, Leukemia, H/L, only cohort studies

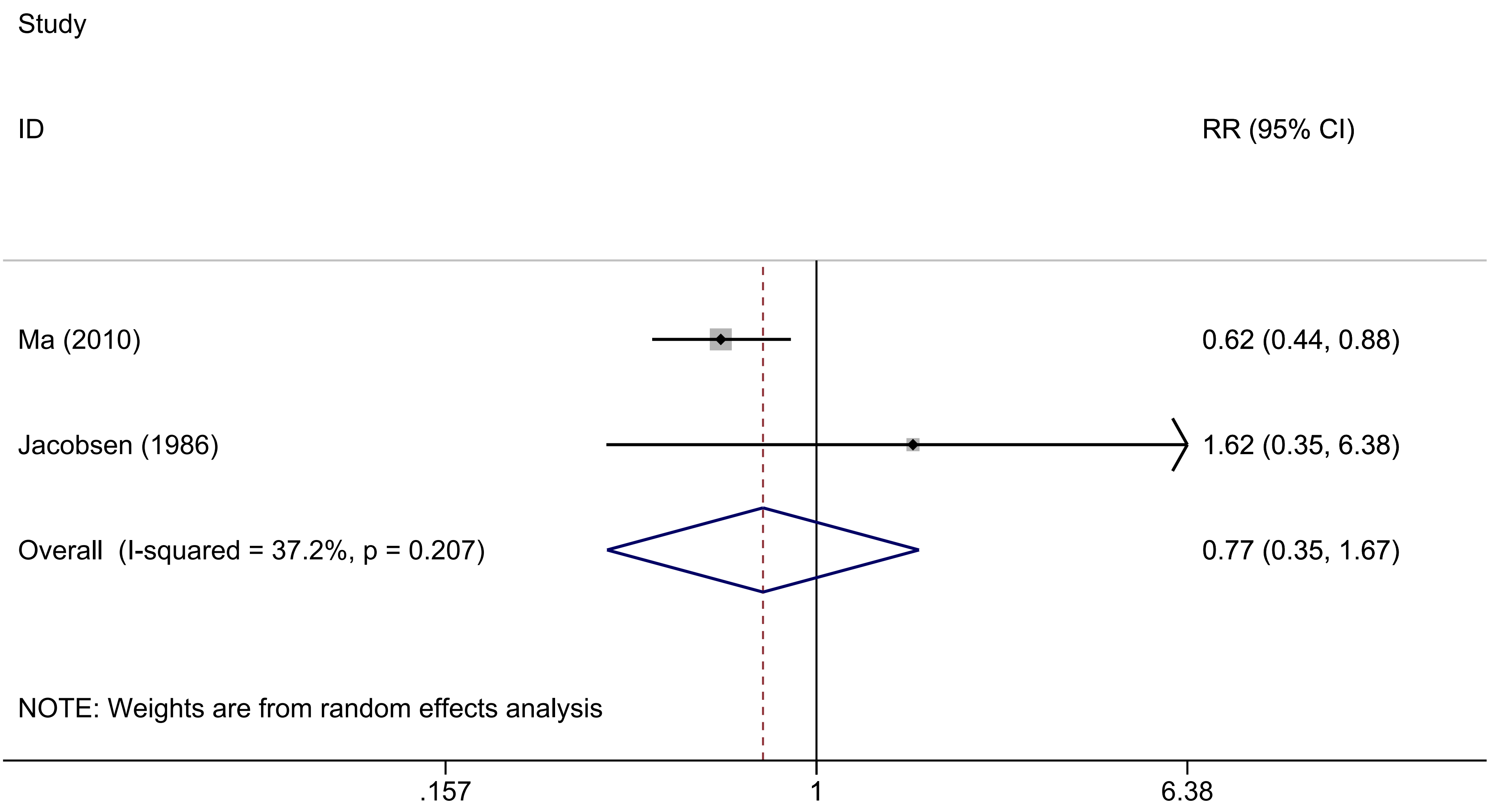

Supplementary figure 53, Liver cancer, H/L, only cohort studies

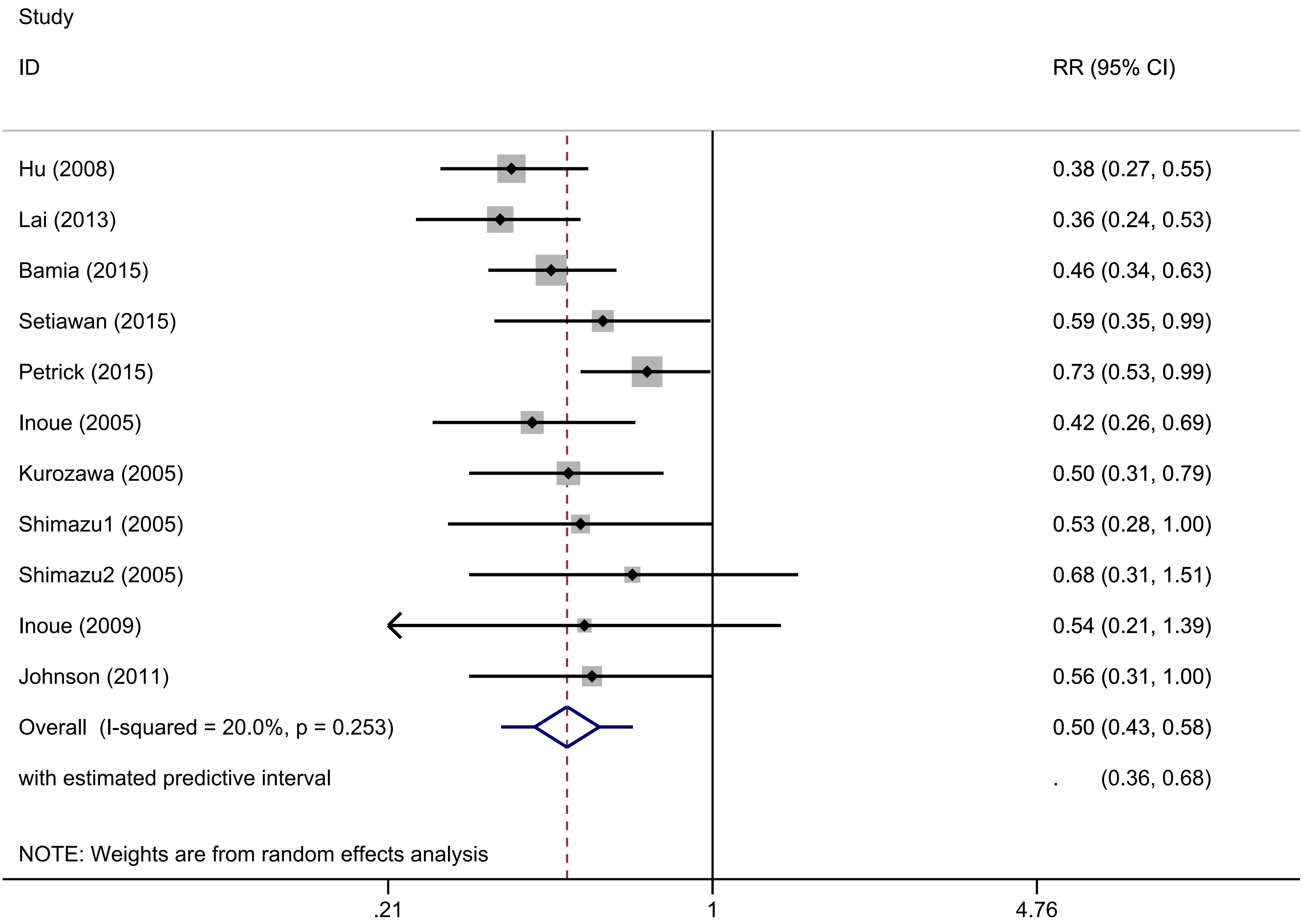

Supplementary figure 54, Liver cancer, Per 1 cup, only cohort studies

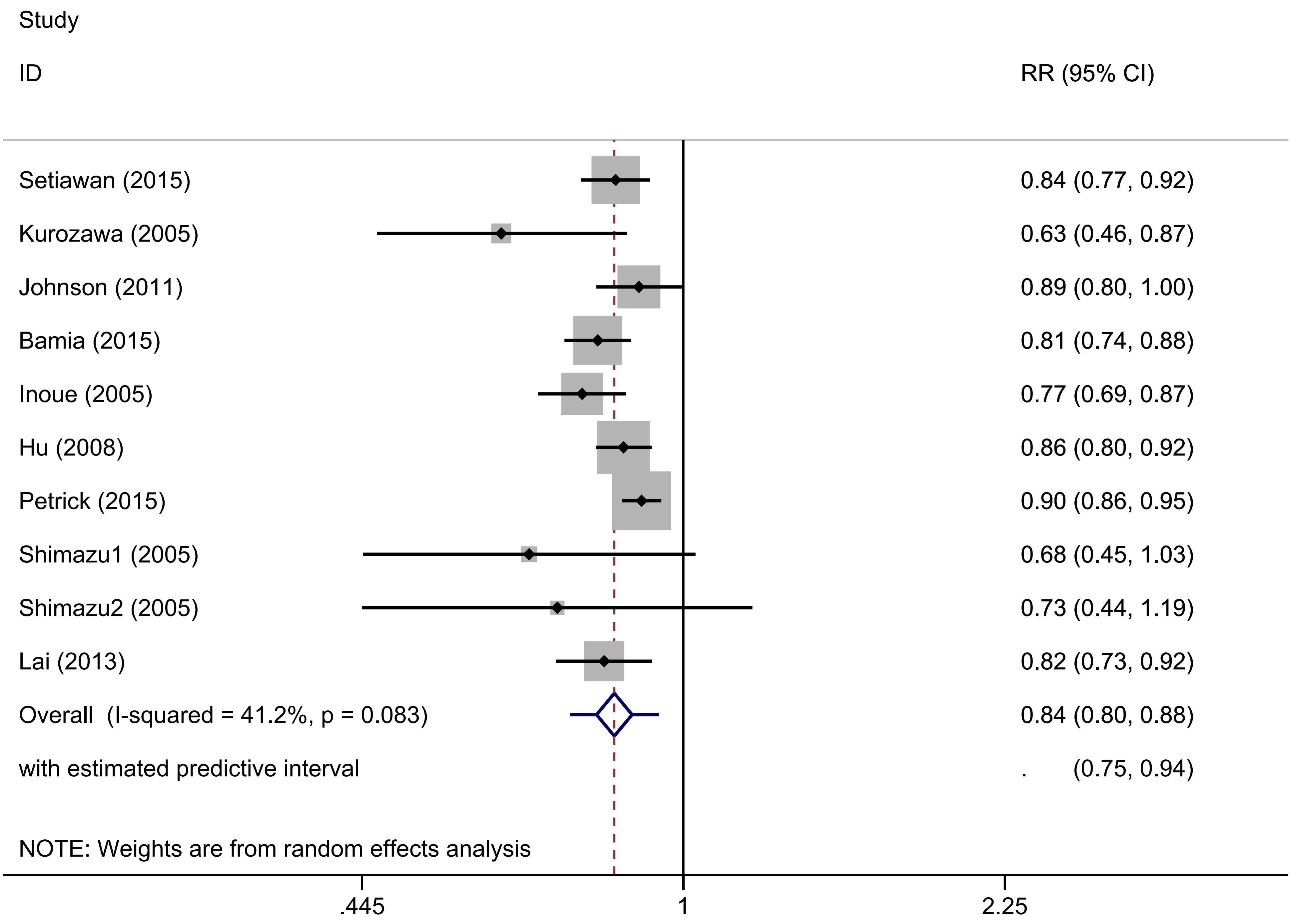

Supplementary figure 55, Lung cancer, H/L, only cohort studies

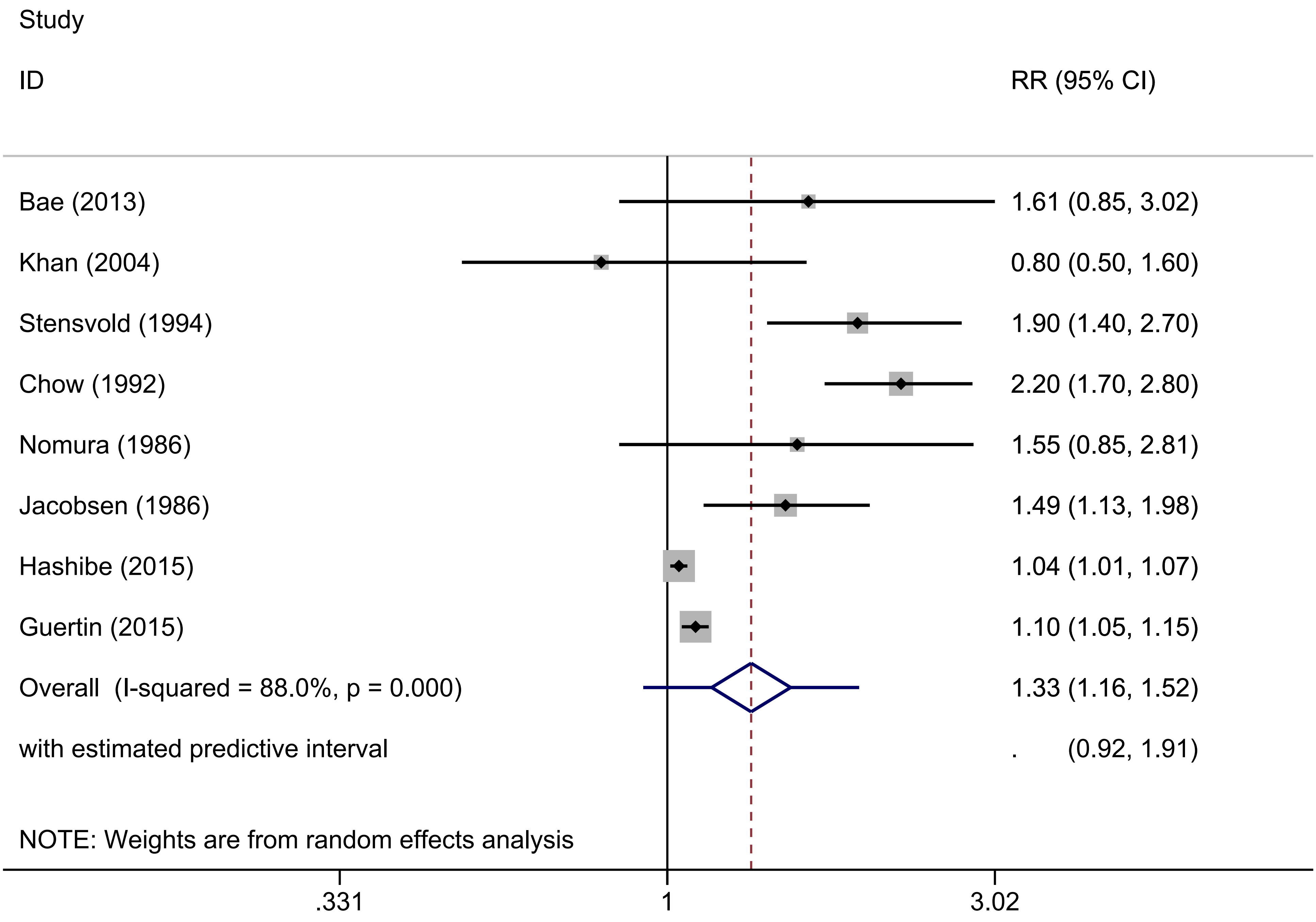

Supplementary figure 56, Lung cancer, Per 1 cup, only cohort studies

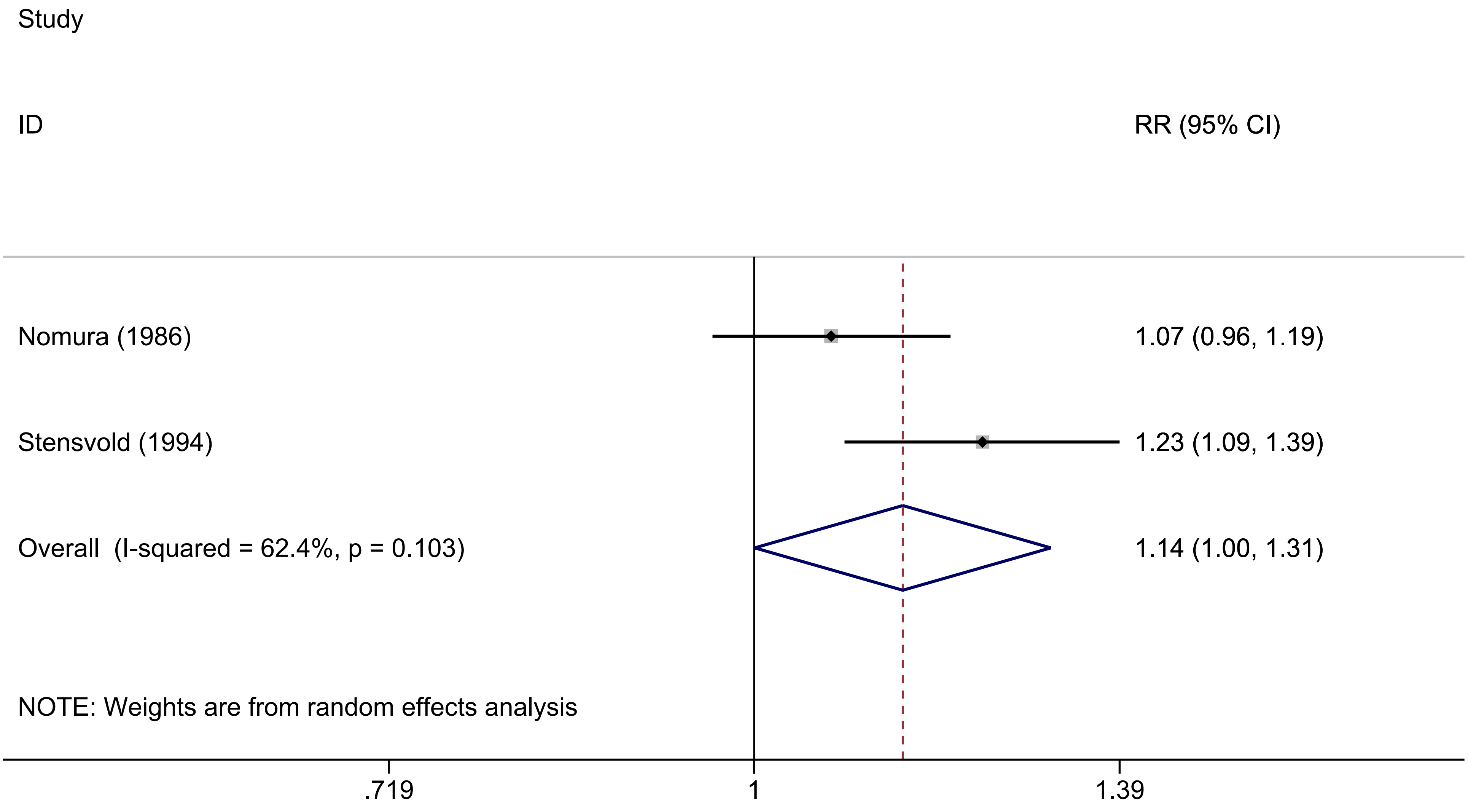

Supplementary figure 57, Lymphoma, H/L, only cohort studies

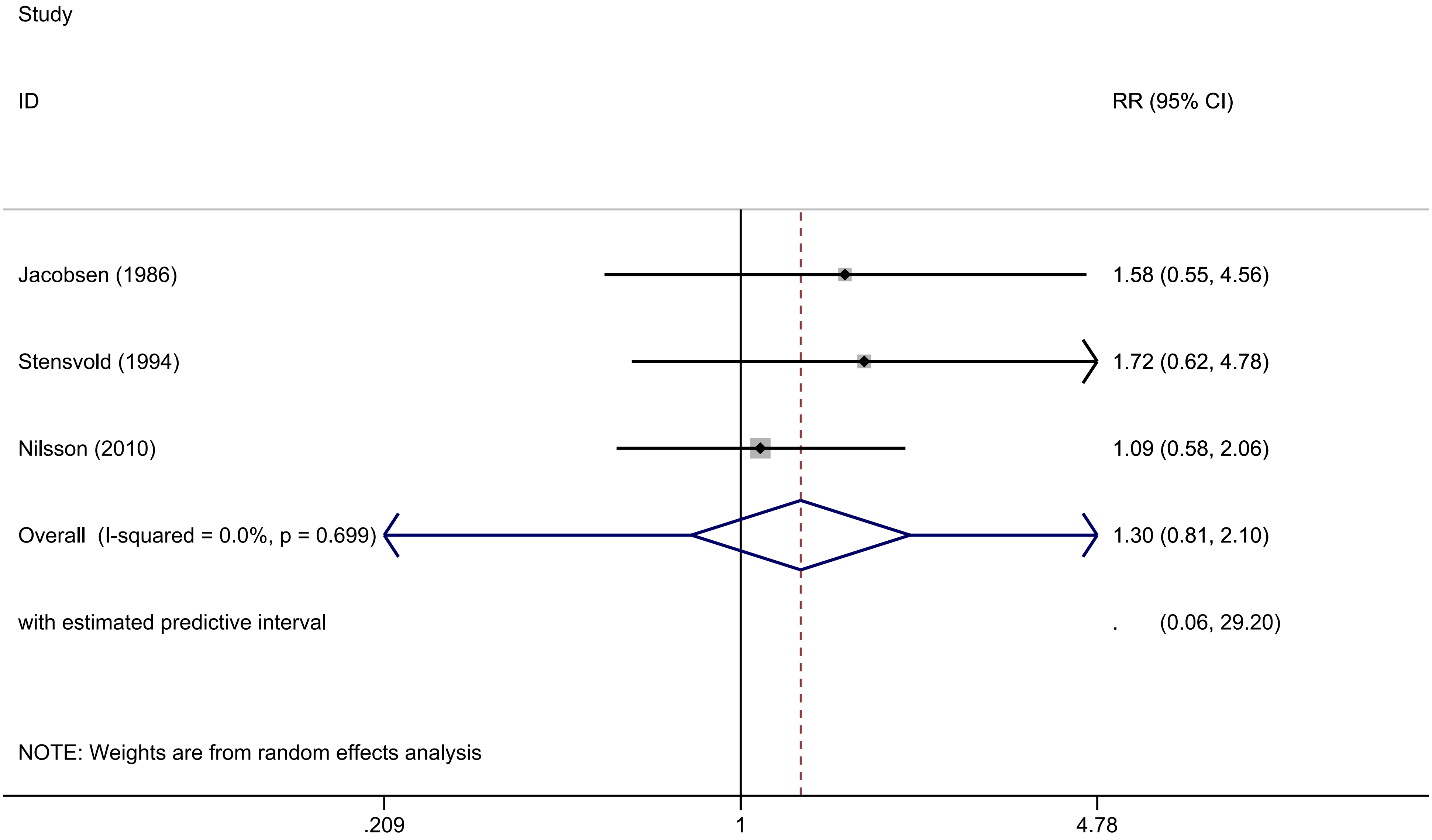

Supplementary figure 58, Melanoma, H/L, only cohort studies

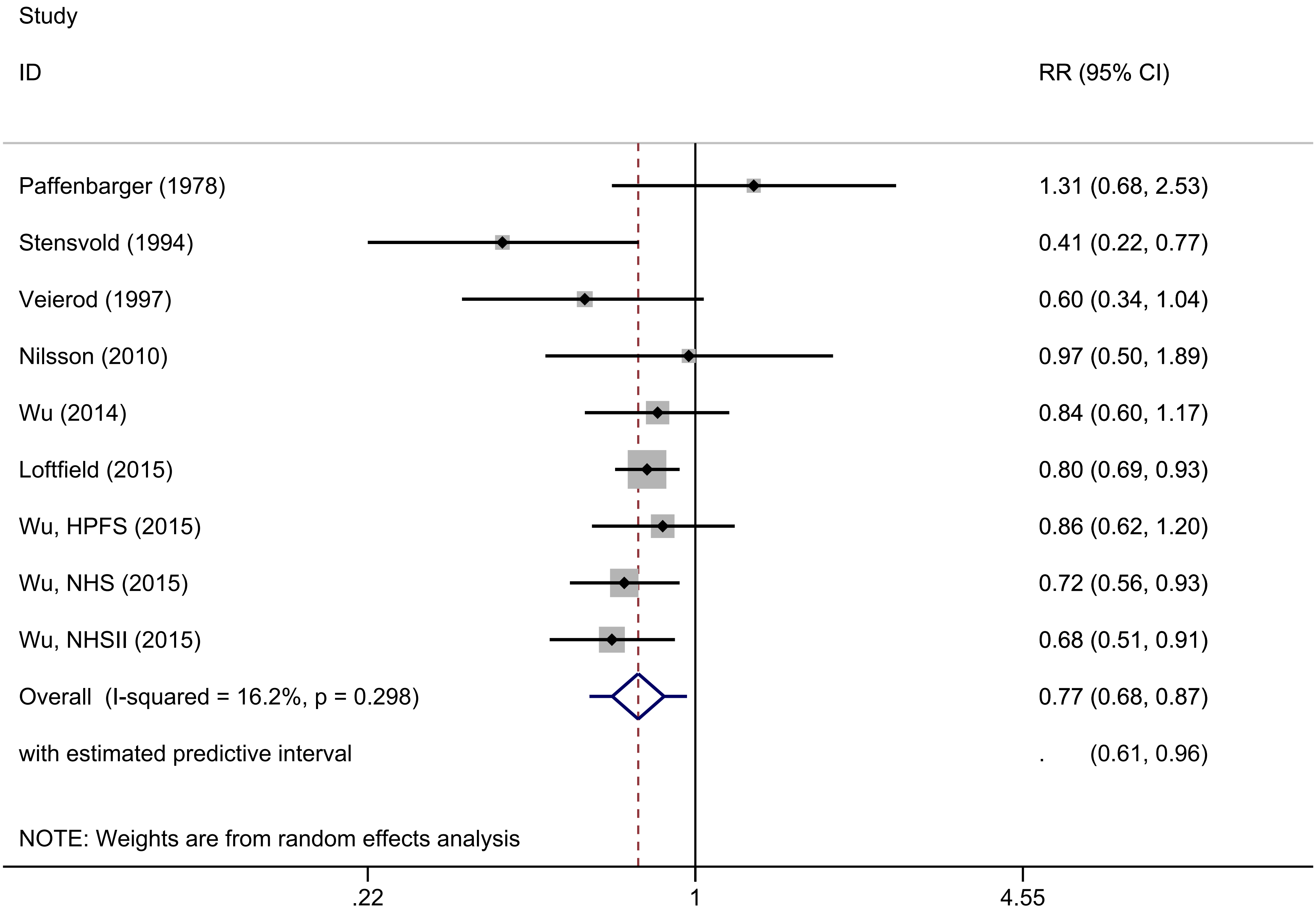

Supplementary figure 59, Nonmelanoma, H/L, only cohort studies

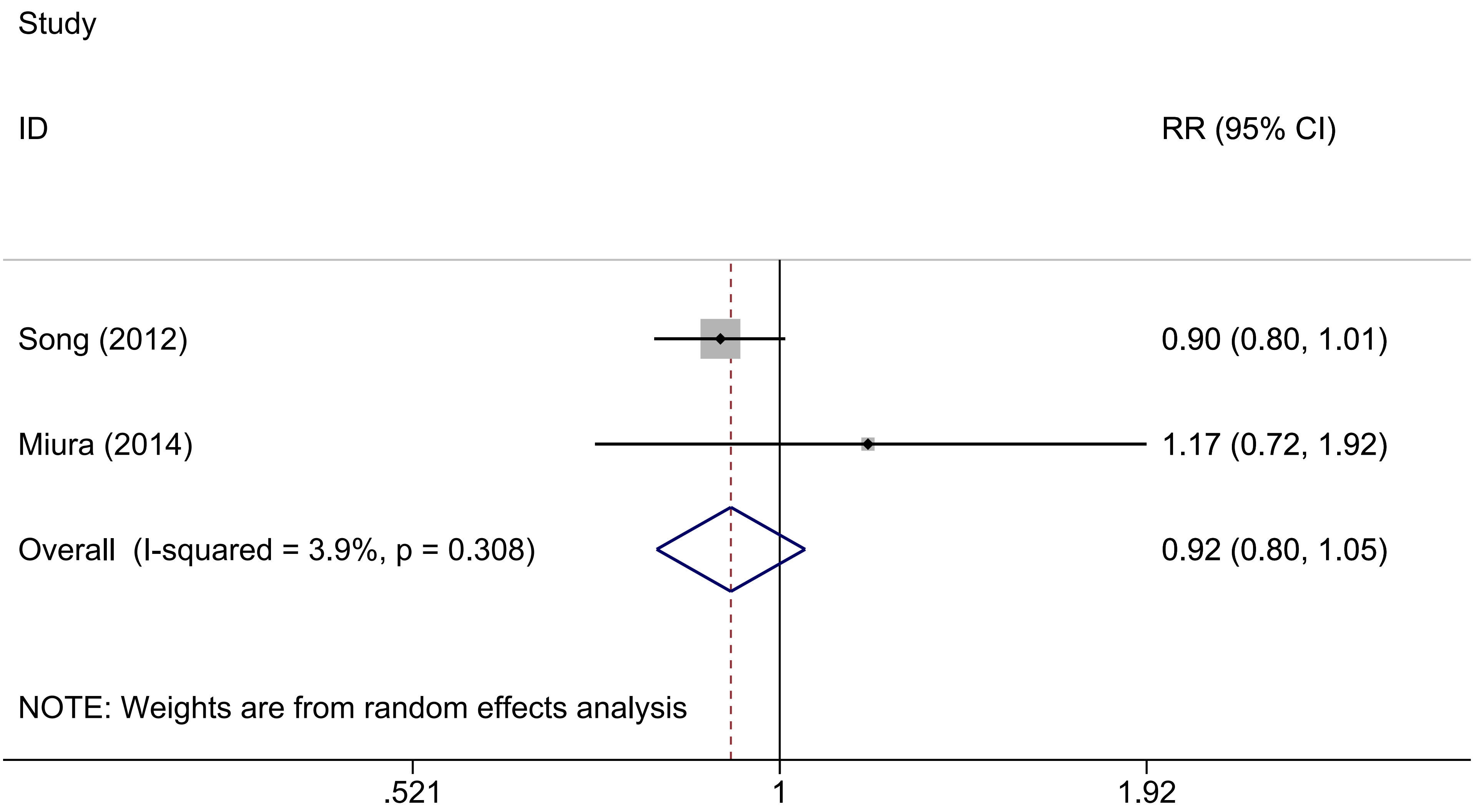

Supplementary figure 60, Oral cancer, H/L, only cohort studies

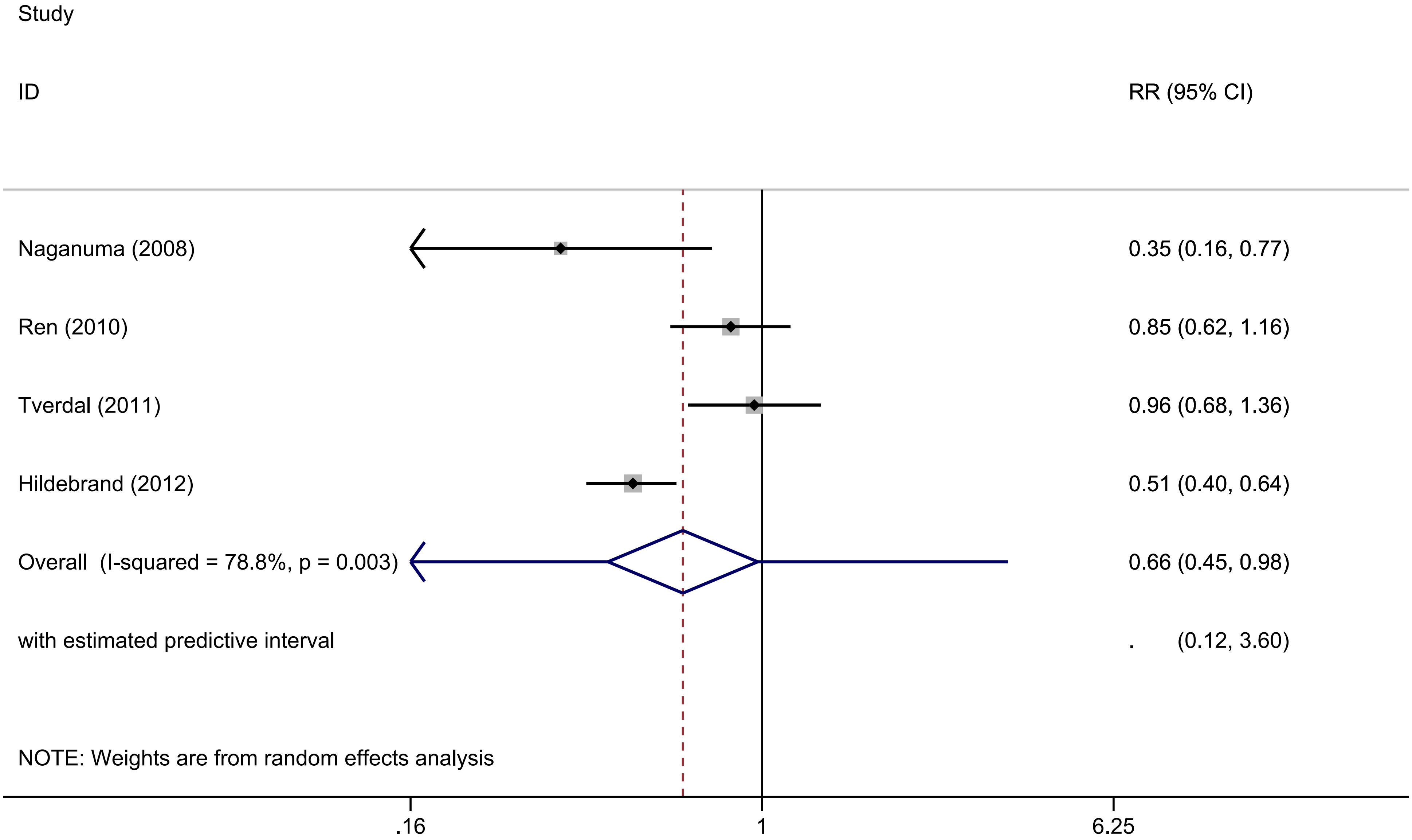

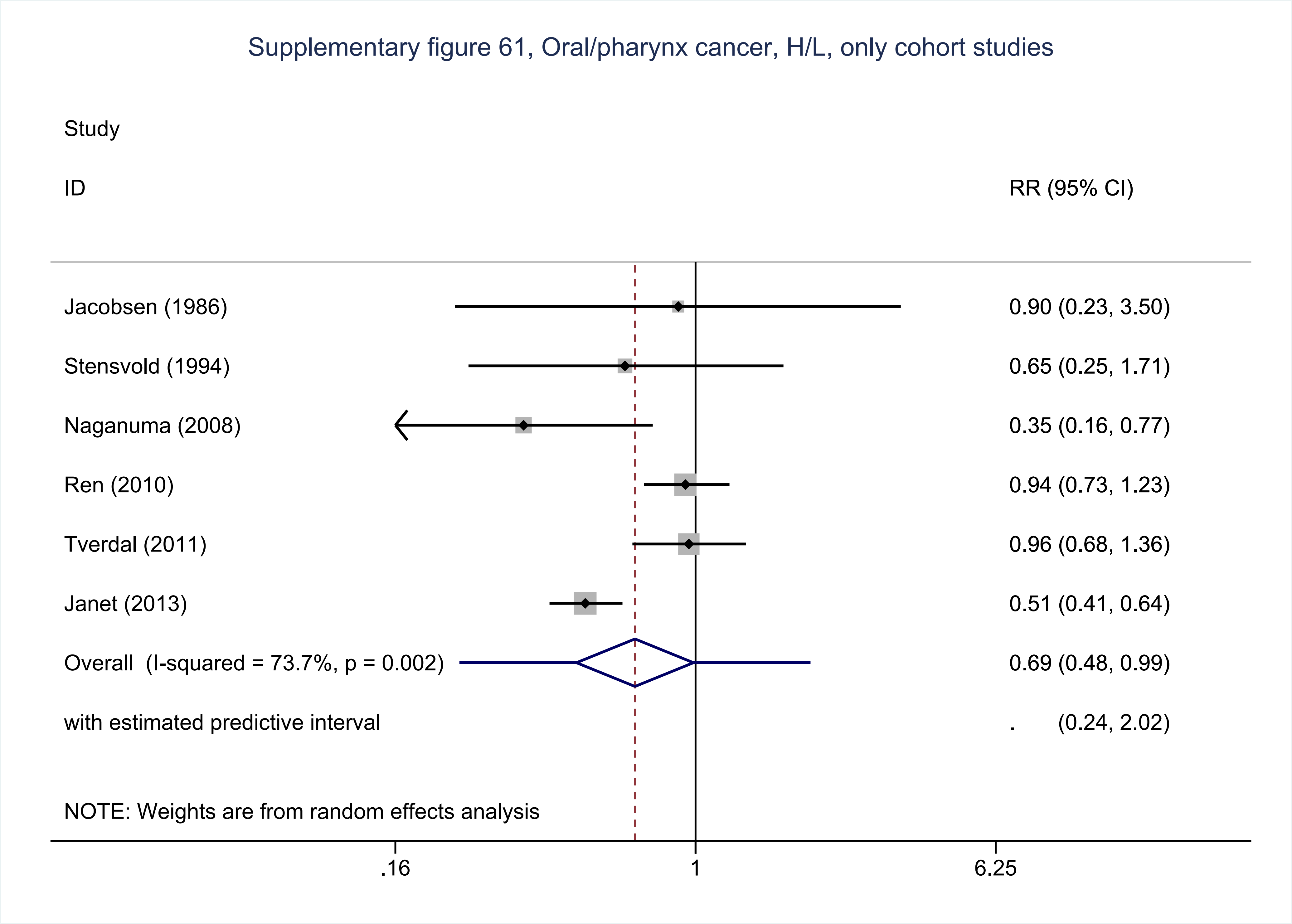
Supplementary figure 61, Oral/pharynx cancer, H/L, only cohort studies
Study
ID
RR (95% CI)
Jacobsen (1986)
0.90 (0.23, 3.50)
Stensvold (1994)
0.65 (0.25, 1.71)
Naganuma (2008)
0.35 (0.16, 0.77)
Ren (2010)
0.94 (0.73, 1.23)
Tverdal (2011)
0.96 (0.68, 1.36)
Janet (2013)
0.51 (0.41, 0.64)
Overall (I-squared = 73.7%, p = 0.002)
0.69 (0.48, 0.99)
with estimated predictive interval
. (0.24, 2.02)
NOTE: Weights are from random effects analysis
.16
1
6.25

Supplementary figure 62, Ovarian cancer, H/L, only cohort studies

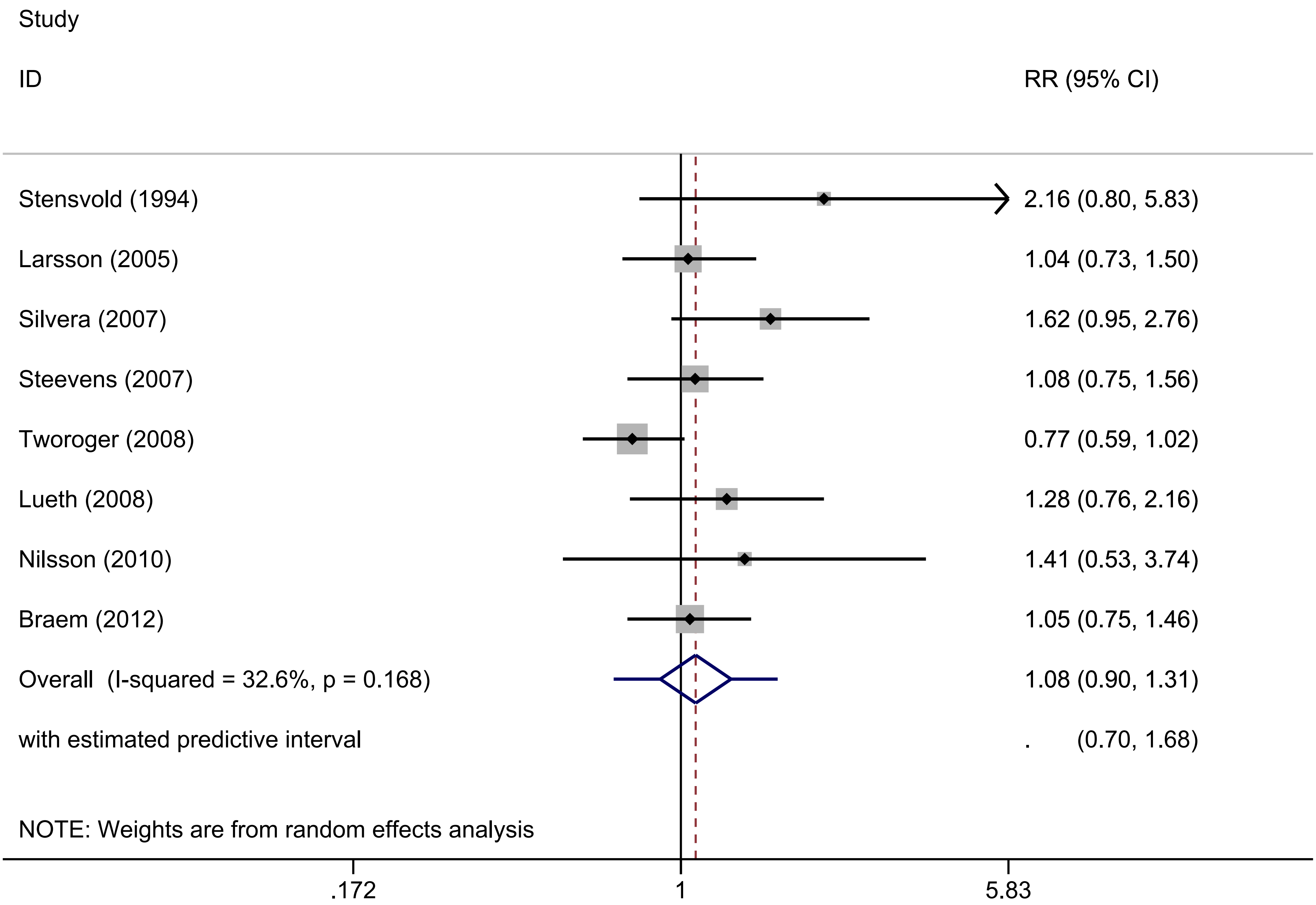

Supplementary figure 63, Pancreatic cancer, H/L, only cohort studies

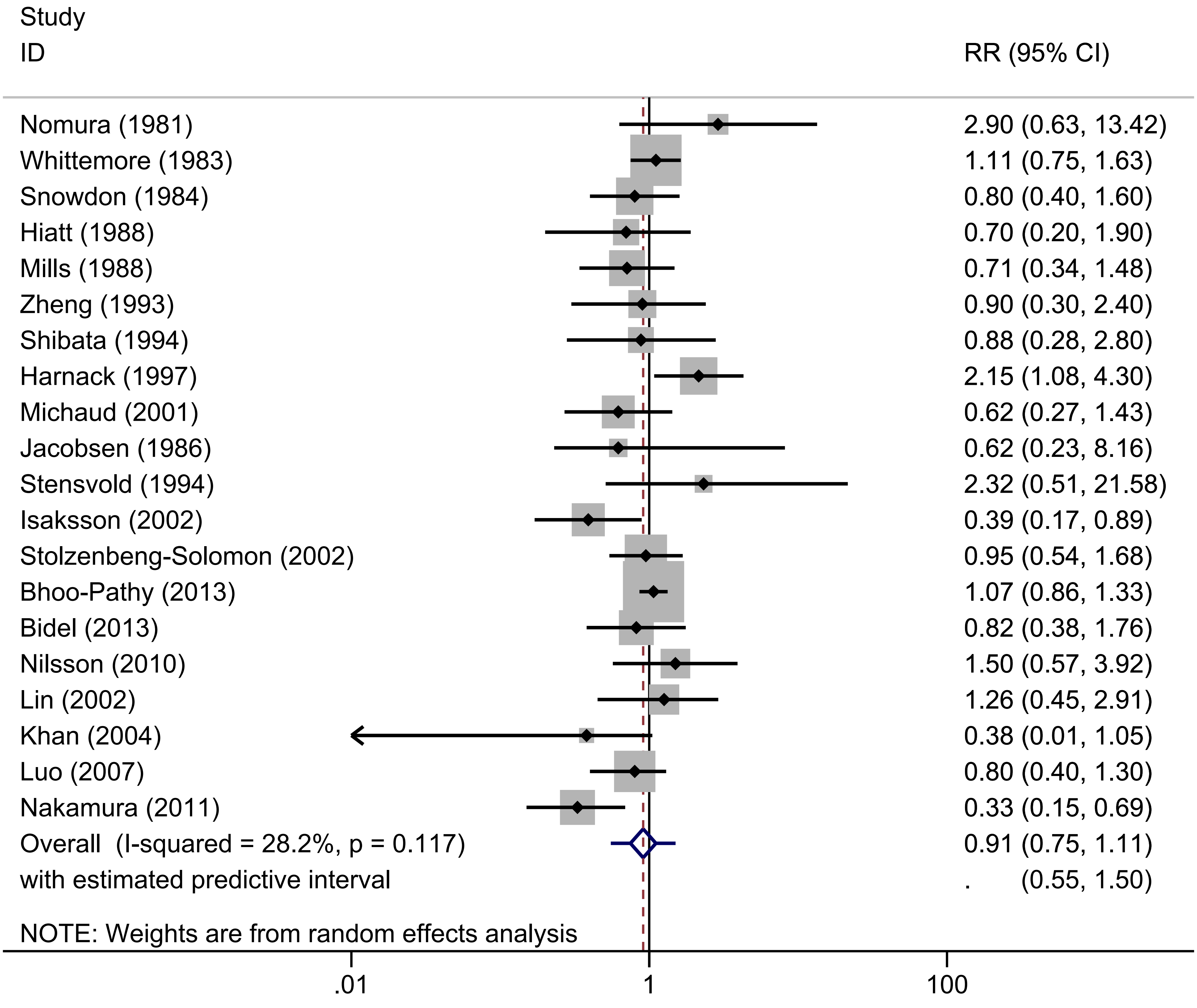

Supplementary figure 64, Pancreatic cancer, Per 1 cup, only cohort studies

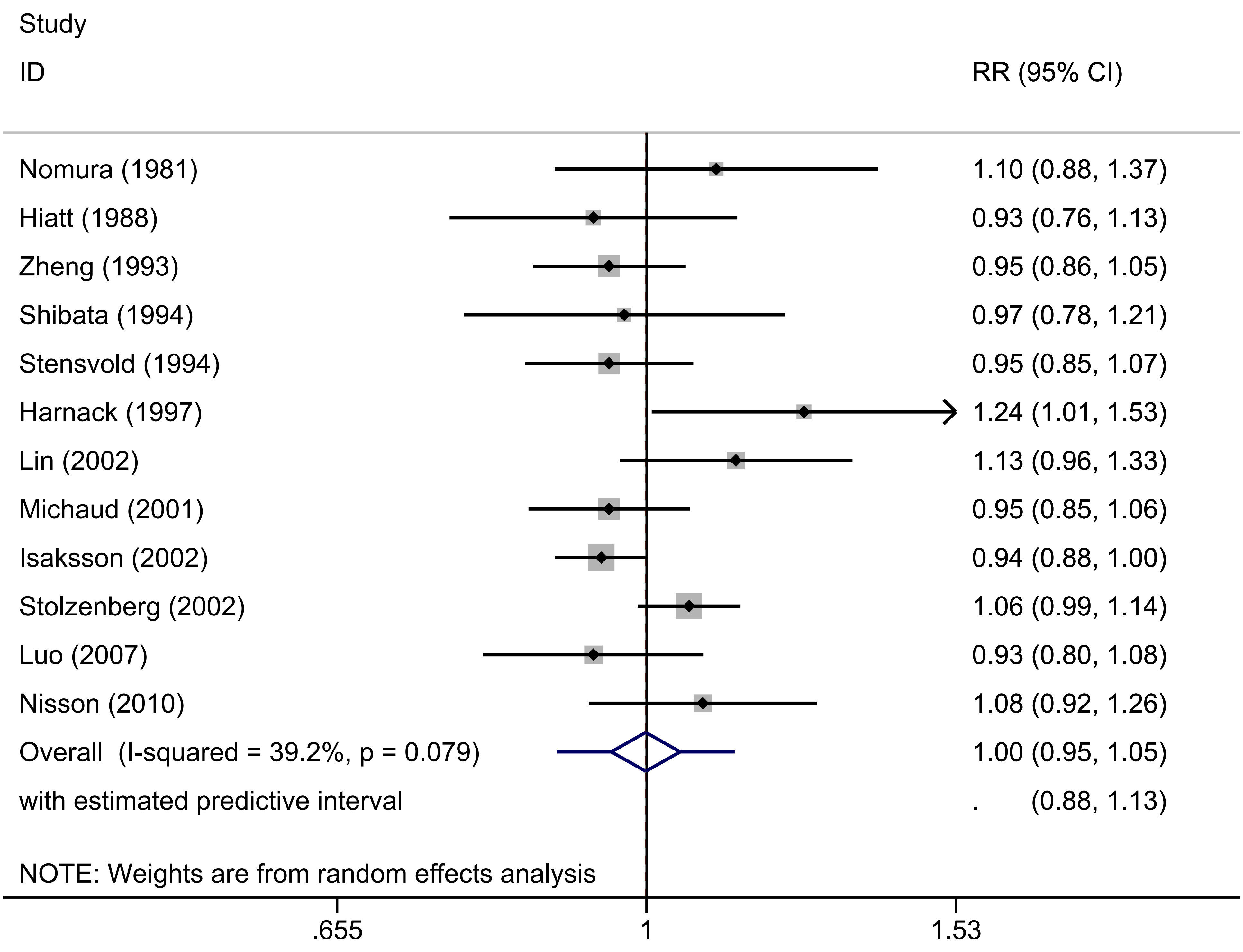

Supplementary figure 65, Prostate cancer, H/L, only cohort studies

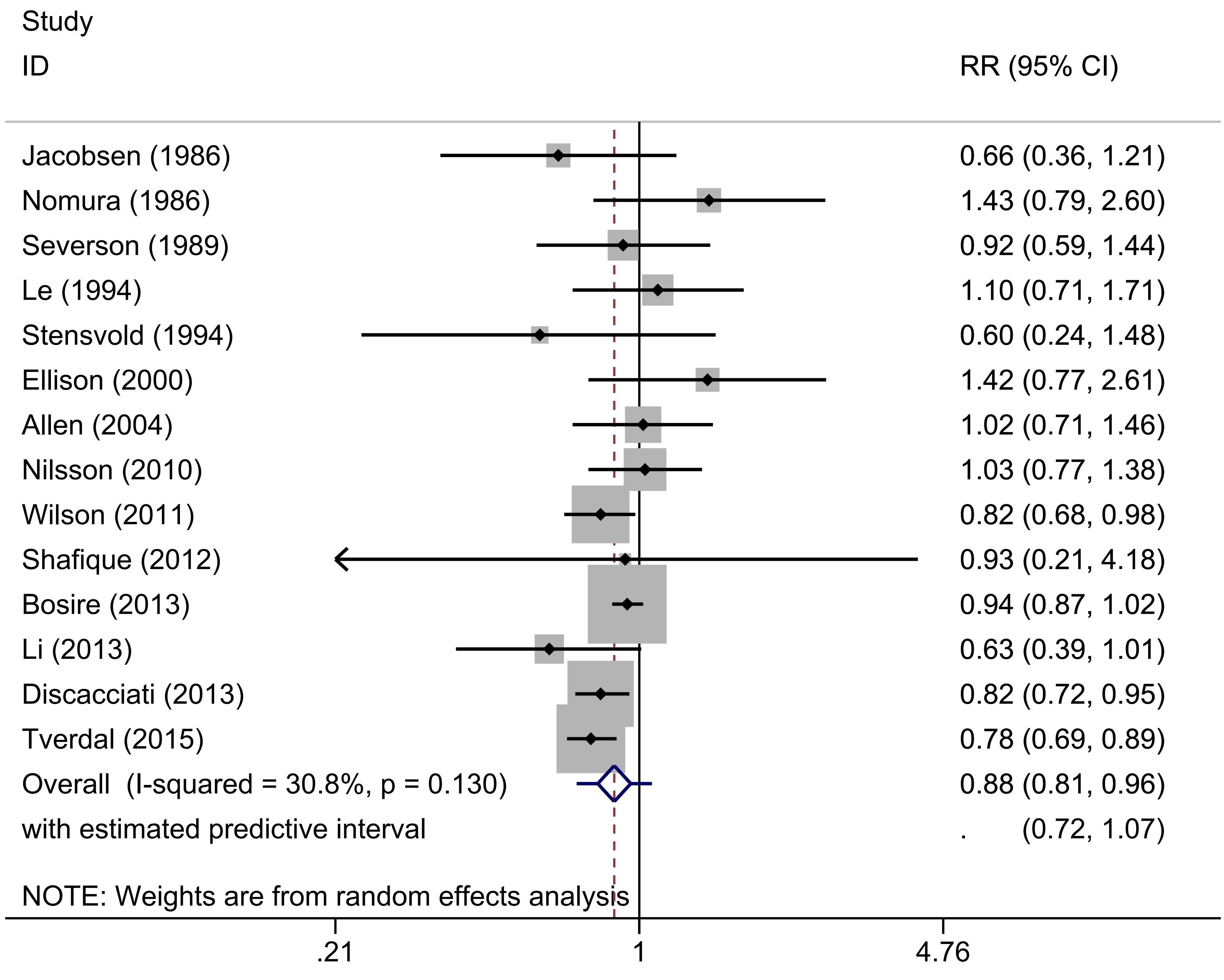

Supplementary figure 66, Rectal cancer, H/L, only cohort studies

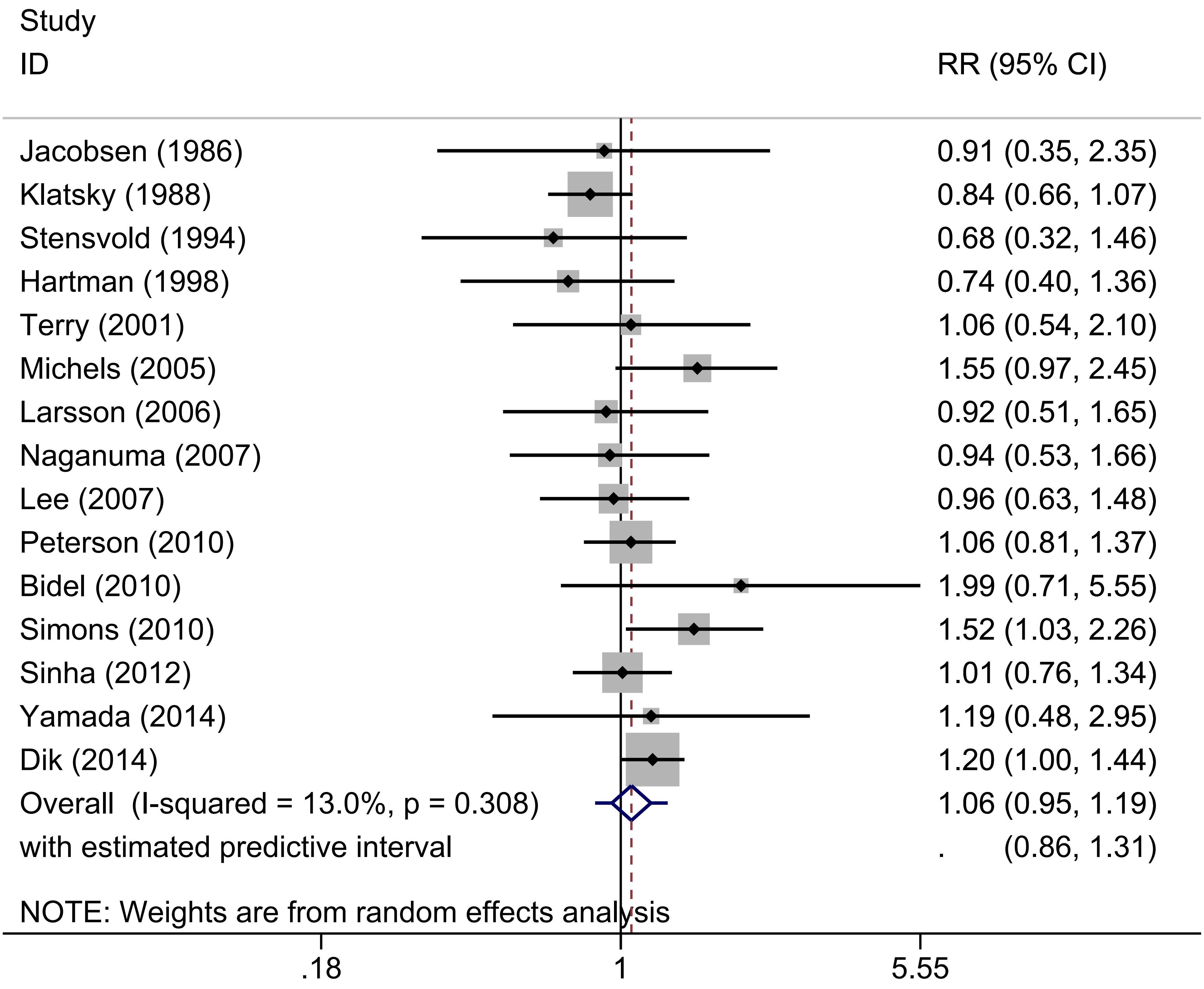

Supplementary figure 67, Rectal cancer, Per 1 cup, only cohort studies

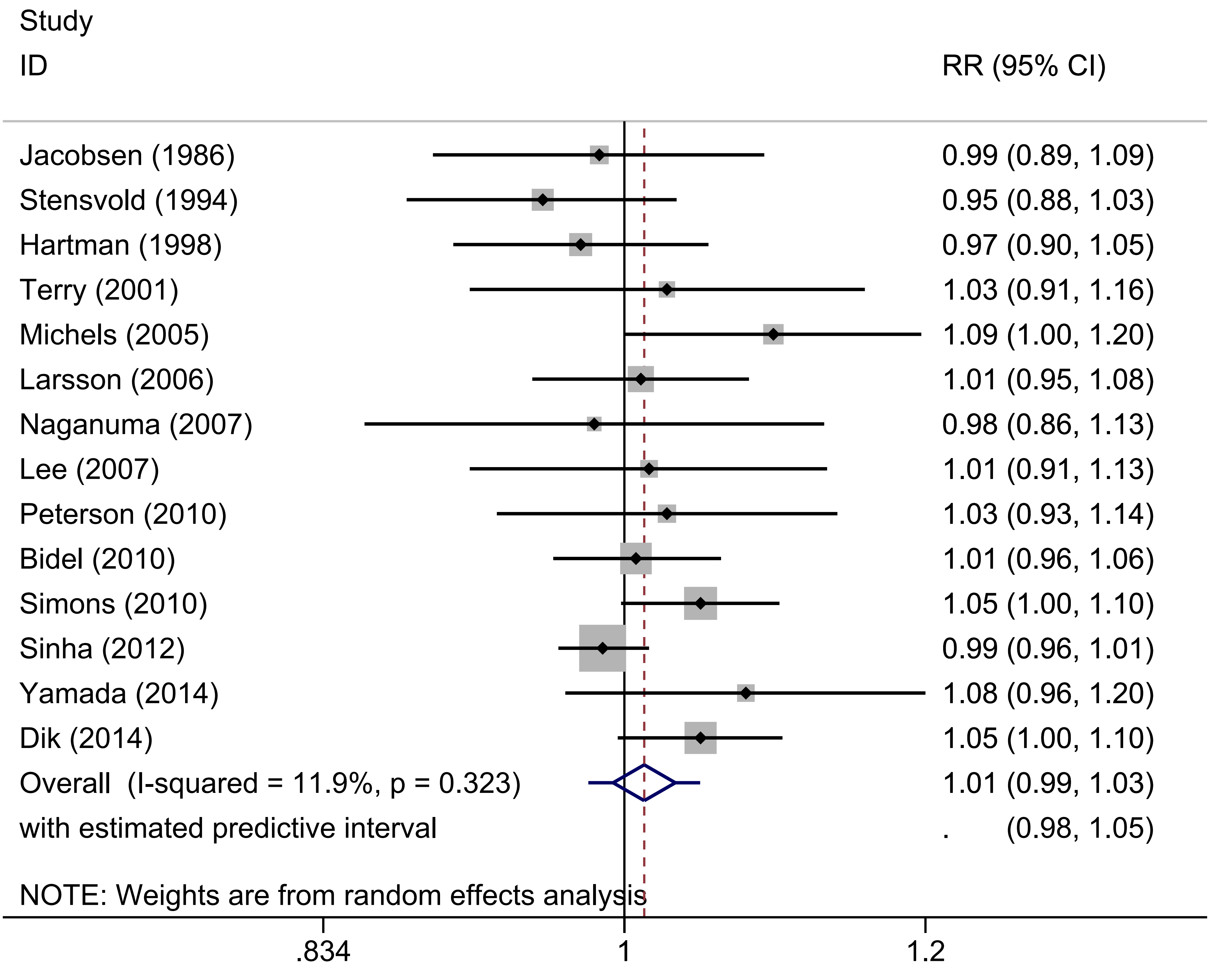

Supplementary figure 68, Thyroid cancer, H/L, only cohort studies

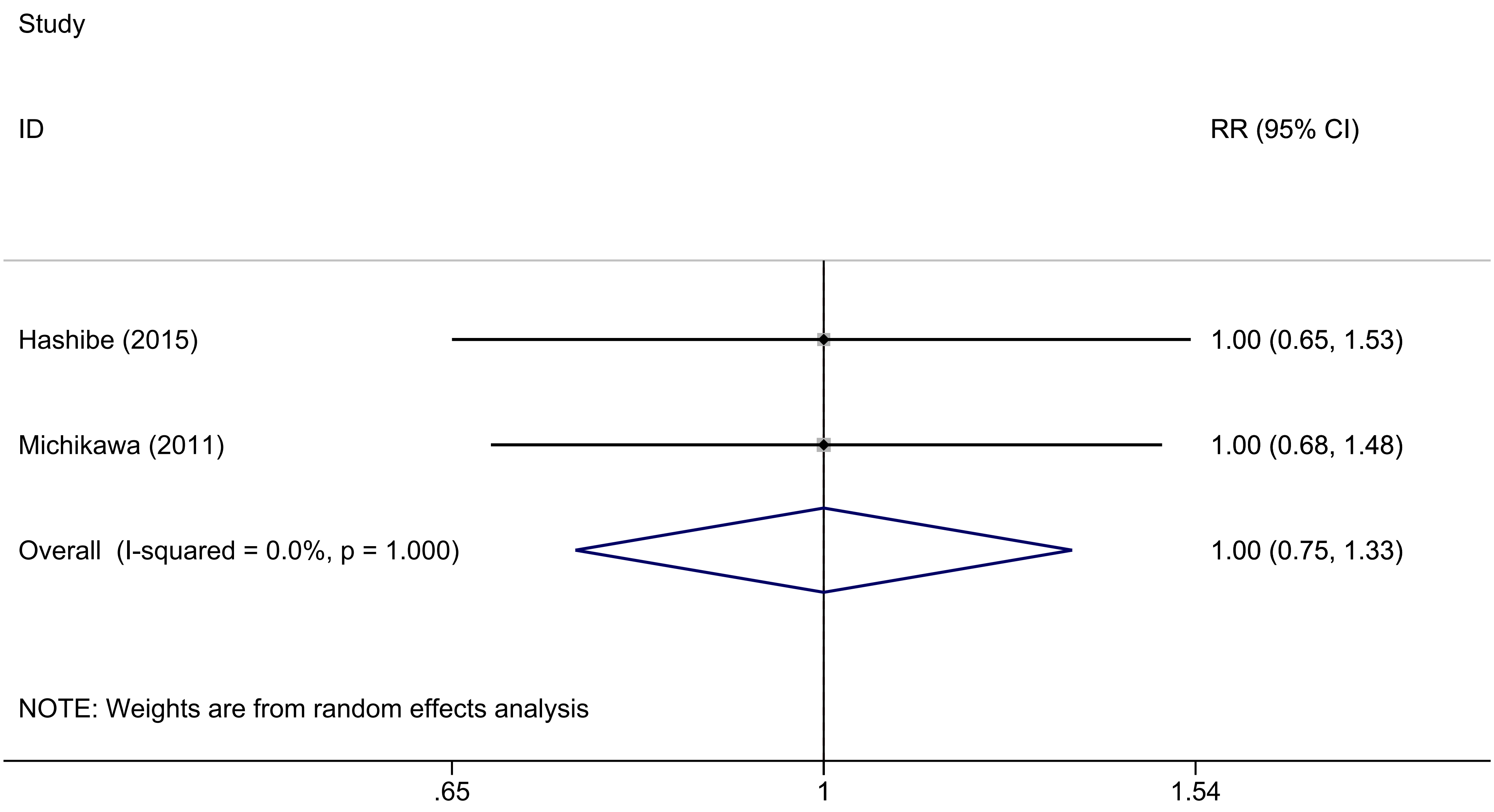

Supplementary figure 69, Biliary tract cancer, H/L, only cohort studies

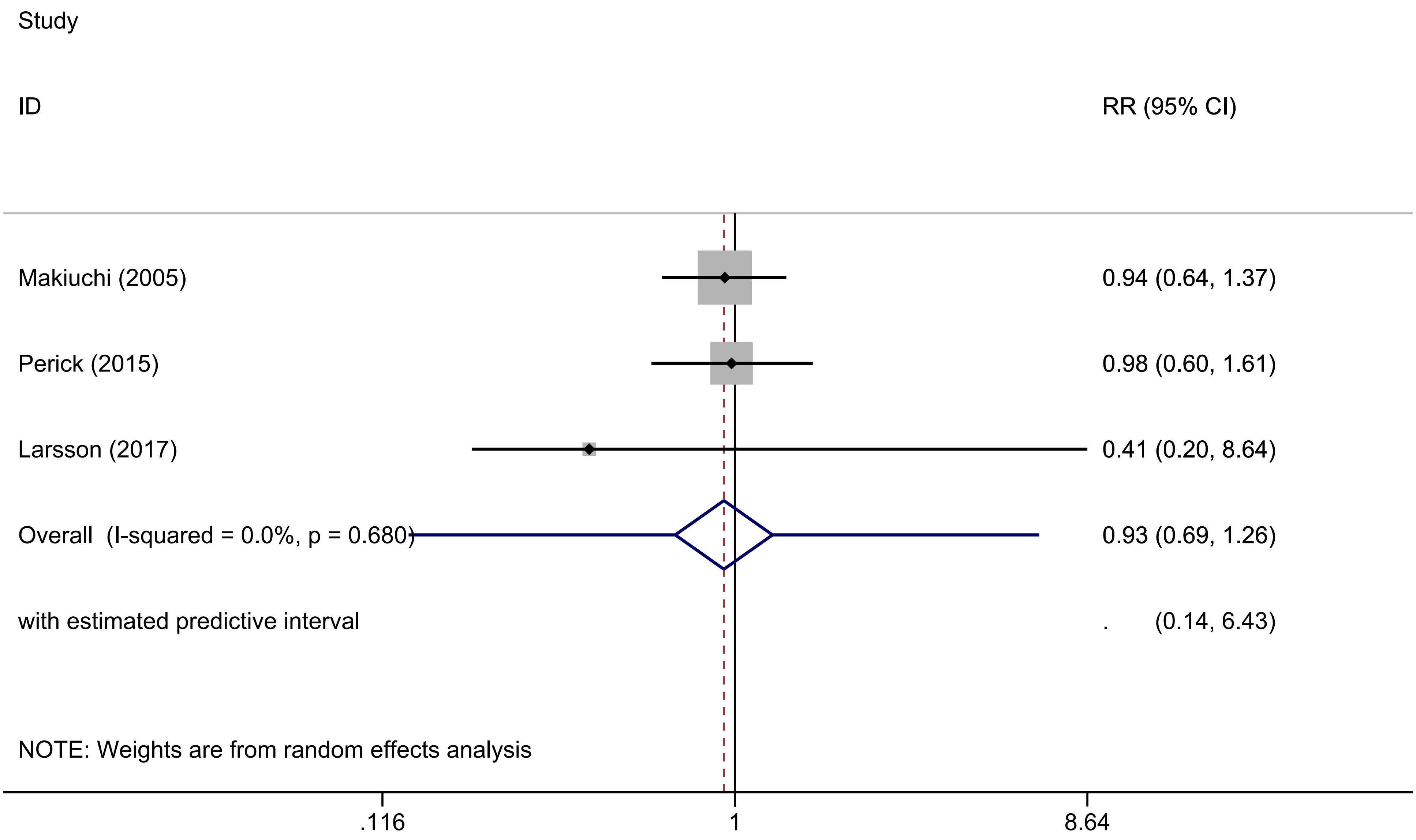

Supplementary figure 70, Glioma, Per 1 cup, only cohort studies

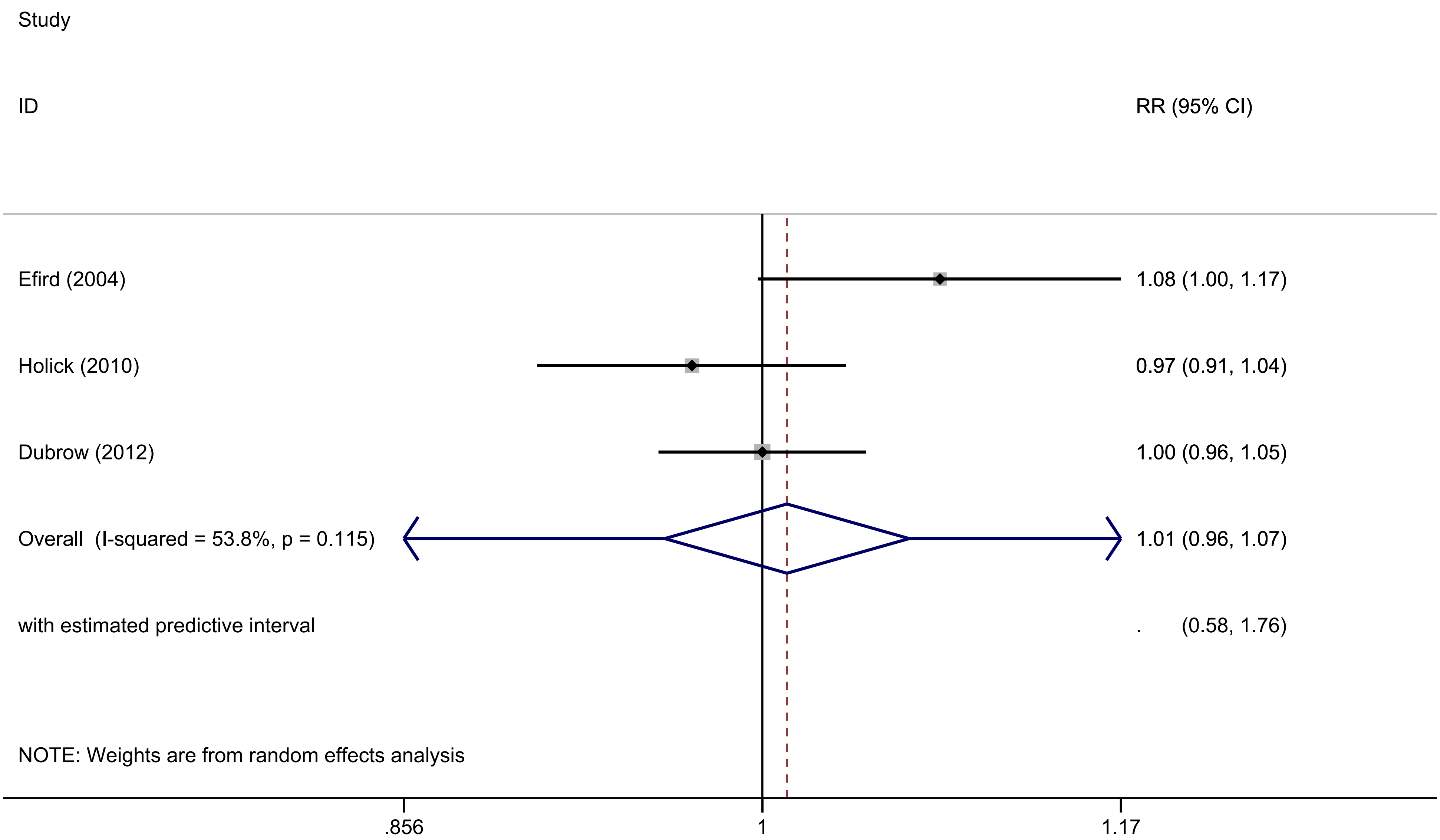

Supplement: Supplementary file 2 — Additional file 2: Figure S1–S36. Forest plots of all observational studies. Figure S37–S70. Forest plots of cohort studies. [file 12885_2020_6561_MOESM2_ESM.pdf]
